# Supplementary material for: Comparison of Cinchona Catalysts Containing Ethyl or Vinyl or Ethynyl Group at Their Quinuclidine Ring
Source: Materials (Basel). 2019 Sep 18;12(18):3034. doi: 10.3390/ma12183034 (PMC6766286; doi:10.3390/ma12183034)
Supplement: Supplementary file 1 [file materials-12-03034-s001.pdf]

materials

Article

Comparison of Cinchona Catalysts Containing Ethyl or Vinyl or Ethynyl Group at Their Quinuclidine Ring

Sándor Nagy [1], Zsuzsanna Fehér [1], Gergő Dargó [1,2], Júlia Barabás [3], Zsófia Garádi [3], Béla Mátravölgyi [1], Péter Kisszékelyi [1], Gyula Dargó [1], Péter Huszthy [1], Tibor Höltzl [3,4], György Tibor Balogh [2,5,*] and József Kupai [1,*]

1 Department of Organic Chemistry & Technology, Budapest University of Technology & Economics, Szent Gellért tér 4, Budapest H-1111, Hungary; nagy.sandor@mail.bme.hu (S.N.); zsuzsi516@gmail.com (Z.F.); dageri15@gmail.com (G.D.); bmatravolgyi@mail.bme.hu (B.M.); pkisszekelyi@mail.bme.hu (P.K.); huszthy@mail.bme.hu (P.H.)
2 Chemical Department, Chemical Works of Gedeon Richter Plc., P.O. Box 27, Budapest H-1103, Hungary; gytbalogh@mail.bme.hu
3 Department of Inorganic & Analytical Chemistry, Budapest University of Technology & Economics, Szent Gellért tér 4, Budapest H-1111, Hungary; julia.barabas@mail.bme.hu (J.B.); garadi.zsofia@egis.hu (Z.G.); tibor.holtzl@furukawaelectric.com (T.H.)
4 Furukawa Electric Institute of Technology, Késmárk utca 28/A, Budapest H-1158, Hungary
5 Department of Chemical & Environmental Process Engineering, Budapest University of Technology and Economics, Műegyetem rkp. 3., Budapest H-1111, Hungary
* Correspondence address: jkupai@mail.bme.hu, Tel.: +36-1-463-2229 (J.K.); gytbalogh@mail.bme.hu, Tel.: +36-1-463-2174 (G.T.B.)

Received: 22 August 2019: Accepted: 12 September 2019; Published: date

1. The pK_a Values of Catalysts Measured in Different Solvents

Table S1. The measured pK_a values of OH derivatives of cinchona in six different solvents.

	**HQ**				**Q**				**DQ**		
**Water**	**pK_{a1}**		**pK_{a2}**		**pK_{a1}**		**pK_{a2}**		**pK_{a1}**	**pK_{a2}**	
	**4.36**		**9.10**		**4.34**		**8.52**		**4.34**	**7.40**	
**0% MeOH**	**pK_{a1}**		**pK_{a2}**		**pK_{a1}**		**pK_{a2}**		**pK_{a1}**	**pK_{a2}**	
Yasuda–Shedlovsky	4.27		9.20		4.24		8.66		4.23	7.53	
Linear	4.43		9.32		4.40		8.76		4.41	7.61	
**98.0% MeOH**	**pK_{a1}**		**pK_{a2}**		**pK_{a1}**		**pK_{a2}**		**pK_{a1}**	**pK_{a2}**	
Yasuda–Shedlovsky	2.45		8.01		2.40		7.74		2.20	6.93	
Linear	2.77		8.12		2.73		7.74		2.60	6.81	
**99.99% MeOH**	**pK_{a1}**		**pK_{a2}**		**pK_{a1}**		**pK_{a2}**		**pK_{a1}**	**pK_{a2}**	
Yasuda–Shedlovsky	4.55		10.15		4.5		9.89		4.29	9.10	
Linear	**2.74**		**8.09**		**2.69**		**7.72**		**2.56**	**6.90**	
**0% MeCN**	**pK_{a1}**		**pK_{a2}**		**pK_{a1}**		**pK_{a2}**		**pK_{a1}**	**pK_{a2}**	
Yasuda–Shedlovsky	3.99		9.17		3.97		8.6		3.94	7.5	
Linear	4.1		9.22		4.09		8.64		4.06	7.54	
**98.0% MeCN**	**pK_{a1}**		**pK_{a2}**		**pK_{a1}**		**pK_{a2}**		**pK_{a1}**	**pK_{a2}**	
Yasuda–Shedlovsky	4.28		10.15		4.2		9.76		4.24	8.62	
Linear	3.17		9.04		3.1		8.64		3.13	7.51	
**99.99% MeCN**	**pK_{a1}**		**pK_{a2}**		**pK_{a1}**		**pK_{a2}**		**pK_{a1}**	**pK_{a2}**	
Yasuda–Shedlovsky	6.6		12.46		6.53		12.07		6.56	10.94	
Linear	**3.15**		**9.03**		**3.08**		**8.64**		**3.11**	**7.51**	
**0% Dioxane**	**pK_{a1}**	**pK_{a2}**	**pK_{a3}**	**pK_{a4}**	**pK_{a1}**	**pK_{a2}**	**pK_{a3}**	**pK_{a4}**	**pK_{a1}**	**pK_{a2}**	**pK_{a3}**
Yasuda–Shedlovsky	3.86	6.92	9.14	11.08	3.78	6.82	8.62	11.25	3.79	7.14	11.28
Linear	4.17	6.91	9.27	10.85	4.1	6.75	8.76	11.12	4.13	7.17	11.17
**98.0% Dioxane**	**pK_{a1}**	**pK_{a2}**	**pK_{a3}**	**pK_{a4}**	**pK_{a1}**	**pK_{a2}**	**pK_{a3}**	**pK_{a4}**	**pK_{a1}**	**pK_{a2}**	**pK_{a3}**
Yasuda–Shedlovsky	−12.35	6.20	1.45	20.79	−11.14	8.01	1.67	15.65	−12.64	4.81	14.9
Linear	2.68	7.32	8.82	12.54	2.70	7.42	8.36	12.18	2.58	7.36	12.12
**99.99% Dioxane**	**pK_{a1}**	**pK_{a2}**	**pK_{a3}**	**pK_{a4}**	**pK_{a1}**	**pK_{a2}**	**pK_{a3}**	**pK_{a4}**	**pK_{a1}**	**pK_{a2}**	**pK_{a3}**
Yasuda–Shedlovsky	−16.44	7.64	0.4	25.96	−14.76	10.41	0.9	18.92	−16.8	5.68	17.89
Linear	**2.65**	7.33	**8.81**	12.57	**2.67**	7.43	**8.35**	12.20	**2.55**	**7.37**	12.14
**0% THF**	**pK_{a1}**		**pK_{a2}**	**pK_{a3}**	**pK_{a1}**		**pK_{a2}**	**pK_{a3}**	**pK_{a1}**	**pK_{a2}**	**pK_{a3}**
Yasuda–Shedlovsky	4.02		3.23	11.82	3.98		8.74	11.78	3.95	7.57	11.74
Linear	4.55		9.75	11.94	4.51		9.23	11.88	4.49	7.96	11.83
**98.0% THF**	**pK_{a1}**		**pK_{a2}**	**pK_{a3}**	**pK_{a1}**		**pK_{a2}**	**pK_{a3}**	**pK_{a1}**	**pK_{a2}**	**pK_{a3}**
Yasuda–Shedlovsky	−4.77		0.68	10.56	−5.21		0.30	10.83	−5.02	1.24	11.00
Linear	1.30		6.59	11.53	1.17		6.19	11.60	1.17	5.65	11.62
**99.99% THF**	**pK_{a1}**		**pK_{a2}**	**pK_{a3}**	**pK_{a1}**		**pK_{a2}**	**pK_{a3}**	**pK_{a1}**	**pK_{a2}**	**pK_{a3}**
Yasuda–Shedlovsky	−3.09		2.38	12.69	−3.55		2.01	12.97	−3.35	3.07	13.16
Linear	**1.23**		**6.52**	11.52	**1.10**		**6.13**	11.60	**1.10**	**5.60**	11.61
**0% DMSO**	**pK_{a1}**		**pK_{a2}**		**pK_{a1}**		**pK_{a2}**		**pK_{a1}**	**pK_{a2}**	

Yasuda–Shedlovsky	3.99	9.07	3.95	8.52	3.91	7.29
Linear	4.37	9.29	4.36	8.72	4.35	7.47
**98.0% DMSO**	**p*K*a1**	**p*K*a2**	**p*K*a1**	**p*K*a2**	**p*K*a1**	**p*K*a2**
Yasuda–Shedlovsky	−3.04	5.24	−3.33	5.14	−3.70	4.67
Linear	2.30	8.22	2.17	7.77	2.02	6.74
**99.99% DMSO**	**p*K*a1**	**p*K*a2**	**p*K*a1**	**p*K*a2**	**p*K*a1**	**p*K*a2**
Yasuda–Shedlovsky	−1.55	7.03	−1.86	6.97	−2.26	6.58
Linear	**2.26**	**8.2**	**2.13**	**7.76**	**1.97**	**6.72**

Table S2. The measured p*K*a values of cinchona amine derivatives in six different solvents.

	HQ–N					Q–N					DQ–N				
**Water**	**p*K*a1**	**p*K*a2**		**p*K*a3**		**p*K*a1**	**p*K*a2**		**p*K*a3**		**p*K*a1**	**p*K*a2**		**p*K*a3**	
	**2.07**	**4.47**		**9.71**		**2.02**	**4.42**		**9.10**		**1.93**	**4.34**		**8.10**	
**0% MeOH**	**p*K*a1**	**p*K*a2**		**p*K*a3**		**p*K*a1**	**p*K*a2**		**p*K*a3**		**p*K*a1**	**p*K*a2**		**p*K*a3**	
Yasuda–Shedlovsky	1.98	4.28		9.65		1.94	4.25		9.07		1.87	4.16		8.00	
Linear	2.11	4.47		9.84		2.07	4.45		9.24		2.01	4.38		8.13	
**98.0% MeOH**															
Yasuda–Shedlovsky	1.05	2.74		8.02		1.05	2.61		7.65		0.87	2.19		7.17	
Linear	1.02	2.92		8.24		1.00	2.82		2.79		0.87	2.53		7.09	
**99.99% MeOH**															
Yasuda–Shedlovsky	3.20	4.86		10.13		3.20	4.72		9.78		3.02	4.29		9.32	
**Linear**	**1.00**	**2.89**		**8.21**		**0.98**	**2.79**		**7.76**		**0.84**	**2.49**		**7.07**	
**0% MeCN**	**p*K*a1**	**p*K*a2**		**p*K*a3**		**p*K*a1**	**p*K*a2**		**p*K*a3**		**p*K*a1**	**p*K*a2**		**p*K*a3**	
Yasuda–Shedlovsky	1.92	4.02		9.49		1.92	4.00		8.93		1.83	3.89		7.83	
Linear	2.07	4.15		9.55		2.08	4.14		9.00		1.98	4.02		7.86	
**98.0% MeCN**															
Yasuda–Shedlovsky	1.74	4.15		10.33		1.62	4.04		9.73		1.53	3.97		9.00	
Linear	0.67	3.05		9.22		0.55	2.94		8.62		0.47	2.86		7.88	
**99.99% MeCN**															
Yasuda–Shedlovsky	4.07	6.47		12.65		3.95	6.37		12.05		3.86	6.29		11.31	
**Linear**	**0.64**	**3.02**		**9.21**		**0.52**	**2.91**		**8.61**		**0.43**	**2.84**		**7.88**	
**0% Dioxane**	**p*K*a1**	**p*K*a2**	**p*K*a3**	**p*K*a4**	**p*K*a5**	**p*K*a1**	**p*K*a2**	**p*K*a3**	**p*K*a4**	**p*K*a5**	**p*K*a1**	**p*K*a2**	**p*K*a3**	**p*K*a4**	**p*K*a5**
Yasuda–Shedlovsky	1.8	3.76	6.76	9.55	11.29	1.7	3.69	6.3	8.94	11.4	1.67	3.63	6.47	7.81	11.22
Linear	2.01	3.93	6.62	9.77	11.2	1.84	3.86	5.75	9.1	11.33	1.78	3.86	6.14	7.69	11.07
**98.0% Dioxane**															
Yasuda–Shedlovsky	−5.04	−1.91	10.32	1.23	14.22	−2.66	−1.37	19.52	3.33	12.75	−2.38	−3.62	15.52	11.64	16.36
Linear	1.47	3.56	7.65	9.09	12.06	1.64	3.54	8.37	8.77	12.01	1.68	3.25	8.00	8.68	12.22
**99.99% Dioxane**															
Yasuda–Shedlovsky	−5.78	−2.23	13.29	−0.04	16.96	−2.51	−1.47	25.94	3.03	14.79	−2.13	−4.51	20.45	14.71	19.89
**Linear**	**1.46**	**3.55**	**7.67**	**9.07**	**12.08**	**1.63**	**3.53**	**8.42**	**8.76**	**12.02**	**1.67**	**3.24**	**8.03**	**8.70**	**12.24**
**0% THF**	**p*K*a1**	**p*K*a2**		**p*K*a3**	**p*K*a4**	**p*K*a1**	**p*K*a2**		**p*K*a3**	**p*K*a4**	**p*K*a1**	**p*K*a2**		**p*K*a3**	**p*K*a4**
Yasuda–Shedlovsky	2.09	4.19		9.85	11.87	2.15	4.1		9.2	11.86	2.13	4.04		8.31	11.79
Linear	2.32	4.62		10.34	12	2.4	4.49		9.57	11.99	2.47	4.49		8.69	11.89

**98.0% THF**															
Yasuda–Shedlovsky	−1.15	−2.74		1.65	10.26	−1.32	−2.33		3.04	10.34	−3.48	−3.47		2.2	10.79
Linear	1.16	2.06		7.33	11.46	1.12	2.13		7.33	11.49	0.45	1.73		6.44	11.59
**99.99% THF**															
Yasuda–Shedlovsky	0.86	−0.95		3.37	12.36	0.68	−0.5		4.88	12.45	−1.61	−1.71		4.05	12.94
**Linear**	**1.13**	**2.01**		**7.26**	**11.45**	**1.09**	**2.08**		**7.29**	**11.47**	**0.41**	**1.67**		**6.4**	**11.58**
**0% DMSO**	**p*K*a1**	**p*K*a2**		**p*K*a3**		**p*K*a1**	**p*K*a2**		**p*K*a3**		**p*K*a1**	**p*K*a2**		**p*K*a3**	
Yasuda–Shedlovsky	2.01	4.08		9.33		1.97	4.04		8.84		1.92	3.99		7.89	
Linear	2.21	4.34		9.47		2.17	4.33		9.01		2.12	4.35		8.04	
**98.0% DMSO**															
Yasuda–Shedlovsky	−1.44	−0.55		6.84		−1.42	−0.93		6.08		−1.38	−2.45		5.41	
Linear	1.25	2.99		8.84		1.23	2.87		8.27		1.21	2.44		7.39	
**99.99% DMSO**															
Yasuda–Shedlovsky	0.39	1.16		8.75		0.41	0.75		7.97		0.46	−0.91		7.33	
**Linear**	**1.23**	**2.97**		**8.82**		**1.21**	**2.84**		**8.25**		**1.19**	**2.41**		**7.38**	

Table S3. The measured p*K*a values of cinchona squaramides in six different solvents.

	**HQ–SQ**					**Q–SQ**				**DQ–SQ**			
**Water**	**p*K*a1**	**p*K*a2**	**p*K*a3**	**p*K*a4**	**p*K*a5**	**p*K*a1**	**p*K*a2**	**p*K*a3**	**p*K*a4**	**p*K*a1**	**p*K*a2**	**p*K*a3**	**p*K*a4**
	**3.50**	**6.68**	**7.86**	**9.57**	**11.73**	**3.23**	**6.95**	**9.29**		**3.21**	**6.13**	**9.20**	
**0% MeOH**	**p*K*a1**	**p*K*a2**	**p*K*a3**	**p*K*a4**	**p*K*a5**	**p*K*a1**	**p*K*a2**	**p*K*a3**		**p*K*a1**	**p*K*a2**	**p*K*a3**	
Yasuda–Shedlovsky	3.28	6.62	7.82	9.52	11.86	3.23	6.95	9.29		3.21	6.13	9.20	
Linear	3.50	6.68	7.86	9.57	11.73	3.45	7.12	9.29		3.43	6.30	9.19	
**98.0% MeOH**													
Yasuda–Shedlovsky	1.57	6.71	8.34	9.94	14.08	1.69	6.00	10.19		1.54	5.11	10.23	
Linear	1.82	6.30	7.77	9.40	12.93	1.83	5.95	9.49		1.74	5.08	9.48	
**99.99% MeOH**													
Yasuda–Shedlovsky	3.68	8.92	10.57	12.17	16.40	3.80	8.15	12.44		3.64	7.25	12.49	
**Linear**	**1.79**	**6.30**	**7.77**	**9.40**	**12.96**	**1.79**	**5.93**	**9.49**		**1.71**	**5.06**	**9.49**	
**0% MeCN**	**p*K*a1**		**p*K*a2**	**p*K*a3**		**p*K*a1**	**p*K*a2**	**p*K*a3**		**p*K*a1**	**p*K*a2**	**p*K*a3**	
Yasuda–Shedlovsky	3.06		7.61	9.38		2.95	7.17	9.18		2.91	6.17	9.08	
Linear	3.19		7.63	9.29		3.07	7.21	9.06		3.04	6.25	8.95	
**98.0% MeCN**													
Yasuda–Shedlovsky	3.14		8.85	12.01		3.17	8.27	12.15		3.04	6.90	12.22	
Linear	2.04		7.74	10.86		2.07	7.16	11.00		1.94	5.79	11.06	
**99.99% MeCN**													
Yasuda–Shedlovsky	5.47		11.16	14.30		5.50	10.58	14.44		5.36	9.21	14.51	
**Linear**	**2.01**		**7.74**	**10.89**		**2.05**	**7.16**	**11.04**		**1.91**	**5.78**	**11.10**	
**0% Dioxane**	**p*K*a1**		**p*K*a2**	**p*K*a3**		**p*K*a1**	**p*K*a2**	**p*K*a3**		**p*K*a1**	**p*K*a2**	**p*K*a3**	
Yasuda–Shedlovsky	2.91		7.28	9.22		2.80	6.88	9.16		2.64	5.49	9.10	
Linear	3.36		7.18	8.74		3.21	6.77	8.65		2.96	5.03	8.59	
**98.0% Dioxane**													
Yasuda–Shedlovsky	−13.31		8.89	24.70		−10.69	9.23	24.50		−7.93	18.35	24.34	

Linear	1.60	7.96	11.36		1.74	7.61	11.35		1.90	7.41	11.27	
**99.99% Dioxane**												
Yasuda−Shedlovsky	−17.40	11.17	31.93		−13.80	11.76	31.67		−10.00	24.63	31.47	
**Linear**	**1.57**	**7.97**	**11.41**		**1.71**	**7.63**	**11.40**		**1.88**	**7.46**	**11.33**	
**0% THF**	**p*K*a1**	**p*K*a2**	**p*K*a3**	**p*K*a4**	**p*K*a1**	**p*K*a2**	**p*K*a3**	**p*K*a4**	**p*K*a1**	**p*K*a2**	**p*K*a3**	**p*K*a4**
Yasuda−Shedlovsky	3.02	7.55	9.07	11.89	2.98	7.12	8.83	11.88	2.90	6.06	8.73	11.87
Linear	3.49	7.89	9.08	12.03	3.48	7.49	8.75	12.02	3.41	6.49	8.60	12.01
**98.0% THF**												
Yasuda−Shedlovsky	−5.71	1.39	10.54	9.93	−5.61	0.85	12.05	10.10	−5.68	−0.90	12.62	10.13
Linear	0.39	5.76	9.58	11.41	0.36	5.24	9.91	11.42	0.27	3.94	10.04	11.43
**99.99% THF**												
Yasuda−Shedlovsky	−4.02	3.23	12.83	12.02	−3.91	2.69	14.44	12.20	−3.98	0.89	15.05	12.23
**Linear**	**0.33**	**5.72**	**9.59**	**11.39**	**0.30**	**5.20**	**9.93**	**11.41**	**0.20**	**3.89**	**10.07**	**11.42**
**0% DMSO**	**p*K*a1**	**p*K*a2**	**p*K*a3**		**p*K*a1**	**p*K*a2**	**p*K*a3**		**p*K*a1**	**p*K*a2**	**p*K*a3**	
Yasuda−Shedlovsky	3.10	7.46	9.35		3.11	7.10	9.02		3.07	6.14	8.89	
Linear	3.55	7.79	9.61		3.52	7.42	9.12		3.47	6.39	8.94	
**98.0% DMSO**												
Yasuda−Shedlovsky	−4.09	2.20	5.75		−4.25	2.10	7.97		−4.34	1.52	8.46	
Linear	1.28	6.17	8.49		1.32	5.87	8.86		1.28	5.08	8.92	
**99.99% DMSO**												
Yasuda−Shedlovsky	−2.61	3.86	7.57		−2.78	3.78	10.02		−2.88	3.23	10.57	
**Linear**	**1.23**	**6.13**	**8.47**		**1.27**	**5.84**	**8.85**		**1.23**	**5.06**	**8.92**	

Table S4. The measured p*K*a values of cinchona thioureas in six different solvents.

	**HQ–TU**			**Q–TU**			**DQ–TU**		
	**p*K*a1**	**p*K*a2**	**p*K*a3**	**p*K*a1**	**p*K*a2**	**p*K*a3**	**p*K*a1**	**p*K*a2**	**p*K*a3**
**Water**	**3.38**	**8.61**	**10.87**	**3.42**	**8.06**	**10.84**	**3.44**	**7.02**	**10.78**
**0% MeOH**									
Yasuda−Shedlovsky	3.38	8.61	10.87	3.42	8.06	10.84	3.44	7.02	10.78
Linear	3.62	8.84	10.77	3.64	8.25	10.77	3.66	7.20	10.71
**98.0% MeOH**									
Yasuda−Shedlovsky	2.28	7.57	12.55	2.10	7.06	12.50	1.86	5.85	12.53
Linear	2.23	7.50	11.61	2.16	7.00	11.54	2.04	5.89	11.53
**99.99% MeOH**									
Yasuda−Shedlovsky	4.42	9.71	14.85	4.29	9.21	14.79	3.98	7.99	14.82
**Linear**	**2.20**	**7.47**	**11.63**	**2.13**	**6.98**	**11.56**	**2.00**	**5.86**	**11.55**
**0% MeCN**									
Yasuda−Shedlovsky	3.22	8.58	10.83	3.24	8.00	10.77	3.21	6.91	10.72
Linear	3.36	8.70	10.63	3.38	8.10	10.59	3.35	7.03	10.53
**98.0% MeCN**									
Yasuda−Shedlovsky	3.31	8.87	14.43	3.25	8.33	14.44	3.18	7.15	14.50
Linear	2.18	7.75	13.31	2.15	7.23	13.28	2.08	6.05	13.34
**99.99% MeCN**									

Yasuda–Shedlovsky	5.63	11.19	16.71	5.57	10.65	16.72	5.51	9.47	16.78
**Linear**	**2.15**	**7.73**	**13.36**	**2.12**	**7.21**	**13.33**	**2.05**	**6.03**	**13.39**
**0% Dioxane**									
Yasuda–Shedlovsky	3.15	7.69	11.03	3.11	7.74	10.96	3.20	7.79	11.15
Linear	3.73	7.65	10.55	3.65	8.10	10.42	3.74	8.57	11.00
**98.0% Dioxane**									
Yasuda–Shedlovsky	−11.96	8.04	22.55	−10.82	−1.90	24.11	−14.11	−19.48	18.32
Linear	1.79	8.19	12.88	1.89	7.04	12.99	1.71	5.26	12.30
**99.99% Dioxane**									
Yasuda–Shedlovsky	−15.65	9.87	28.36	−14.09	−3.64	30.50	−18.59	−27.51	22.58
**Linear**	**1.75**	**8.20**	**12.93**	**1.85**	**7.02**	**13.04**	**1.67**	**5.20**	**12.33**
**0% THF**									
Yasuda–Shedlovsky	2.96	7.95	11.06	2.87	7.47	11.03	2.64	6.79	10.87
Linear	3.50	8.44	10.74	3.37	8.02	10.72	3.50	7.56	10.44
**98.0% THF**									
Yasuda–Shedlovsky	−3.53	1.86	16.46	−3.03	0.80	16.28	−1.46	−3.14	17.69
Linear	0.81	5.95	13.00	0.91	5.26	12.93	1.22	3.48	13.32
**99.99% THF**									
Yasuda–Shedlovsky	−1.70	3.71	18.97	−1.17	2.61	18.79	0.31	−1.52	20.28
**Linear**	**0.75**	**5.90**	**13.05**	**0.86**	**5.20**	**12.97**	**1.18**	**3.40**	**13.38**
**0% DMSO**									
Yasuda–Shedlovsky	3.22	8.30	10.52	3.30	7.77	10.65	3.32	6.82	10.51
Linear	3.78	8.55	10.35	3.72	7.98	10.65	3.74	7.07	10.46
**98.0% DMSO**									
Yasuda–Shedlovsky	−2.98	5.87	13.17	−3.82	5.01	11.27	−4.65	2.29	12.10
Linear	1.45	7.67	11.44	1.52	7.14	10.95	1.37	5.78	11.07
**99.99% DMSO**									
Yasuda–Shedlovsky	−1.41	7.79	15.56	−2.34	6.90	13.48	−3.24	4.01	14.39
**Linear**	**1.41**	**7.66**	**11.46**	**1.47**	**7.12**	**10.96**	**1.33**	**5.76**	**11.09**

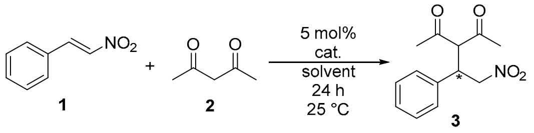

Scheme S1. Michael Addition of pentane-2,4-dione (**2**) to trans-β-nitrostyrene (**1**)

Table S5. Catalysts applied in Michael addition using *trans*–β–nitrostyrene (1) and pentane–2,4–dione (2)[a].

Entry	Catalyst	Solvent	Yield [%][b]	ee [%][c]
1	**HQ**	DCM	98	−16
2	**HQ**	MTBE	99	−8
3	**HQ**	toluene	98	−21
4	**HQ**	MeCN	97	4

5	**HQ**	EtOAc	99	−8
6	**HQ**	MeOH	91	−3
7	**HQ**	THF	96	−18
8	**Q**	DCM	98	−15
9	**Q**	MTBE	99	−6
10	**Q**	toluene	99	−14
11	**Q**	MeCN	97	1
12	**Q**	EtOAc	97	2
13	**Q**	MeOH	91	−2
14	**Q**	THF	93	−8
15	**DQ**	DCM	99	−13
16	**DQ**	MTBE	98	7
17	**DQ**	toluene	87	−15
18	**DQ**	MeCN	99	1
19	**DQ**	EtOAc	100	9
20	**DQ**	MeOH	82	0
21	**DQ**	THF	97	−7

Entry	Catalyst	Solvent	Yield [%][b]	ee [%][c]
1	**HQ-N**	DCM	99	6
2	**HQ-N**	MTBE	98	5
3	**HQ-N**	toluene	99	11
4	**HQ-N**	MeCN	99	4
5	**HQ-N**	EtOAc	99	6
6	**HQ-N**	MeOH	95	5
7	**HQ-N**	THF	97	15
8	**Q-N**	DCM	86	12
9	**Q-N**	MTBE	92	7
10	**Q-N**	toluene	71	11
11	**Q-N**	MeCN	91	7
12	**Q-N**	EtOAc	88	10
13	**Q-N**	MeOH	87	4
14	**Q-N**	THF	94	9
15	**DQ-N**	DCM	85	5
16	**DQ-N**	MTBE	83	7
17	**DQ-N**	toluene	89	34
18	**DQ-N**	MeCN	97	3
19	**DQ-N**	EtOAc	74	6
20	**DQ-N**	MeOH	87	3
21	**DQ-N**	THF	90	3

[a] Reaction conditions: Pentane−2,4−dione (**2**) (0.41 mmol) was added to the solution of *trans*−β−nitrostyrene (**1**) (0.16 mmol) and 5 mol% catalysts in 1 mL of solvent, then the resulting

mixture was stirred at room temperature for 24 hours. [b] Isolated yields. [c] Determined by chiral HPLC (plus marks *S* enantiomer, minus marks *R* enantiomer).

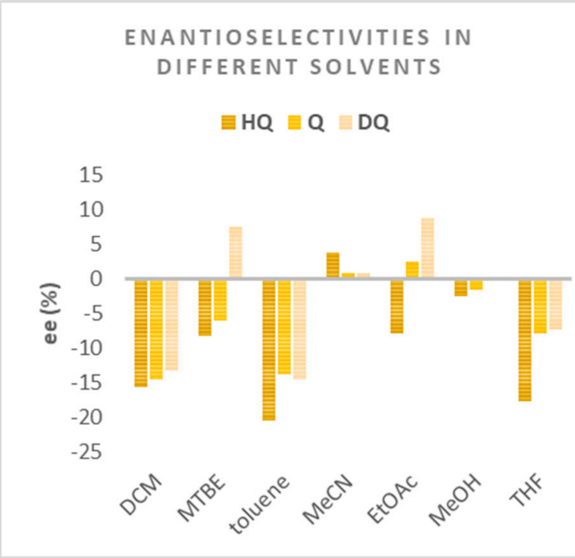

(**a**)

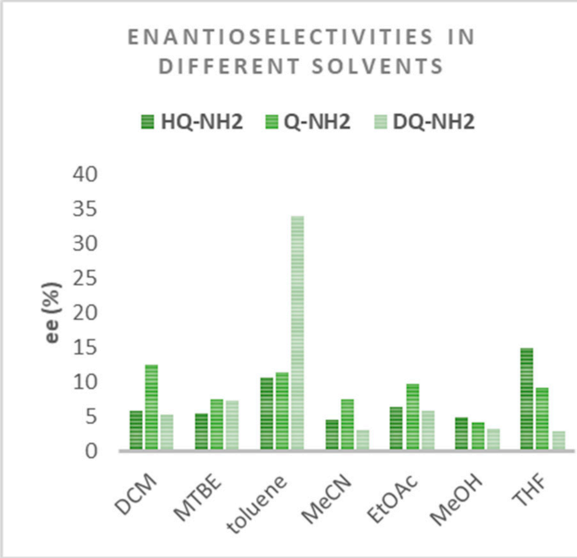

(**b**)

Figure S1. The dependence of enantiomeric excesses in different solvents when hydroxyl (a) or amino derivatives (b) were applied in Michael addition determined by chiral HPLC (plus marks *S* enantiomer, minus marks *R* enantiomer).

2. FT-IR Spectra of the New Compounds

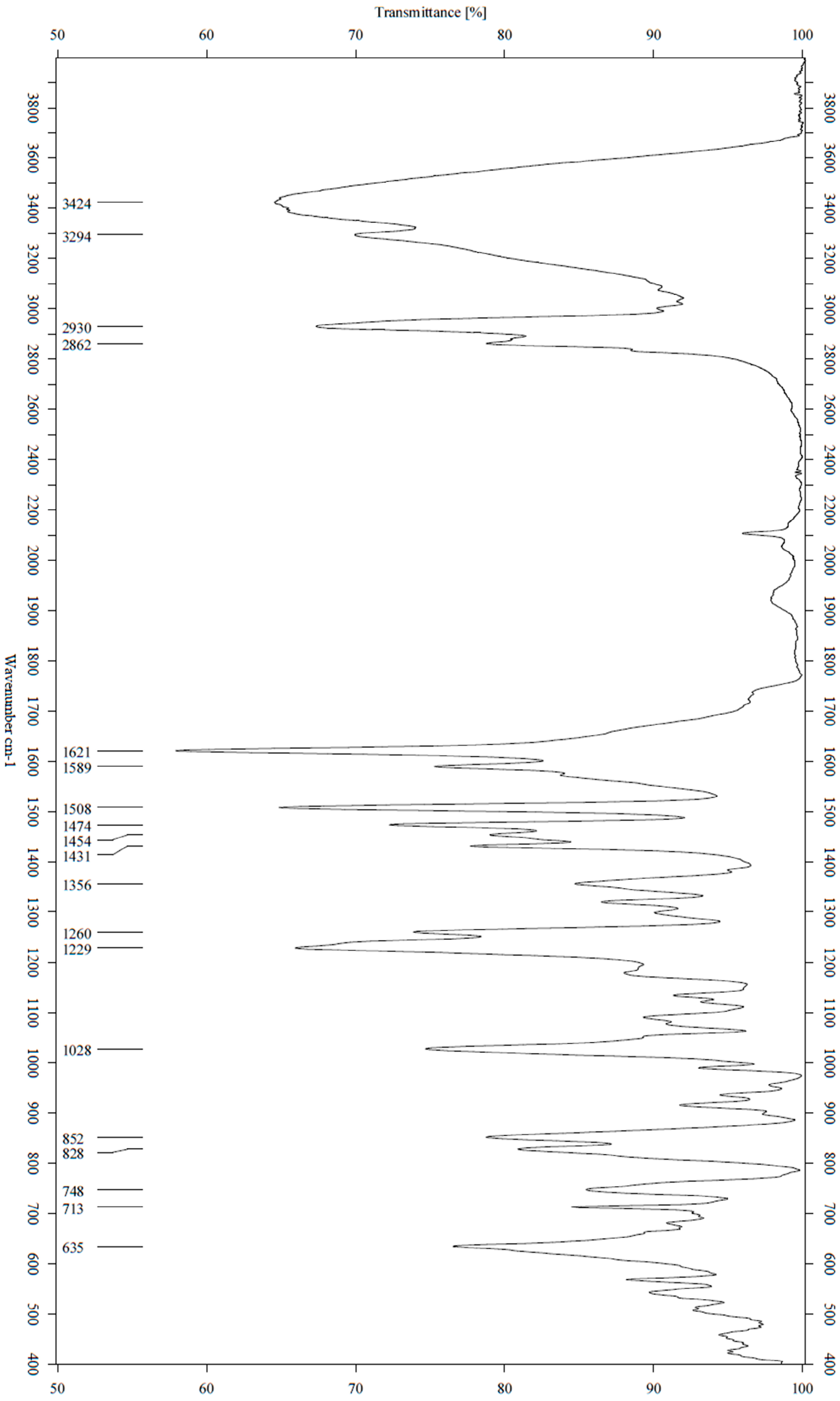

Figure S2. FT−IR Spectrum of **DQ-NH** (in KBr).

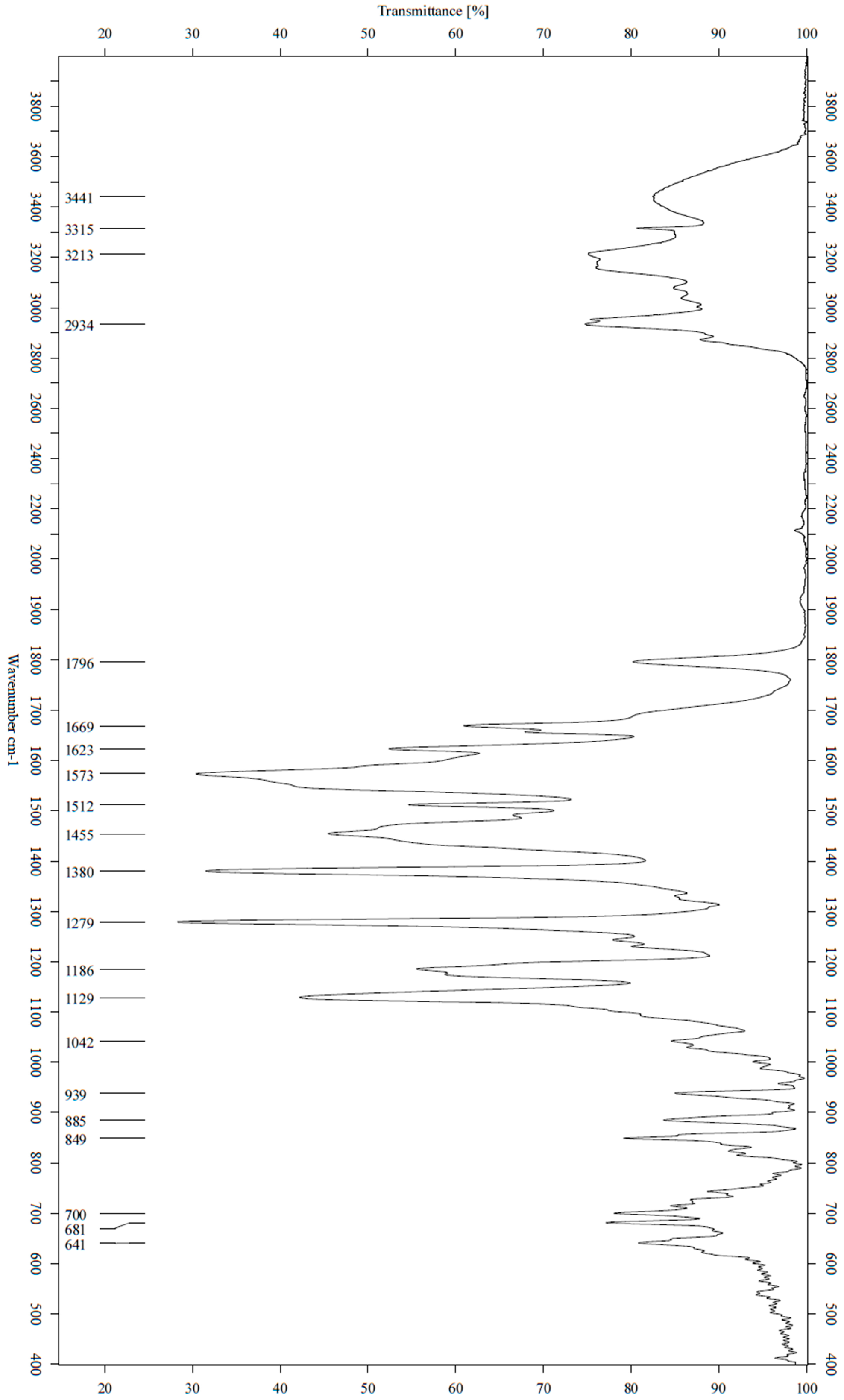

Figure S3. FT−IR Spectrum of **DQ-SQ** (in KBr).

3. NMR Spectra of the New Compounds

3.1. NMR Spectra of DQ-N

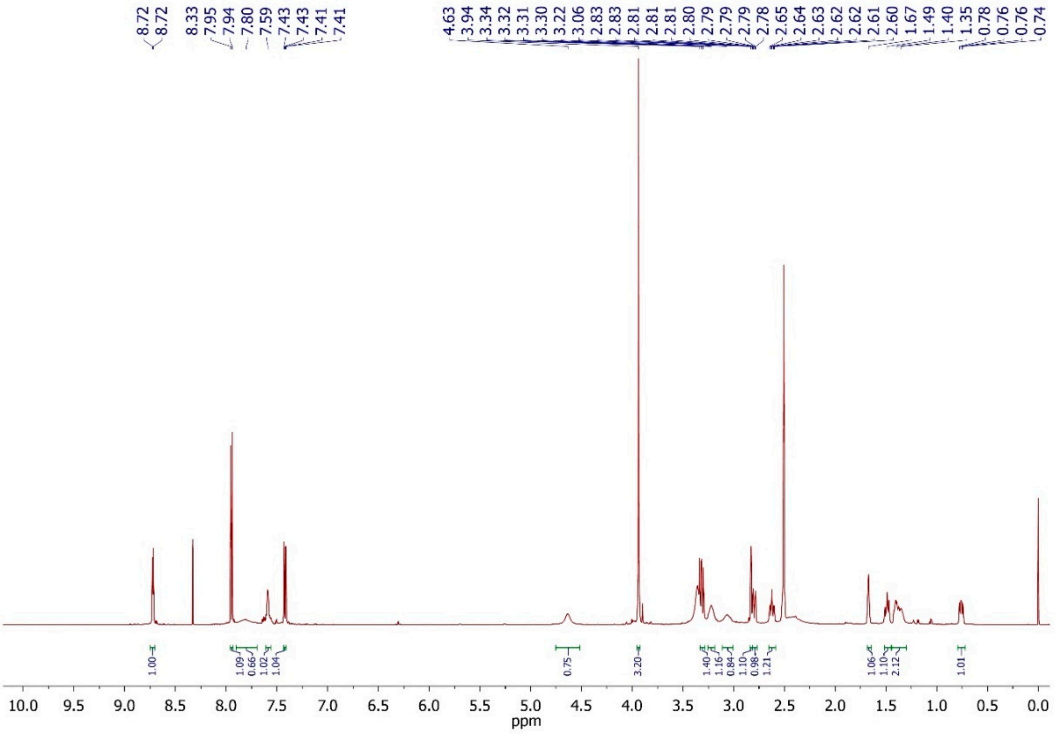

Figure S4a. ^{1}H NMR Spectrum of **DQ-N** (600 MHz, DMSO, 25 °C).

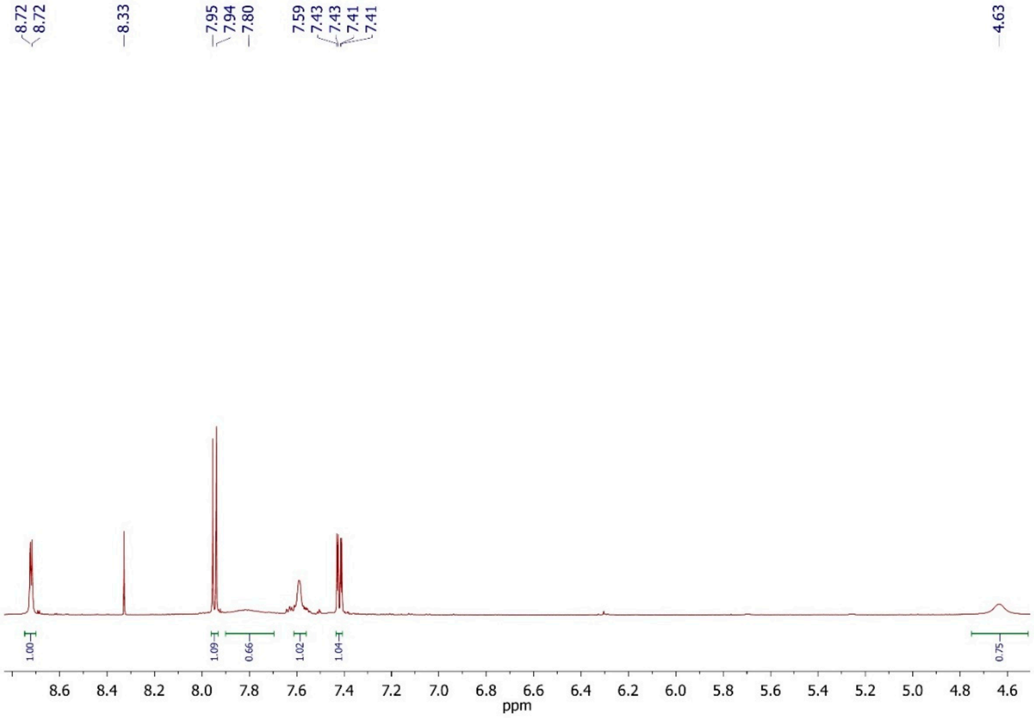

Figure S4b. ^{1}H NMR Spectrum of **DQ-N** in a range between 8.8 and 4.5 ppm (600 MHz, DMSO, 25°C).

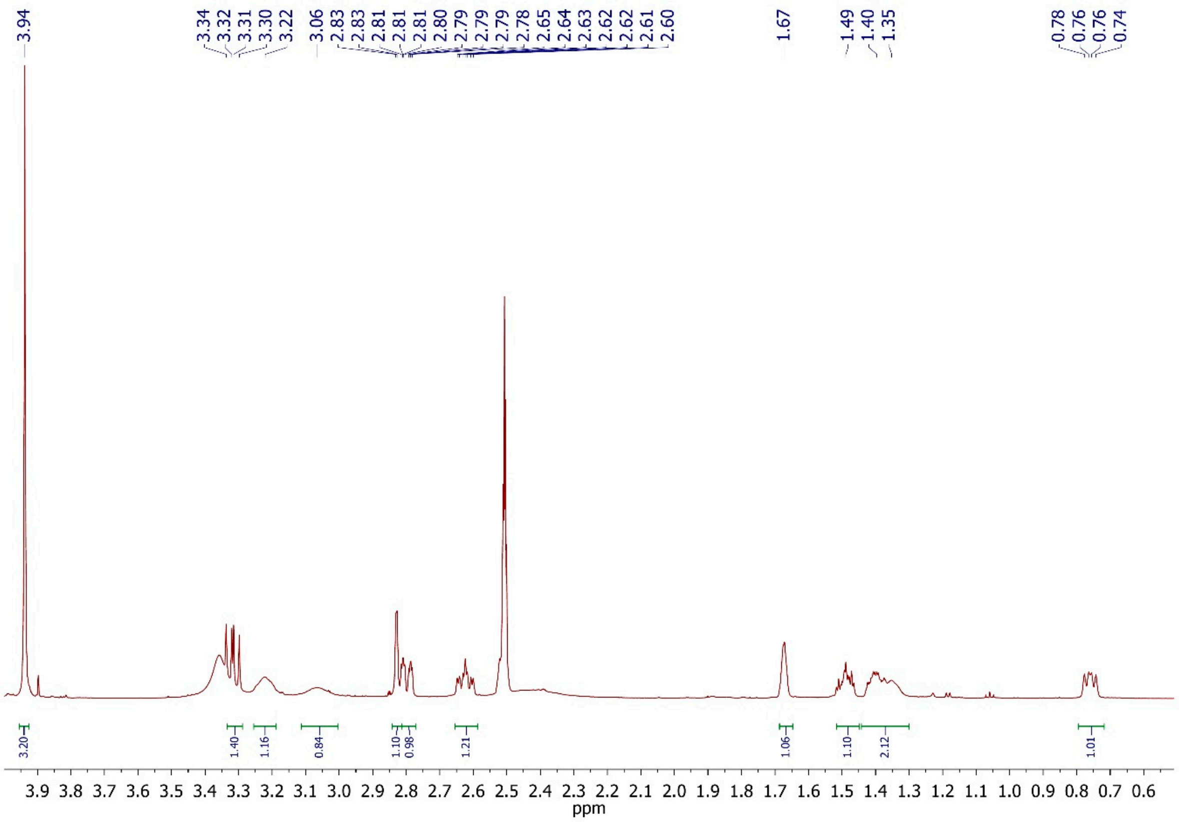

Figure S4c. ^{1}H NMR Spectrum of **DQ-N** in a range between 4.0 and 0.5 ppm (600 MHz, DMSO, 25°C).

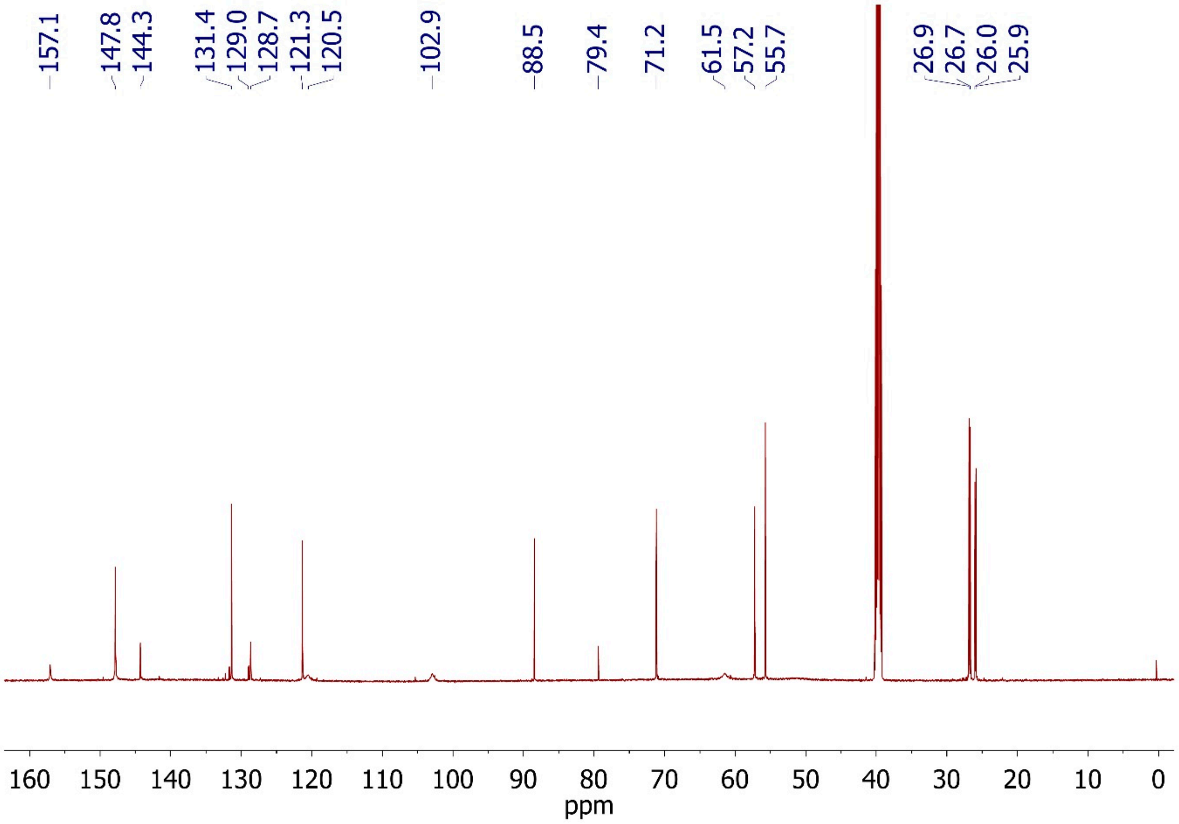

Figure S5. ^{13}C NMR Spectrum of **DQ–N** (150 MHz, DMSO, 25 °C).

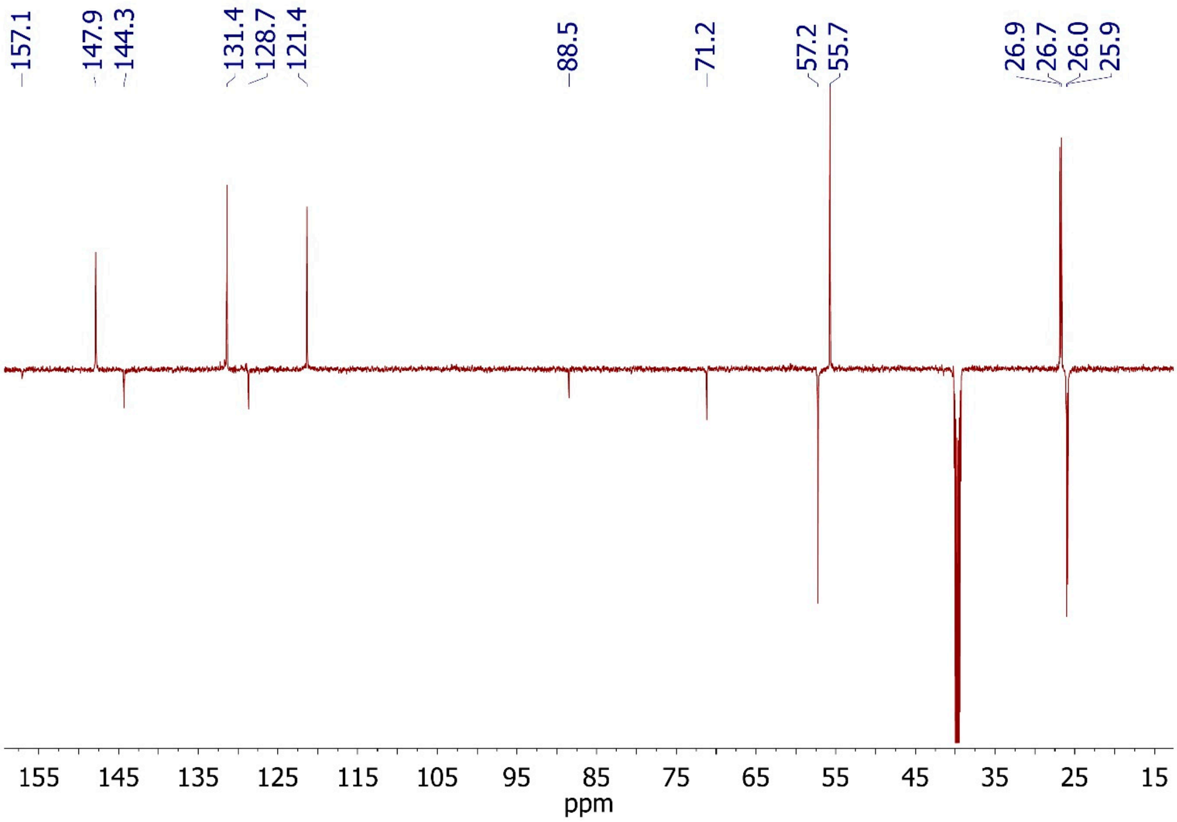

Figure S6. ^{13}C DEPT NMR Spectrum of **DQ-N** (150 MHz, DMSO, 25 °C).

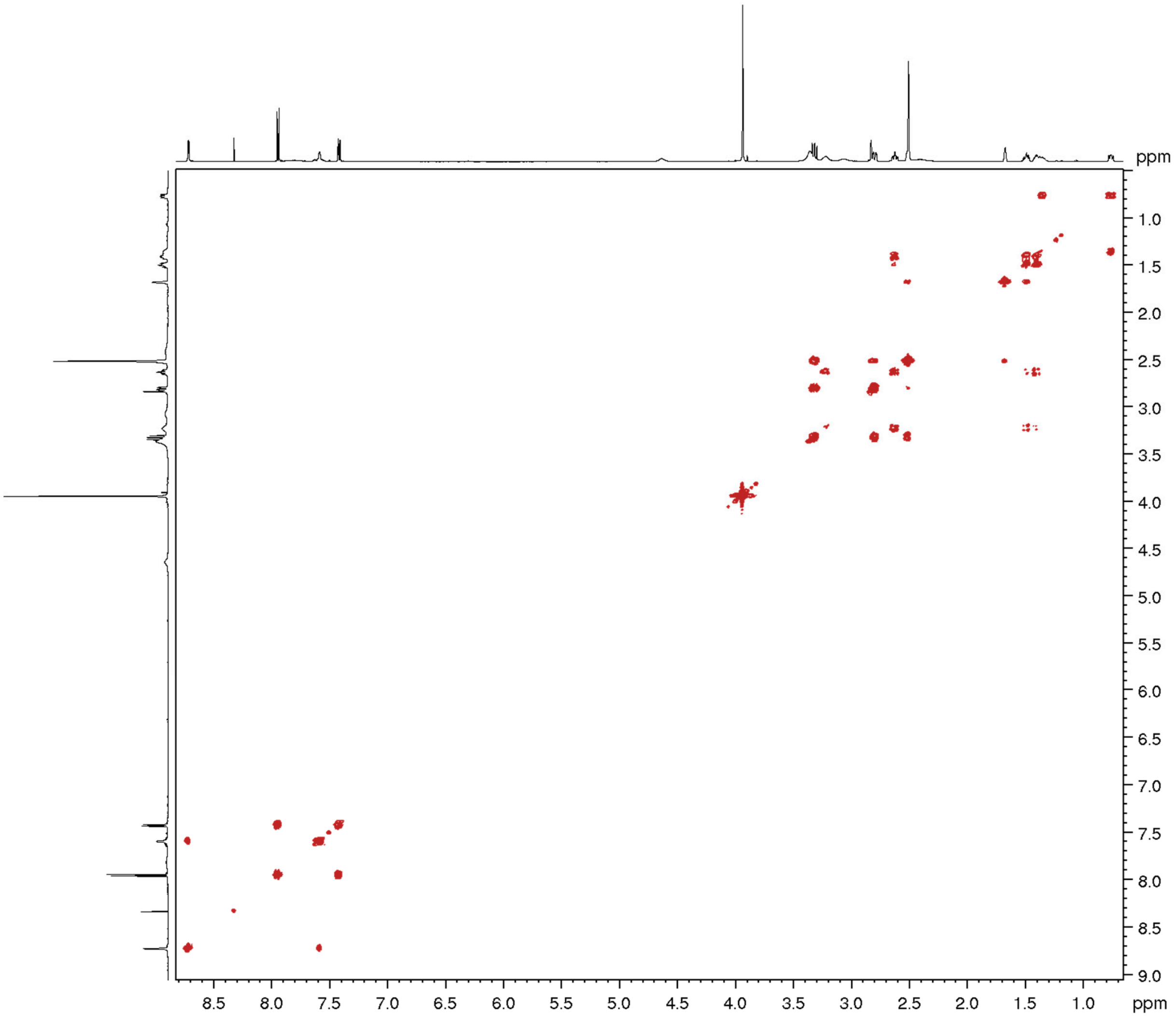

Figure S7. COSY NMR Spectrum of **DQ-N** (DMSO, 25 °C).

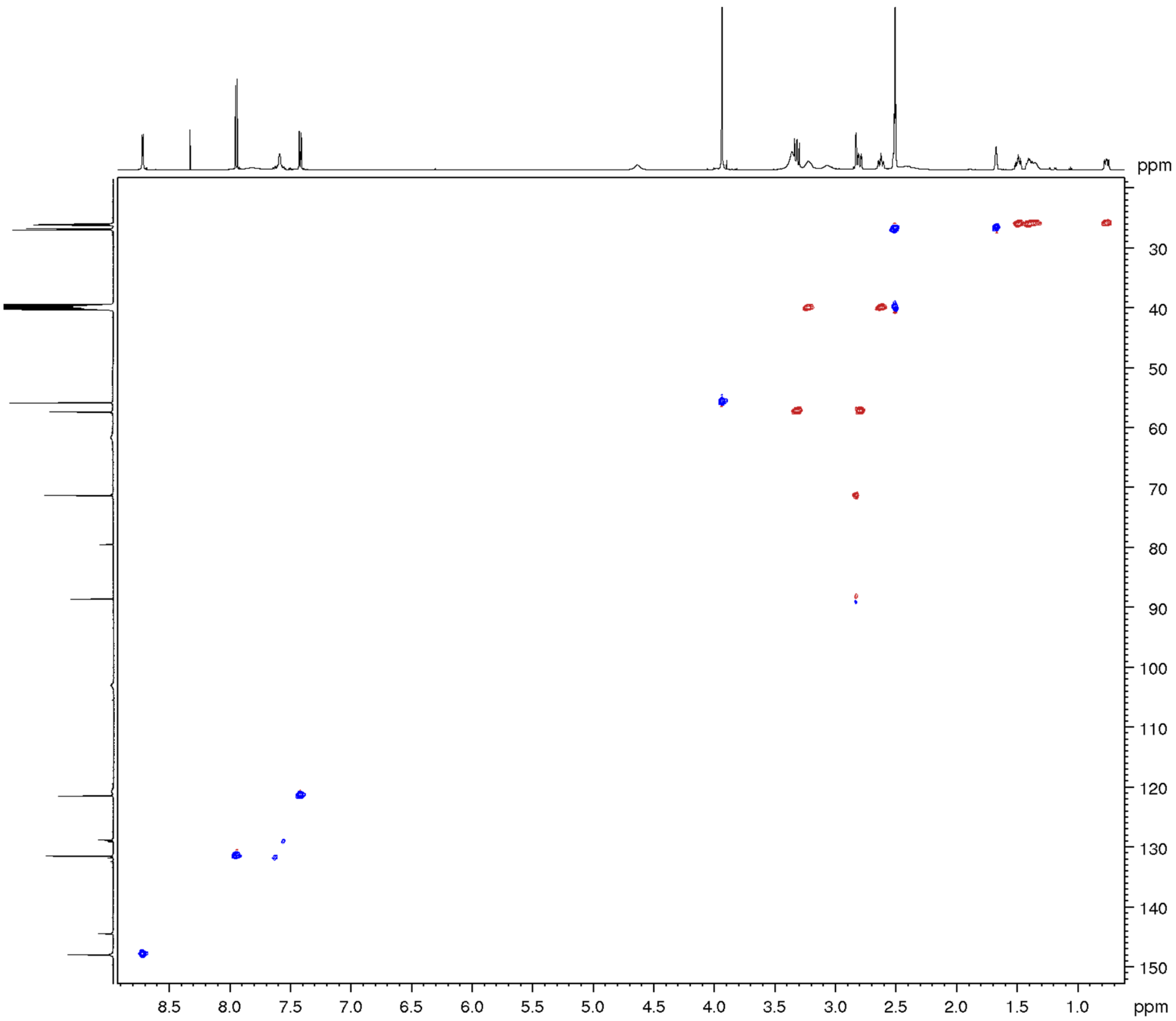

Figure S8. Edited HSQC NMR Spectrum of **DQ-N** (DMSO, 25 °C).

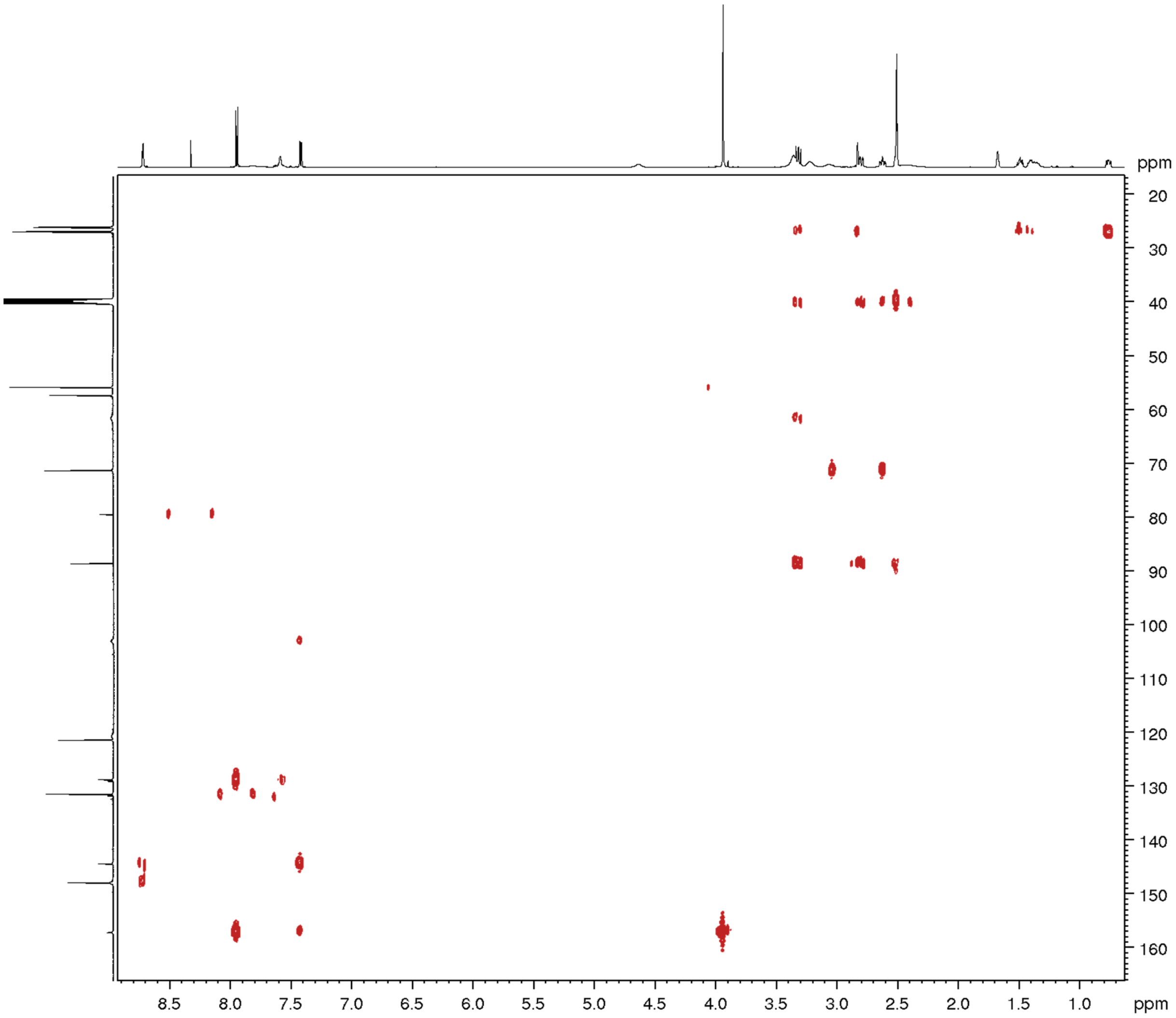

Figure S9. HMBC NMR Spectrum of **DQ-N** (DMSO, 25 °C).

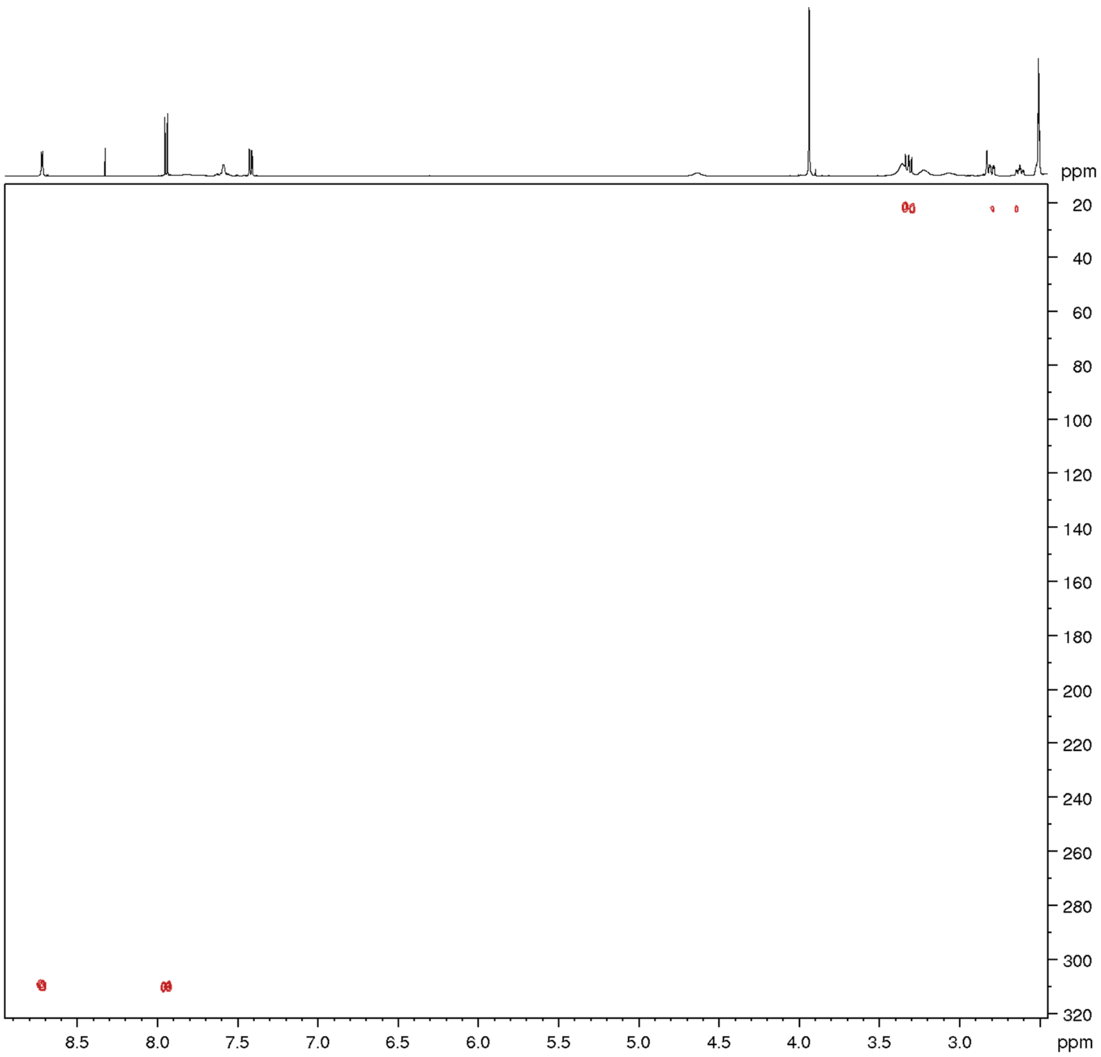

Figure S10. ^{15}N HMBC NMR Spectrum of **DQ-N** (DMSO, 25 °C).

3.2. NMR Spectra of DQ-SQ

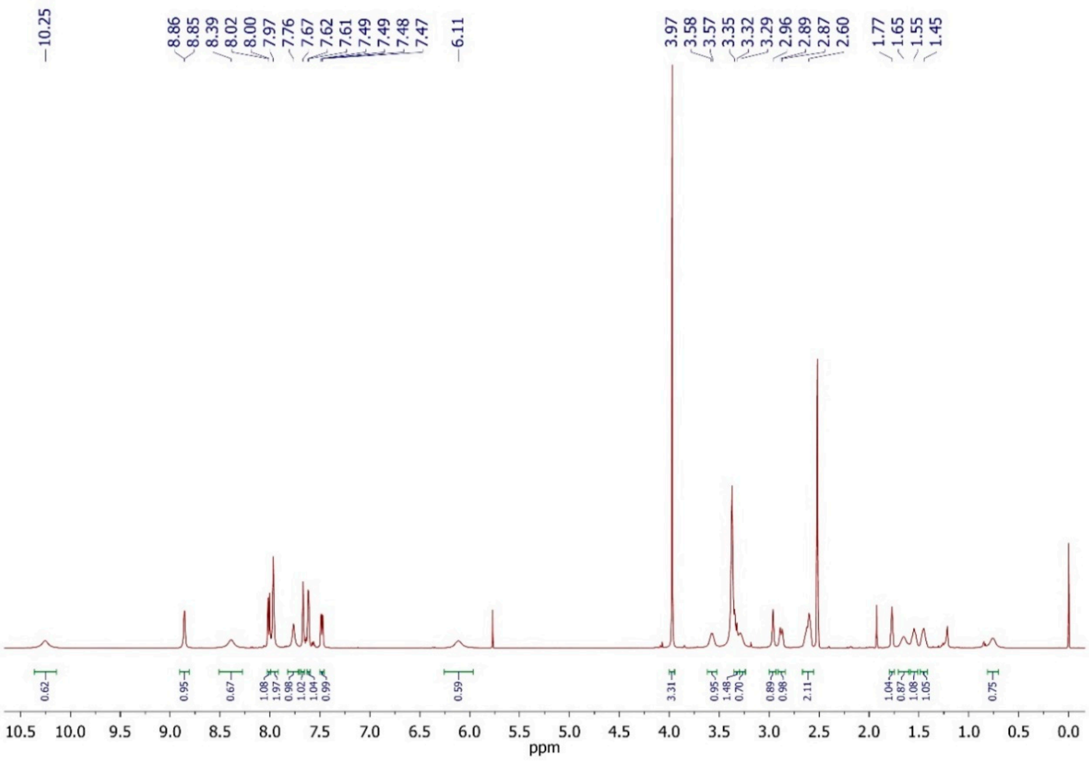

Figure S11a. NMR Spectrum of **DQ-SQ** (600 MHz, DMSO, 25 °C).

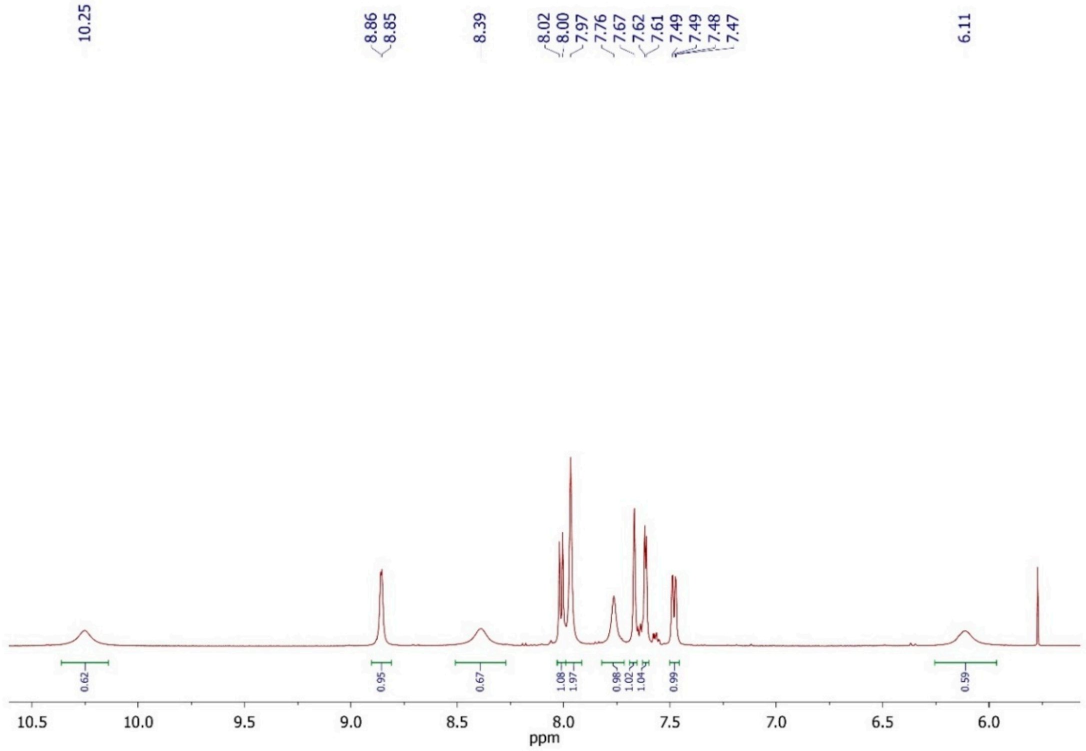

Figure S11b. NMR Spectrum of **DQ-SQ** in a range between 10.5 and 5.0 ppm (600 MHz, DMSO, 25 °C).

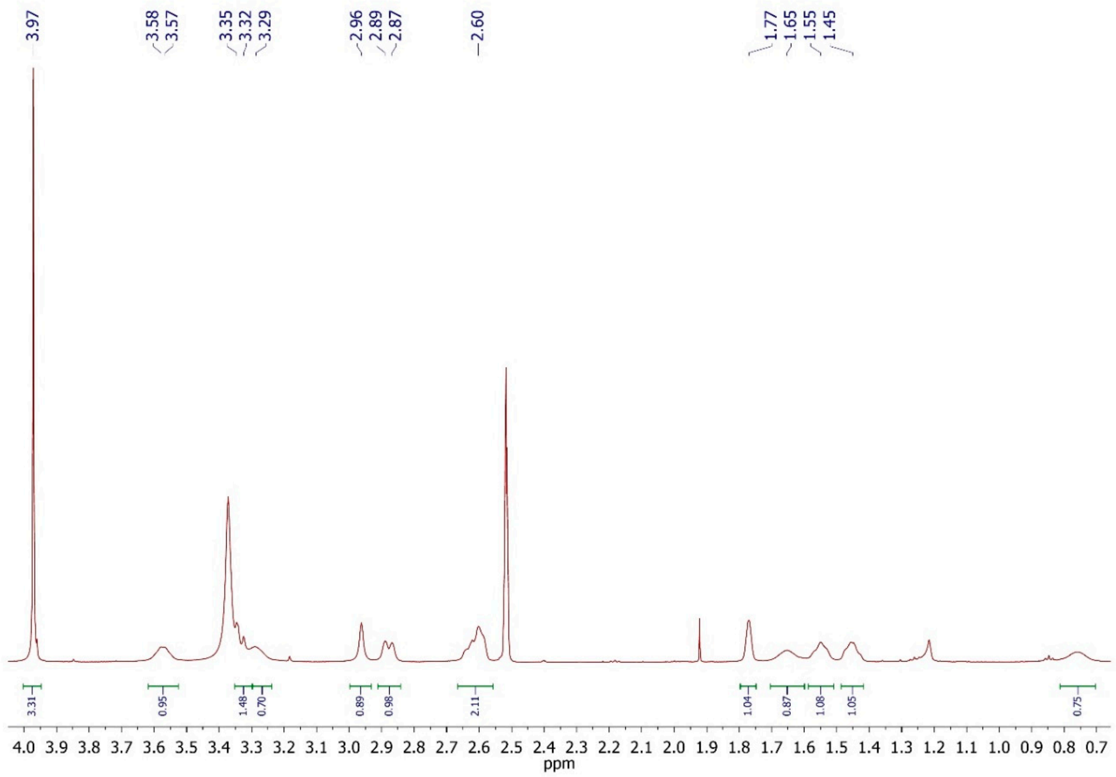

Figure S11c. NMR Spectrum of **DQ-SQ** in a range between 4.1 and 0.7 ppm (600 MHz, DMSO, 25°C).

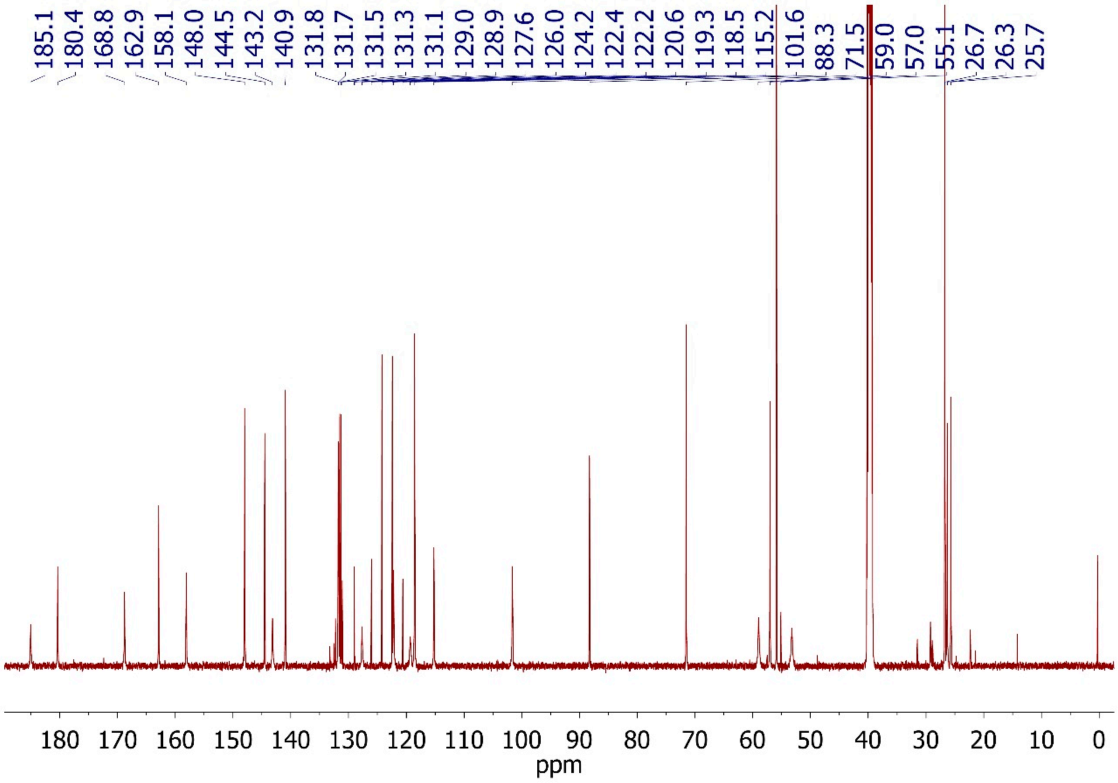

Figure S12a. ^{13}C NMR Spectrum of **DQ-SQ** (150 MHz, DMSO, 25 °C).

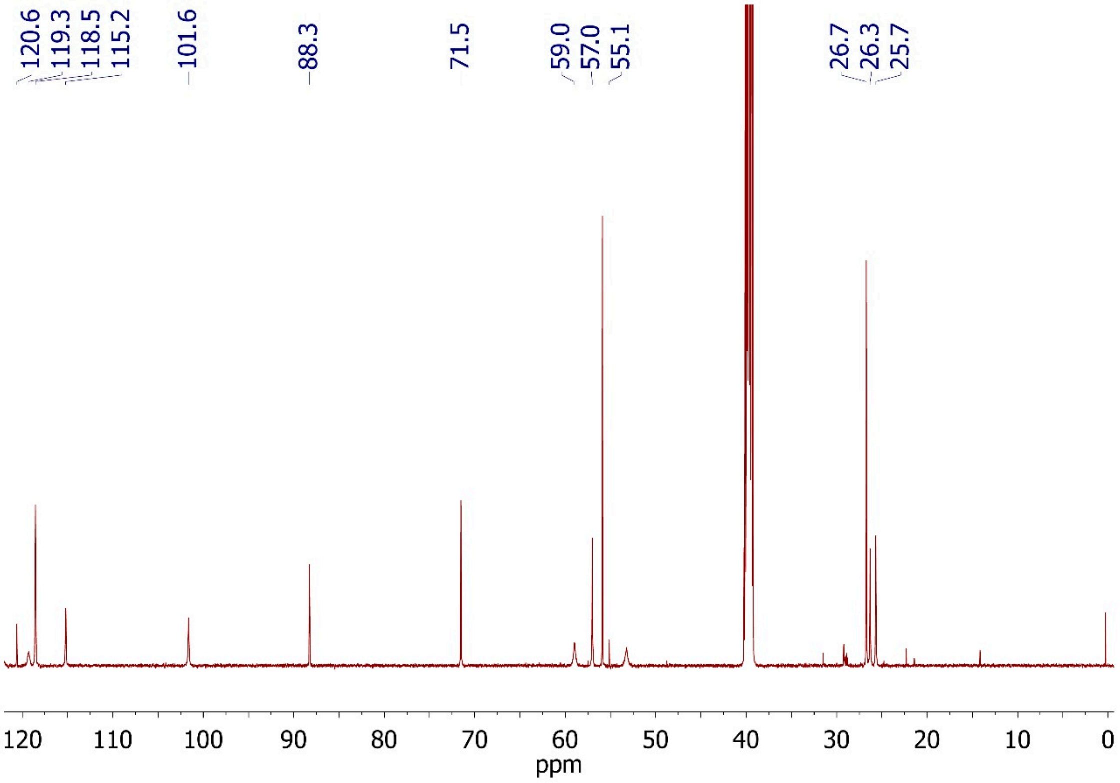

Figure S12b. ^{13}C NMR Spectrum of **DQ-SQ** in a range between 120.0 and 0.0 ppm (150 MHz, DMSO, 25°C).

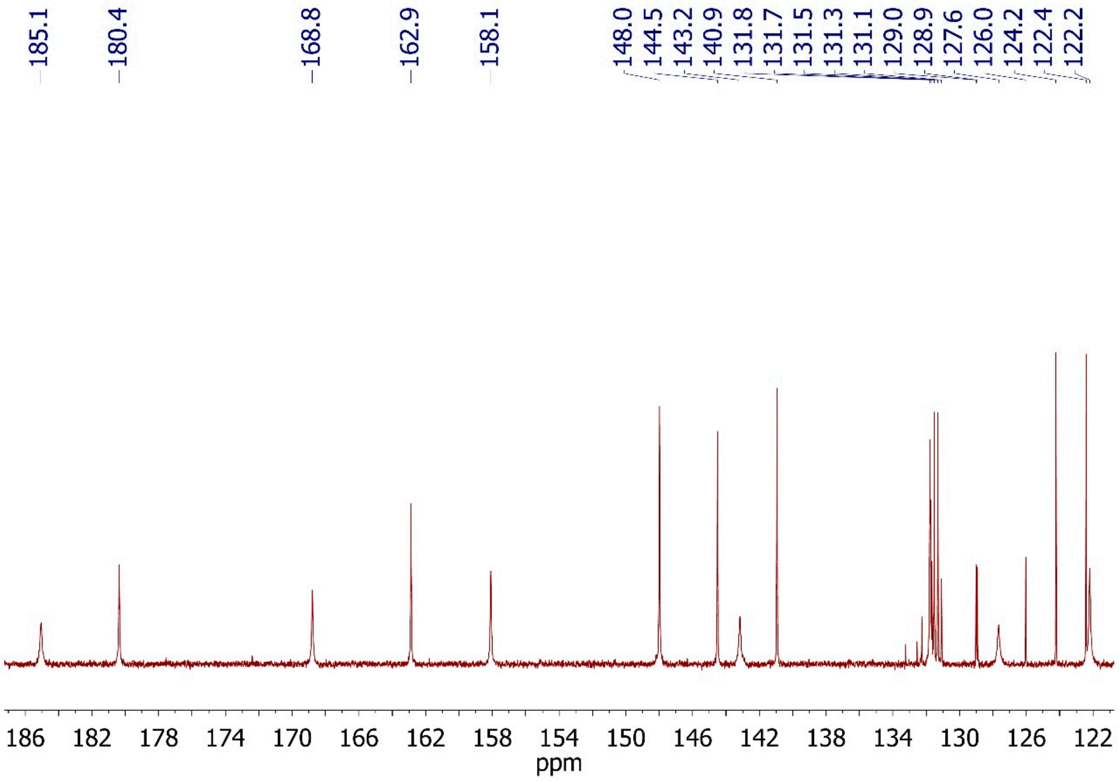

Figure S12c. ^{13}C NMR Spectrum of **DQ-SQ** in a range between 186.0 and 122.0 ppm (150 MHz, DMSO, 25 °C).

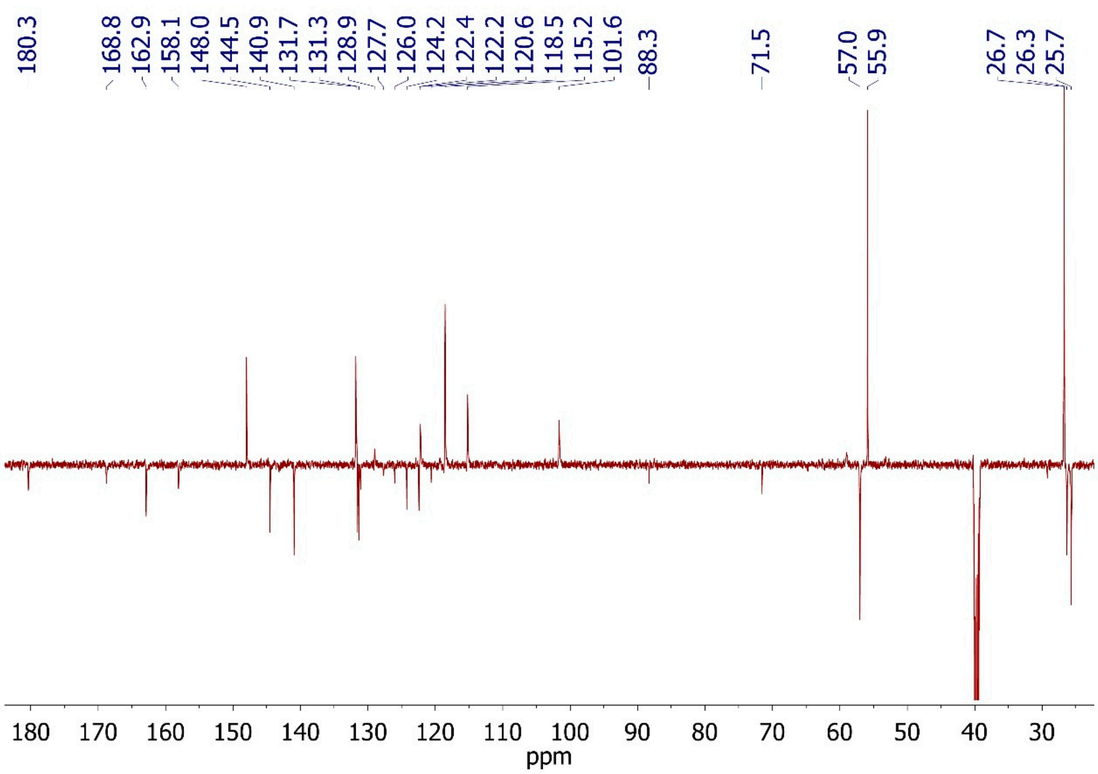

Figure S13. ^{13}C DEPT NMR Spectrum of **DQ-SQ** (150 MHz, DMSO, 25 °C).

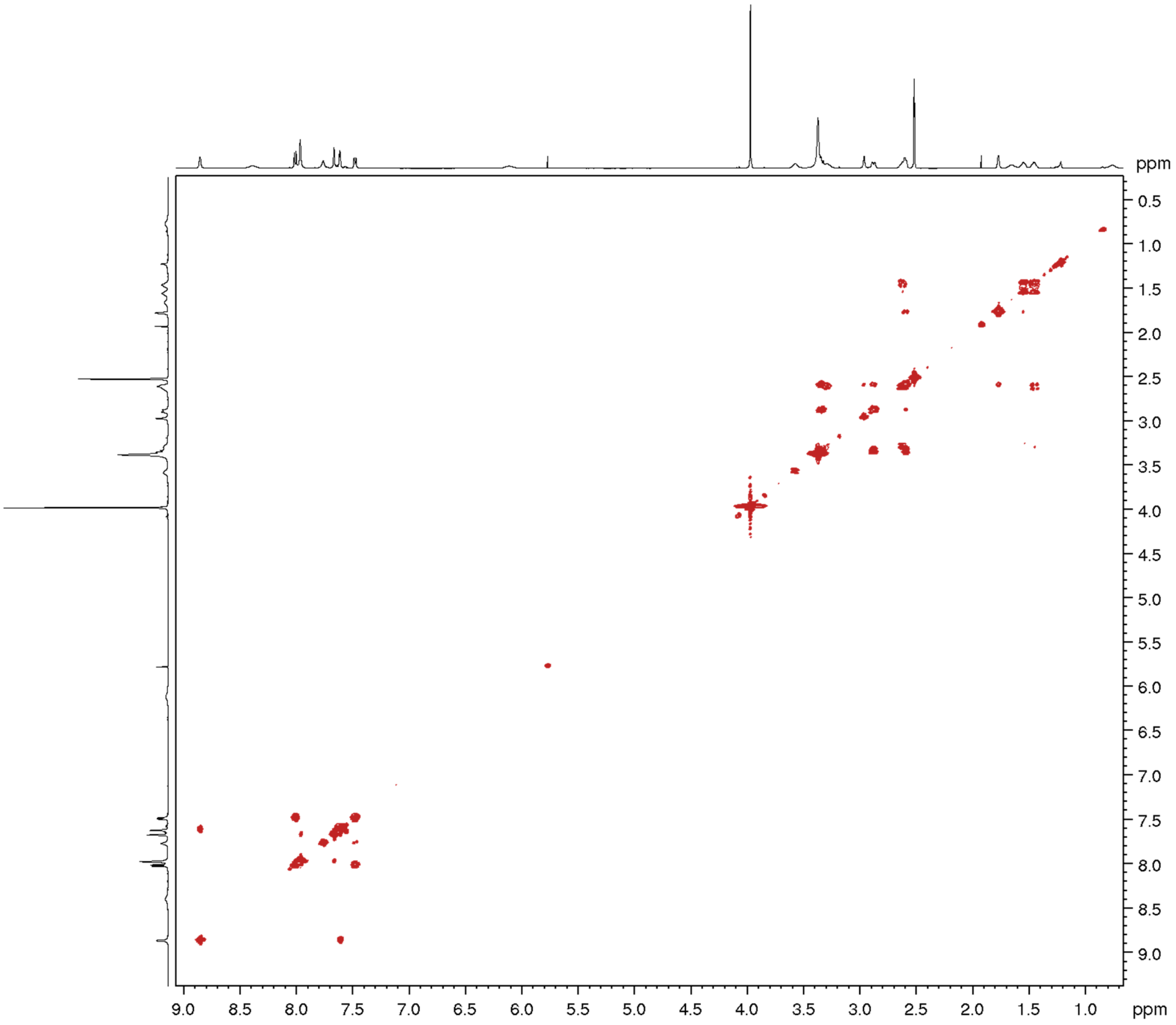

Figure S14. COSY NMR Spectrum of **DQ-SQ** (DMSO, 25 °C).

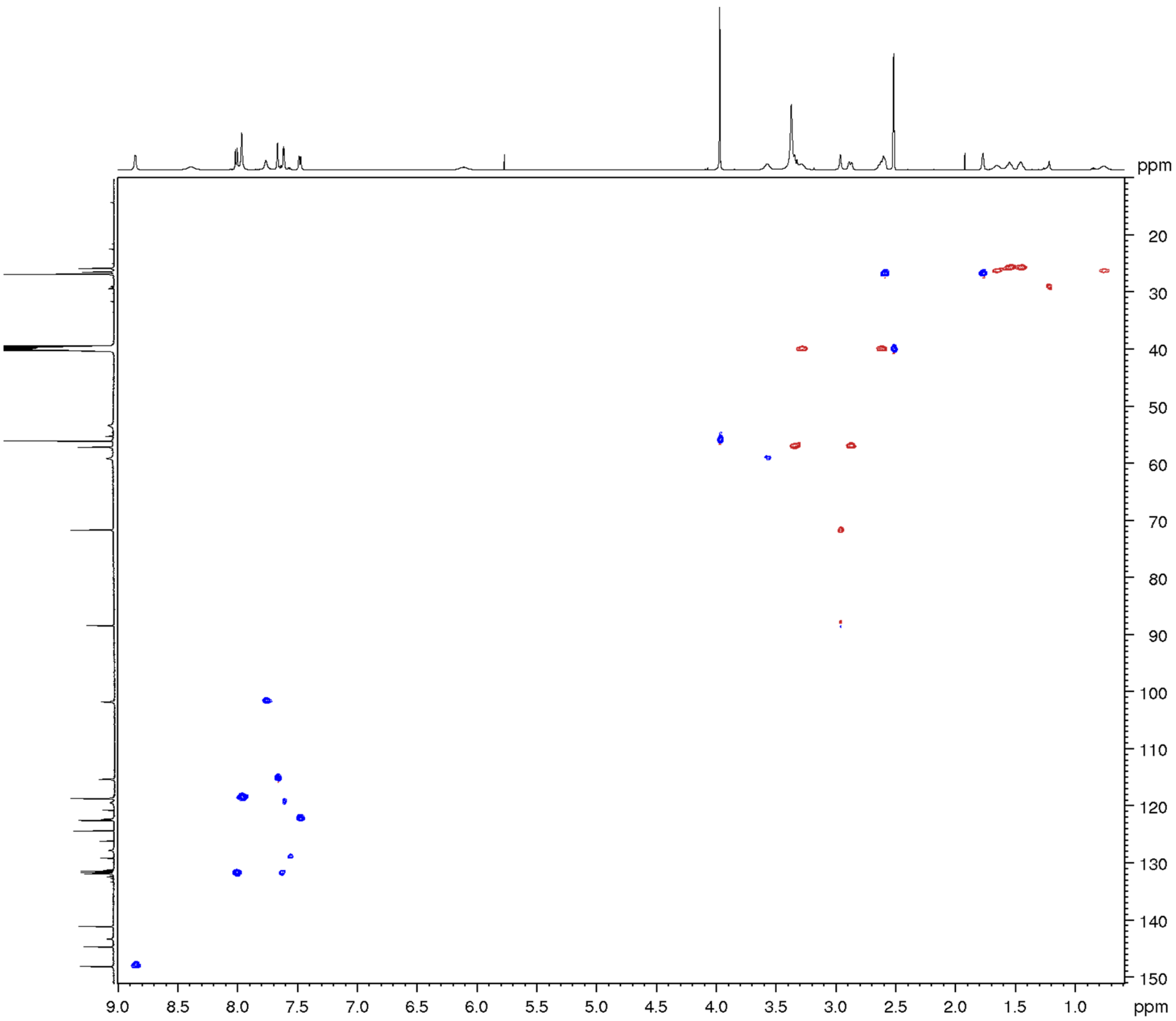

Figure S15. Edited HSQC NMR Spectrum of **DQ-SQ** (DMSO, 25 °C).

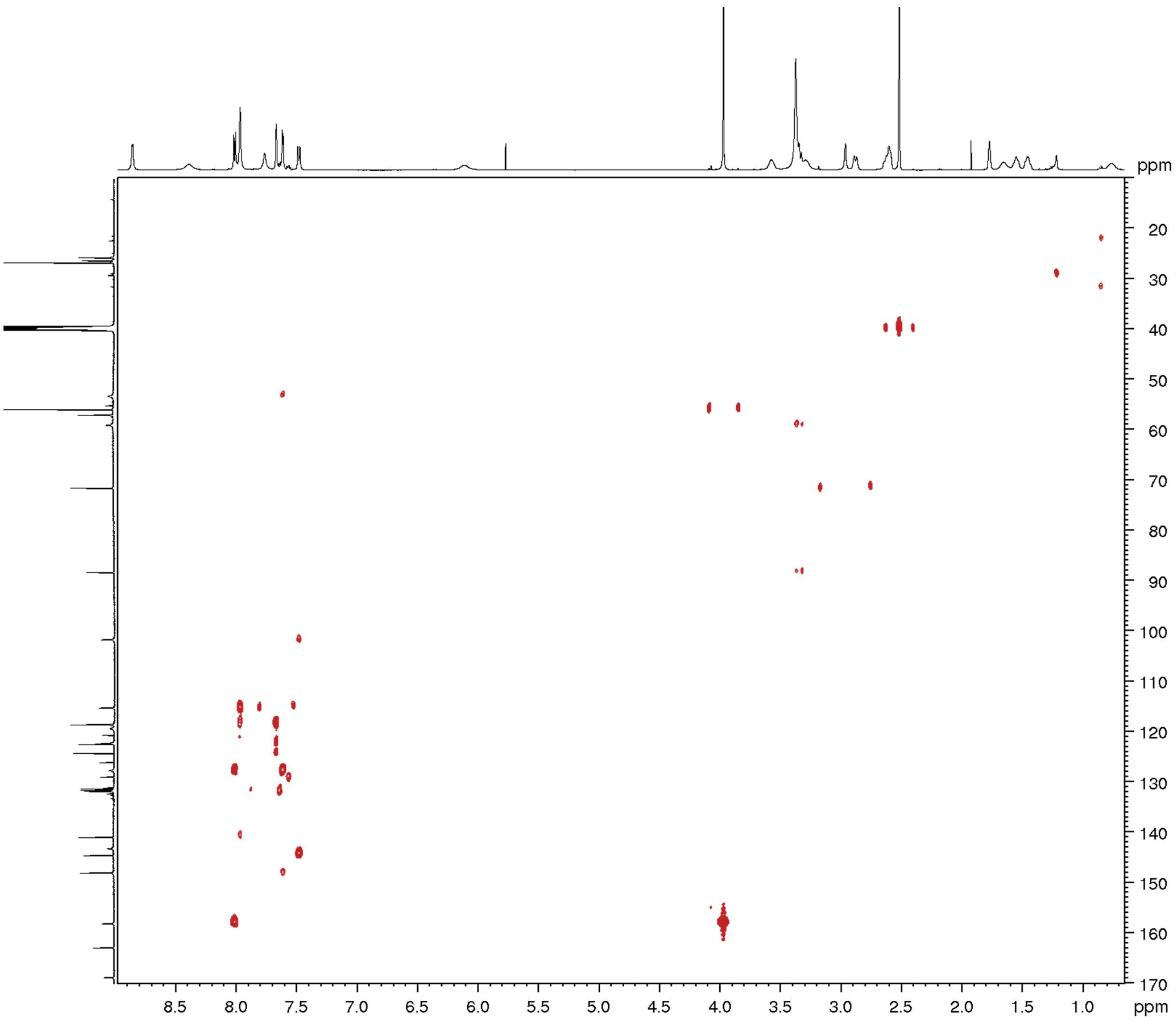

Figure S16. HMBC NMR Spectrum of **DQ-SQ** (DMSO, 25 °C).

4. Chiral HPLC Profiles of Michael Adducts 3

HPLC: Phenomonex Lux Cellulose–3 column (3 µm, 250 × 4.6 mm), eluent CH_3CN/20 mM NH_4OAc in H_2O = 40/60, isocratic mode; 0.6 mL·min^{-1}; UV detector 222 nm, 5 µL or 10 µL injection, 20 °C. Retention time for (*S*)–**3**: 11.94 min, for (*R*)–**3**: 14.20 min.

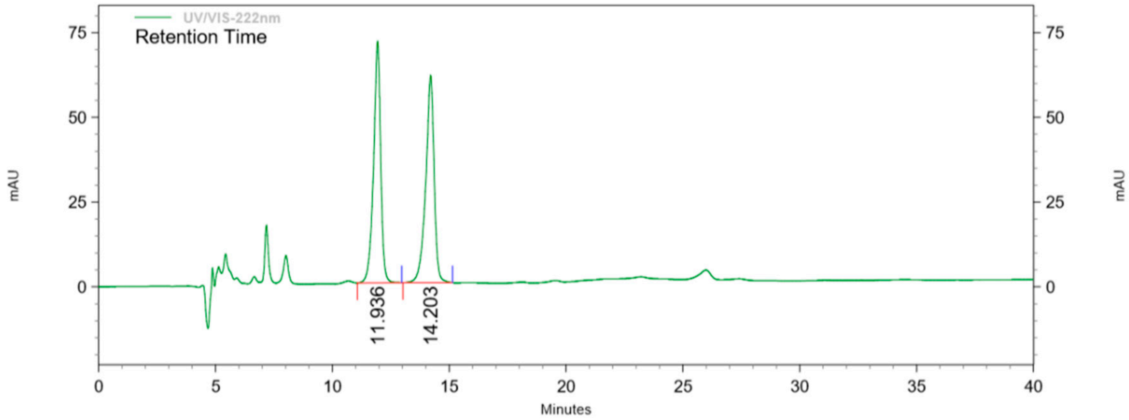

Name	Retention Time	Area	Area Percent
	11.936	1512904	49.812
	14.203	1524328	50.188

Totals		3037232	100.000

Figure S17. HPLC Chromatogram of racemic **3.**

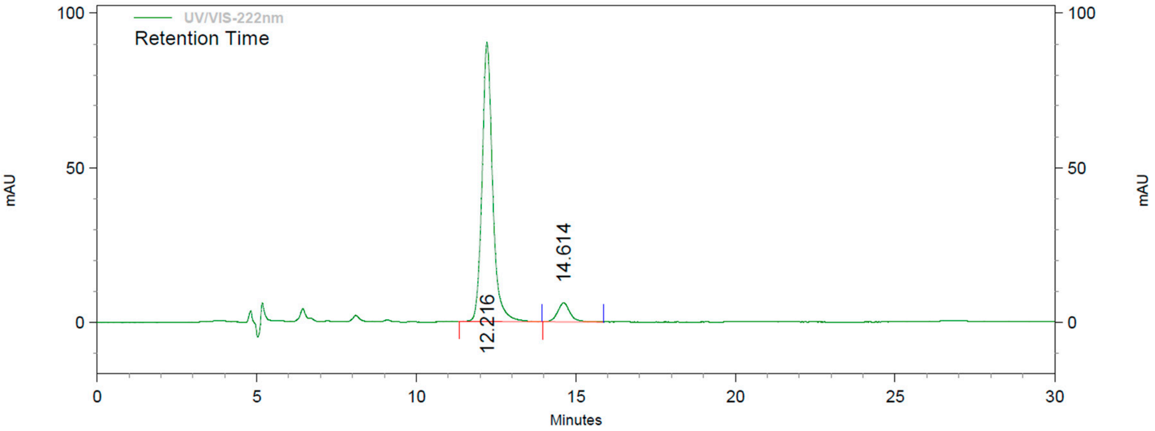

Name	Retention Time	Area	Area Percent
	12.216	2079914	92.707
	14.614	163616	7.293

Totals		2243530	100.000

Figure S18. HPLC Chromatogram of enantiomeric enriched **3** (Table 3, Entry 1).

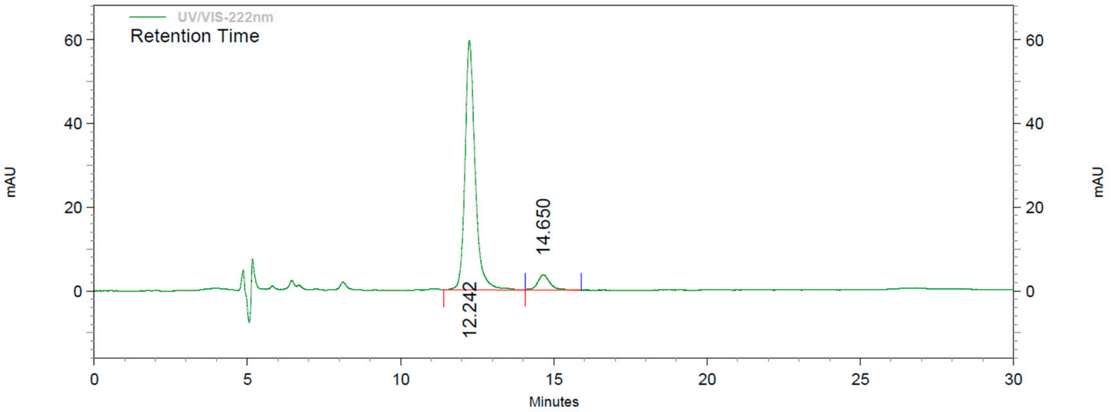

Name	Retention Time	Area	Area Percent
	12.242	1362764	93.201
	14.650	99411	6.799
Totals		1462175	100.000

Figure S19. HPLC Chromatogram of enantiomeric enriched **3** (Table 3, Entry 26).

5. HRMS Spectra of New Compounds

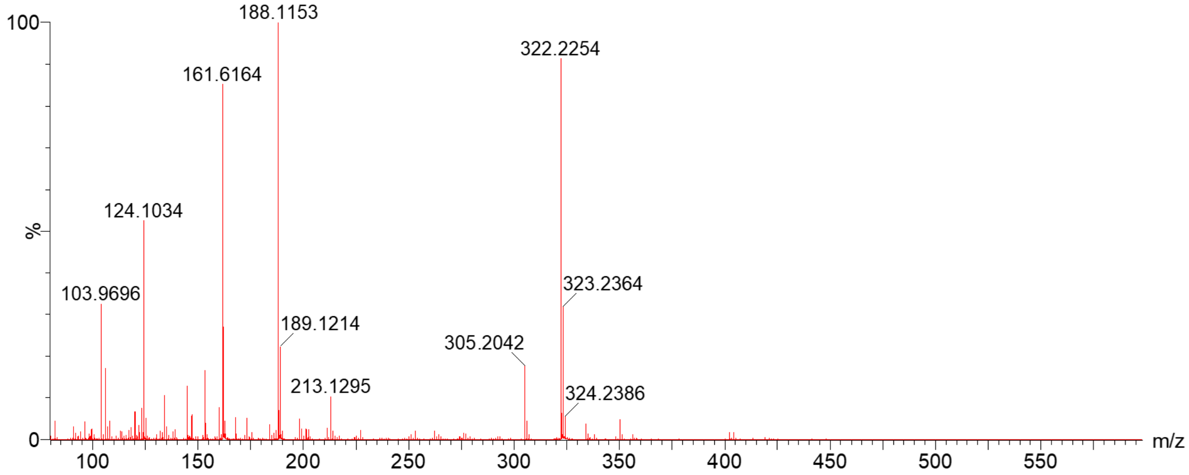

Figure S20. HRMS Spectrum of **DQ–N.**

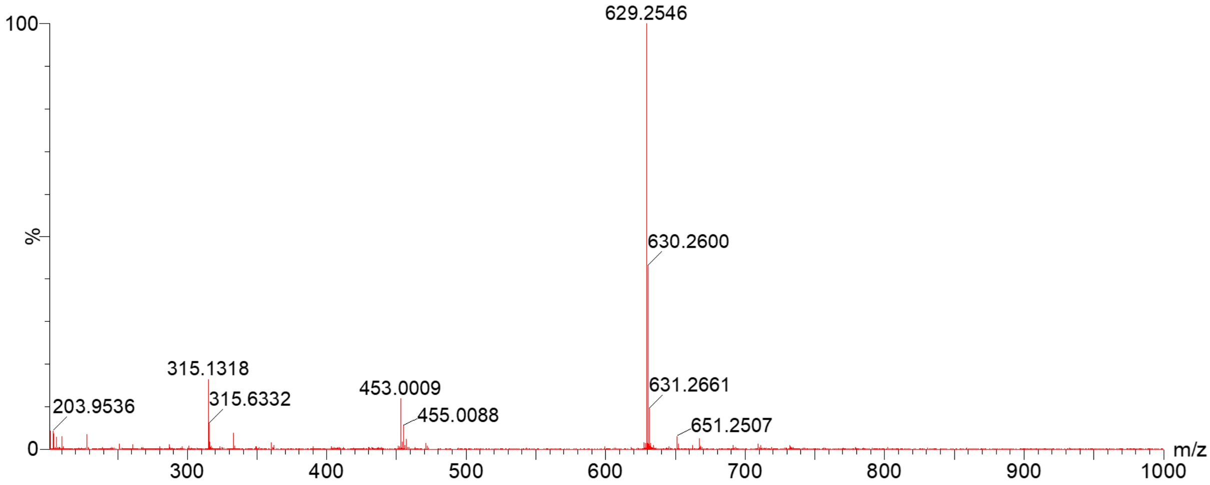

Figure S21. HRMS Spectrum of **DQ–SQ.**

6. Quantum Chemical Computations

Table S6. Relative free−energies (G_{rel}) in THF solvent and relative energies (E_{rel}) in the gas phase (both in kJ·mol^{-1}) compared to the lowest energy tautomers using two different solvent models (SMx and SS(V)PE) on three different level of theories (ωB97X−D/6−31G* and ωB97X−D/6−31+G**// ωB97X−D/6−31G* using SM8 and SS(V)PE and ωB97X−D/6−311++G**// ωB97X−D/6−31G* using SM12 solvent model).

	G_{rel} SM8		G_{rel} SM12	G_{rel} SS(V)PE		E_{rel} gas
	**6−31G***	**6−31+G****	**6−311++G****	**6−31G***	**6−31+G****	**6−31+G****
**Neutral**						
**HQ-TU**_i	0	0	0	0	0	0
**HQ-TU**_ii	28	35	32	42	34	79
**HQ-TU**_iii	60	64	59	61	56	118
**HQ-TU**_iv	74	65	64	69	67	50
**HQ-TU**_v	88	76	79	85	85	68
**HQ-TU**_vi	88	79	87	100	90	161
**HQ-TU**_vii	110	90	94	101	90	96
**HQ-TU**_viii	125	133	129	134	132	204
**HQ-TU**_ix	137	136	146	122	130	129
**HQ-TU**_x	188	194	187	181	191	178
**Q-TU**_i	0	0	0	0	0	0
**Q-TU**_ii	27	34	32	41	34	78
**Q-TU**_iii	64	66	63	66	61	123
**Q-TU**_iv	75	70	65	70	67	52
**DQ-TU**_i	0	0	0	0	0	0
**DQ-TU**_ii	32	42	40	49	42	86
**DQ-TU**_iii	68	66	66	69	67	120
**DQ-TU**_iv	74	69	70	72	68	53
**Deprotonated anion**						
**HQ-TU**_anion_1	0	0	0	0	0	0
**HQ-TU**_anion_2	12	16	9	4	3	26
**Q-TU**_anion_1	0	0	0	0	0	0
**Q-TU**_anion_2	14	18	10	7	9	28
**DQ-TU**_anion_1	0	0	0	0	0	0
**DQ-TU**_anion_2	13	19	12	7	5	29
**Protonated cation**						
**HQ-TU**_cation_1	0	0	0	0	0	0
**HQ-TU**_cation_2	78	65	62	67	58	50
**Q-TU**_cation_1	0	0	0	0	0	0

**Q-TU**_cation_2	83	71	69	69	59	51
**DQ-TU**_cation_1	0	0	0	0	0	0
**DQ-TU**_cation_2	71	66	64	77	69	50

Table S7. Protonation and deprotonation free energies (ΔG) in THF solvent and energies in the gas phase (ΔE) (in kJ·mol^{-1}) using two different solvent models (SMx and SS(V)PE) on three different level of theories (ωB97X–D/6–31G* and ωB97X–D/6–31+G**// ωB97X–D/6–31G* using SM8 and SS(V)PE and ωB97X–D/6–311++G**// ωB97X–D/6–31G* using SM12 solvent model).

	**ΔG SM8**		**ΔG SM12**	**ΔG SS(V)PE**		**ΔE gas**
	**6–31G***	**6–31+G****	**6–311++G****	**6–31G***	**6–31+G****	**6–31+G****
**Deprotonated anion**						
**HQ-TU**_anion_1	156	132	150	195	178	1406
**HQ-TU**_anion_2	168	148	159	199	181	1432
**Q-TU**_anion_1	155	128	148	192	177	1402
**Q-TU**_anion_2	168	146	157	198	181	1430
**DQ-TU**_anion_1	154	125	144	189	172	1398
**DQ-TU**_anion_2	167	145	157	197	178	1427
**Protonated cation**						
**HQ-TU**_cation_1	−74	−41	−44	−38	−32	−1029
**HQ-TU**_cation_2	−24	−11	−14	−13	−8	−1057
**Q-TU**_cation_1	−77	−42	−46	−37	−29	−1023
**Q-TU**_cation_2	−20	−5	−9	−9	−4	−1050
**DQ-TU**_cation_1	−59	−36	−40	−36	−29	−961
**DQ-TU**_cation_2	−21	−6	−9	−8	−2	−950
**THF**						
**Monomer THF-H**	−1078	−1079	−1078	−1059	−1059	
**Dimer THF-H**	−1162	−1163	−1158	−1131	−1131	
**Dimer H-THF**	−1162	−1163	−1158	−1130	−1130	
**Dimer THF-2H**	−1069	−1072	−1067	−1028	−1030	

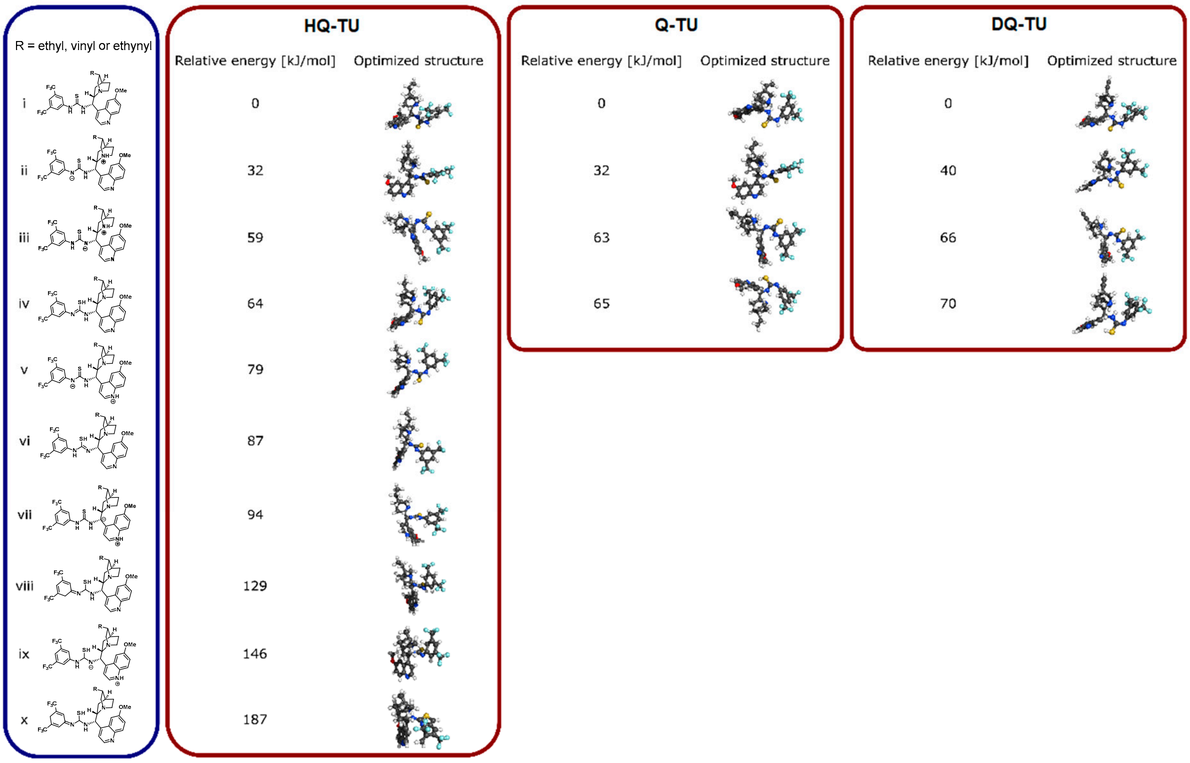

Figure S22. Geometric structures of the studied tautomers and their relative Gibbs free energies in THF, computed using the ωB97X−D/6−311++G**+SM12 method (for higher resolution see below: **Figure S22a–c**).

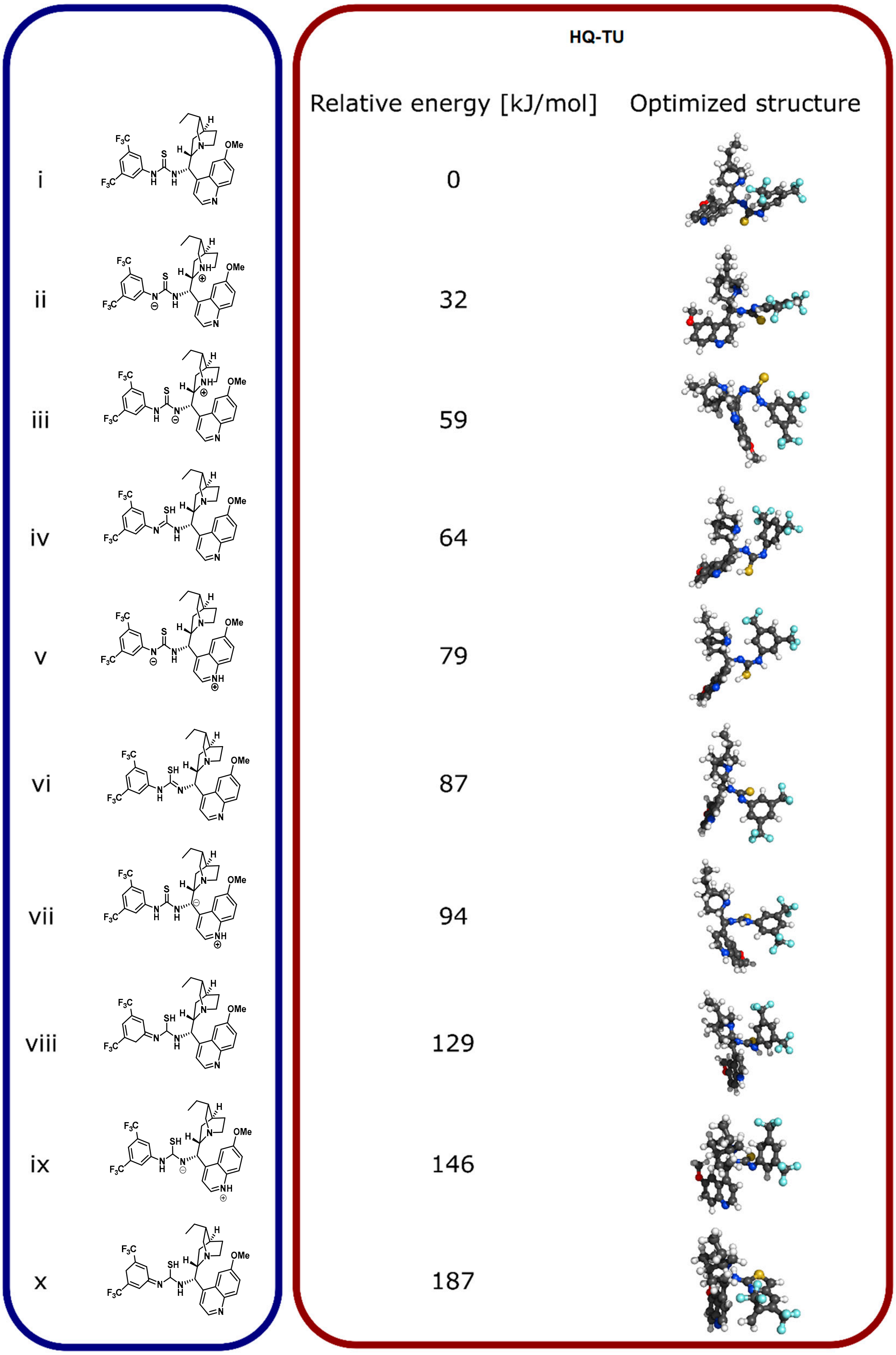

Figure S22a. Geometric structures of the studied tautomers of **HQ-TU** and their relative Gibbs free energies in THF, computed using the ωB97X–D/6–311++G**+SM12(THF)//ωB97X–D/6–31G*+SM8(THF) method.

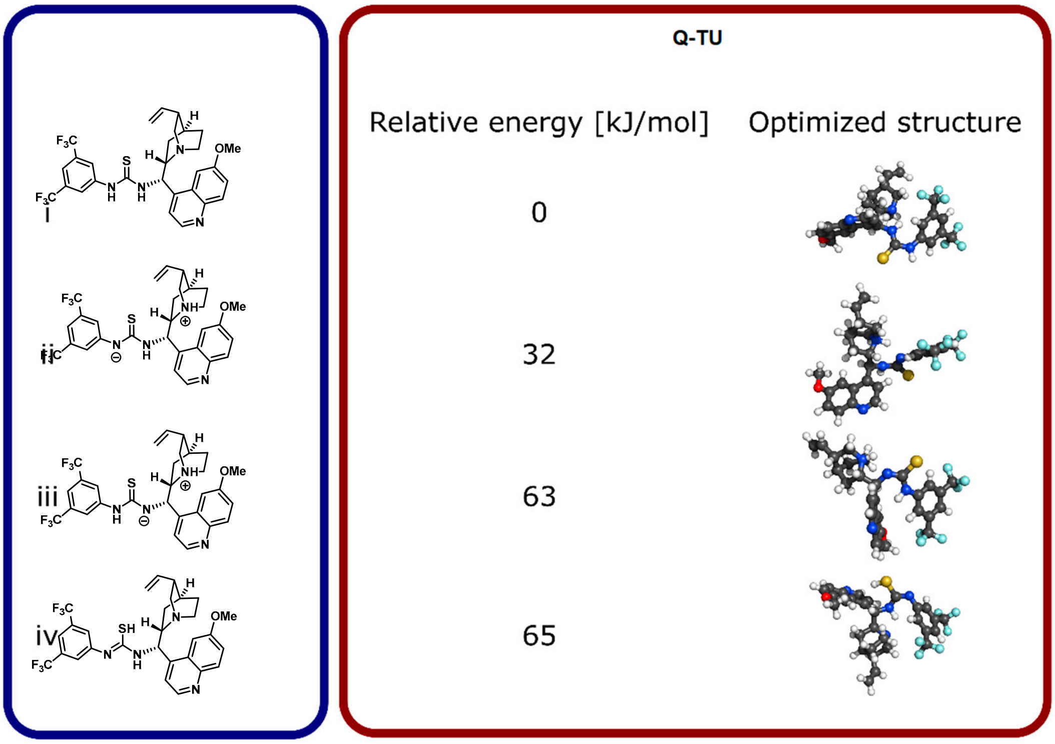

Figure s22b. Geometric structures of the studied tautomers of **Q-TU** and their relative Gibbs free energies in THF, computed using the ωB97X−D/6−311++G**+SM12(THF)//ωB97X−D/6−31G*+SM8(THF) method.

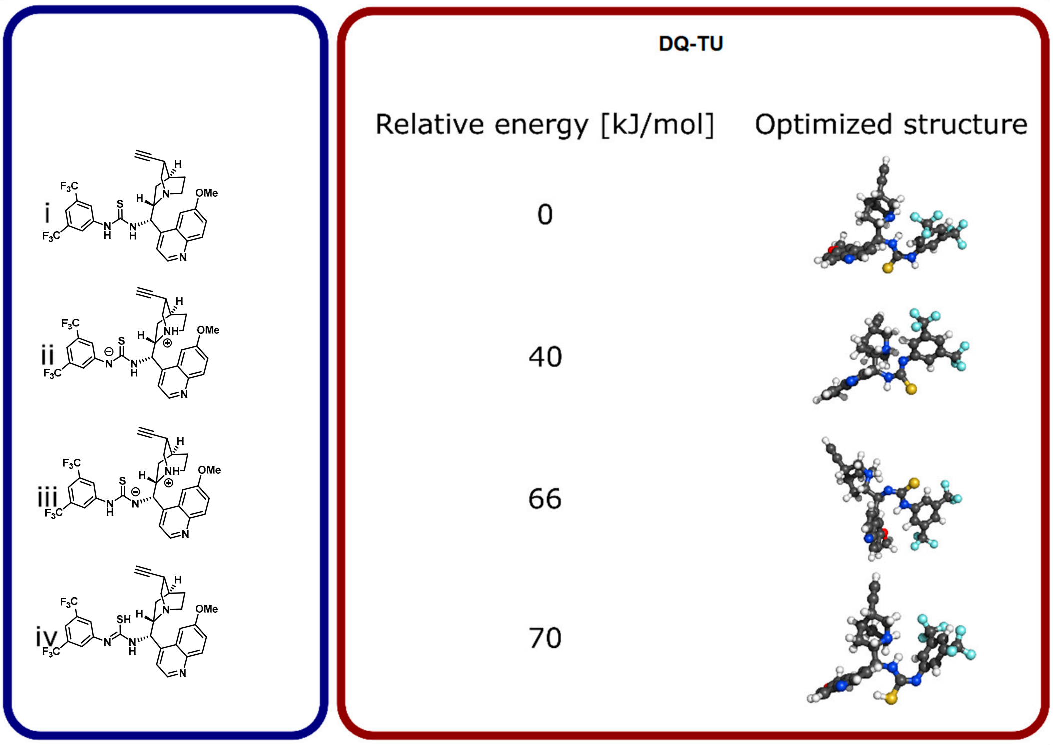

Figure S22c. Geometric structures of the studied tautomers of **DQ-TU** and their relative Gibbs free energies in THF, computed using the ωB97X−D/6−311++G**+SM12(THF)//ωB97X−D/6−31G*+SM8(THF) method.

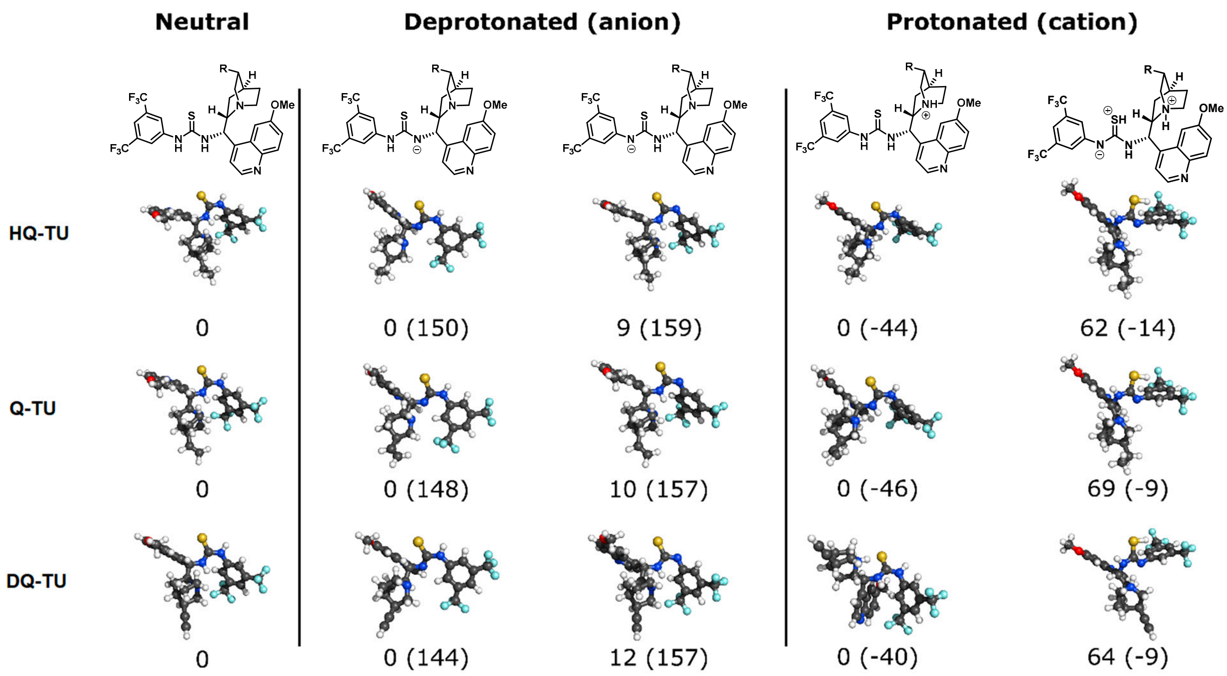

Figure s23. Geometric structures of the neutral, deprotonated and protonated form of *i* and their relative Gibbs free energies in THF. Protonation and deprotonation Gibbs free energies of the corresponding neutral tautomer are indicated in parenthesis. (ωB97X−D/6−311++G**+SM12(THF)//ωB97X−D/6−31G*+SM8(THF) method).

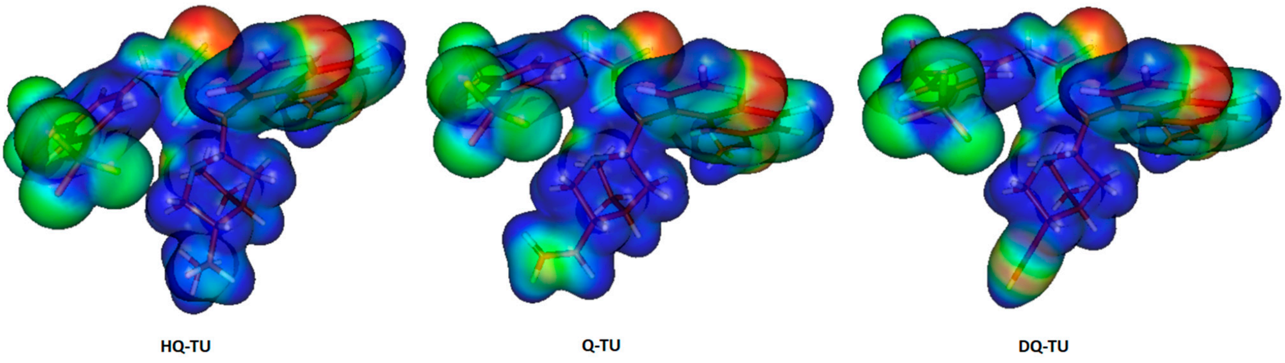

Figure s24. Electrostatic potentials (ESP) mapped onto the 0.01 a.u. electron density isosurface of **HQ-TU**, **Q-TU** and **DQ-TU**. Colors: ESP<−0.05a.u. – red, ESP=−0.02a.u. – yellow, ESP=0.0a.u. – green, ESP=0.02a.u. – light blue, ESP>0.05a.u. blue.

Table S8a. Total energies in Hartree and optimized coordinates of the different neutral, protonated and deprotonated **HQ-TU**, **Q-TU** and **DQ-TU** tautomers. (ωB97X−D/6−31G* +SM8(THF) level of theory).

HQ-TU_i				
E = −2414.09342337501				
1	C	−8.7444564215	3.1770499741	−2.7908482866
2	O	−9.2837799595	2.3728726280	−1.7546787932
3	C	−9.0796747534	1.2692310323	0.2960376298
4	C	−7.1618716773	2.4545667292	−0.5948919635
5	C	−6.3933723983	2.0757456347	0.5401771850
6	C	−8.3591627578	0.8836464631	1.3868054472
7	C	−6.9994136245	1.2690466449	1.5431715495

8	C	−5.0326876157	2.4549357953	0.7413317708
9	C	−4.4022855729	2.0059696556	1.8769897698
10	C	−5.0920777244	1.2006355988	2.8003481001
11	N	−6.3485242623	0.8374733486	2.6588865347
12	C	−4.2978863978	3.4035368124	−0.1900331641
13	C	−4.2645557980	4.8197653246	0.4448364776
14	C	−5.6360188728	5.5367036797	0.4878915909
15	C	−5.3740569479	7.0274759556	0.2001747762
16	C	−4.2383561448	7.4821518018	1.1489204412
17	C	−2.9389792770	6.7941565013	0.6541757471
18	C	−4.0847474527	8.9903628153	1.3985665593
19	C	−3.8887693695	9.9037804877	0.1887537871
20	N	−3.2501608509	5.6556592599	−0.2205555501
21	N	−2.9044866749	3.0515207725	−0.3861964882
22	C	−2.4373220030	2.0296908034	−1.1192347440
23	N	−1.0793728948	2.0141383577	−1.2934263989
24	C	−0.1460769185	2.9616669989	−0.8197189804
25	C	−0.1437984935	3.3802484746	0.5132054814
26	C	0.7661931161	4.3401340550	0.9353805586
27	C	1.7163241439	4.8593193004	0.0631136841
28	C	0.8050822766	3.4759571617	−1.6997877522
29	C	1.7354119767	4.4072828063	−1.2498478773
30	C	0.7431276875	4.7682179767	2.3782509739
31	C	2.7560284441	4.9278105558	−2.2262349129
32	F	1.4039727697	5.9196886323	2.5745210328
33	F	1.3067311652	3.8429329996	3.1762235657
34	F	−0.5147293542	4.9449686823	2.8223846884
35	F	3.5937643366	3.9526624625	−2.6227410437
36	F	3.5053986728	5.9110210300	−1.7027689567
37	F	2.1731755878	5.4129060650	−3.3358983313
38	S	−3.4036043985	0.8185394520	−1.8026838659
39	H	−8.4282101374	4.1559896596	−2.4113259272
40	H	−9.5500314046	3.3172443953	−3.5119771598
41	H	−7.9013214005	2.6805198022	−3.2856225463
42	C	−8.4737087763	2.0626308880	−0.7146413831
43	H	−10.1174079505	0.9790063126	0.1707189995
44	H	−8.7985888250	0.2724650105	2.1681464520
45	H	−6.6996532623	3.0377692470	−1.3785028736
46	H	−4.5837893912	0.8476021060	3.6951578694
47	H	−3.3660473985	2.2663217289	2.0678144228
48	H	−4.7950981170	9.9744159866	−0.4209058362
49	H	−3.0698277308	9.5727816794	−0.4603890282
50	H	−3.6453119424	10.9190887468	0.5193064266
51	H	−3.2326452080	9.1225541808	2.0794430850
52	H	−4.9648655928	9.3372703857	1.9550210199
53	C	−4.9602986112	7.1202088962	−1.2797591853
54	C	−3.7607729445	6.1630393691	−1.5017853796
55	H	−4.4784591589	7.0568228903	2.1336118123
56	H	−2.3139132527	7.4865048395	0.0784380554
57	H	−2.3328797769	6.4391067240	1.4937846554
58	H	−6.2808120284	7.6146742021	0.3818102421
59	H	−4.6959219601	8.1420660838	−1.5575318543

60	H	−5.8081291174	6.8322313241	−1.9114014138
61	H	−2.9355063849	6.6668535831	−2.0137016995
62	H	−4.0374162451	5.3067538464	−2.1250530478
63	H	−6.3228657701	5.1289417732	−0.2627395776
64	H	−6.1112251504	5.3912483851	1.4627866342
65	H	−3.8981749310	4.6803109897	1.4694162548
66	H	−4.7967999523	3.4319599939	−1.1637764115
67	H	−2.3366347074	3.9048100874	−0.3346648979
68	H	−0.7430711081	1.3422100417	−1.9725613763
69	H	−0.8582172711	2.9627255351	1.2124910416
70	H	0.8026170389	3.1590553994	−2.7379984107
71	H	2.4251969082	5.6048731768	0.3990707954
		**HQ-TU_ii**		
		E = −2414.08284281588		
1	C	−6.6869281877	1.6305948211	1.8965226655
2	O	−6.8091197761	2.4543796116	0.7474237074
3	C	−5.9259955170	3.3810039171	−1.2082245915
4	C	−4.5108996635	1.9722696460	0.1666282123
5	C	−3.4345339960	2.1466111440	−0.7484645599
6	C	−4.9091999578	3.5690256308	−2.0955600739
7	C	−3.6391352613	2.9599694621	−1.8984865136
8	C	−2.1488723754	1.5438970169	−0.5942721708
9	C	−1.2024734170	1.7882736537	−1.5635603425
10	C	−1.5104574838	2.6152011723	−2.6583039573
11	N	−2.6799638094	3.1906476309	−2.8373597583
12	C	−1.7968186159	0.6426242938	0.5891769328
13	C	−1.6635065131	−0.8038239191	0.0704756755
14	C	−3.0331709622	−1.4596911257	−0.2208086023
15	C	−3.0227309098	−2.8881755698	0.3483695273
16	C	−1.7841804045	−3.5943354512	−0.2470583530
17	C	−0.5398053136	−2.9630467093	0.4042352968
18	C	−1.7541761847	−5.1309927401	−0.2145161445
19	C	−1.9259807722	−5.8223664157	1.1377942696
20	N	−0.9252400970	−1.6672496560	1.0501089340
21	N	−0.6935359117	1.1535145108	1.4159504488
22	C	0.6651606471	1.1835198679	1.1202759995
23	N	1.0761229174	0.1610111431	0.4012776649
24	C	2.3904535908	−0.1144190048	0.0626787773
25	C	2.6190429293	−0.6784871916	−1.1992608944
26	C	3.8880758178	−1.0925831617	−1.5894195834
27	C	4.9706873759	−0.9707909163	−0.7303812989
28	C	3.4917013325	0.0060569498	0.9289487623
29	C	4.7510340679	−0.4210689927	0.5305770926
30	C	4.0556675886	−1.6834676940	−2.9607211174
31	C	5.9180775519	−0.2468207869	1.4619583228
32	F	5.3145145962	−2.0954419995	−3.1928032034
33	F	3.7462274559	−0.7949927251	−3.9259197552
34	F	3.2507866175	−2.7437057775	−3.1487174019
35	F	6.5508911321	0.9269239895	1.2590953274
36	F	6.8412015749	−1.2123242712	1.2951216160
37	F	5.5480155078	−0.2705477601	2.7529333067
38	S	1.5583353483	2.5218350254	1.7623662894

39	H	−7.6600956575	1.6538669455	2.3869845418
40	H	−5.9289717305	2.0178429762	2.5873416410
41	H	−6.4496940339	0.5965137602	1.6198776580
42	C	−5.7279844244	2.5697867795	−0.0589550766
43	H	−6.8977187594	3.8402850230	−1.3550712744
44	H	−5.0355252472	4.1841479887	−2.9802108258
45	H	−4.3713148743	1.3687296390	1.0521357644
46	H	−0.7547190101	2.8060691880	−3.4173138633
47	H	−0.2111725766	1.3536369735	−1.4852447429
48	H	−1.7242718197	−6.8939538628	1.0398076484
49	H	−2.9461487800	−5.7250749880	1.5213723095
50	H	−1.2379373159	−5.4393537008	1.9001809777
51	H	−0.8008189443	−5.4499131336	−0.6558356932
52	H	−2.5323774507	−5.4965682047	−0.8951137147
53	C	−2.9570667099	−2.7674065773	1.8833170108
54	C	−1.7257761053	−1.9298201761	2.2820682000
55	H	−1.7644318755	−3.3307312405	−1.3113759464
56	H	−0.1166735390	−3.5861158966	1.1953125841
57	H	0.2508425155	−2.7418903616	−0.3146560673
58	H	−3.9352083217	−3.4149730341	0.0548393964
59	H	−2.9147800246	−3.7465972981	2.3595045601
60	H	−3.8674499418	−2.2793144437	2.2422504443
61	H	−1.0689739721	−2.4486367495	2.9812248342
62	H	−1.9739569890	−0.9623272627	2.7179743104
63	H	−3.8426864742	−0.8809076947	0.2355925050
64	H	−3.2142453510	−1.4612828165	−1.2983146133
65	H	−1.0281477003	−0.8011495596	−0.8146131726
66	H	−2.6371696552	0.6472433173	1.2837359415
67	H	1.7780594393	−0.7885192425	−1.8776049061
68	H	3.3490281045	0.4342290209	1.9122543215
69	H	5.9565685073	−1.3059910673	−1.0263213152
70	H	−0.0515584793	−1.1454417001	1.2375251852
71	H	−0.9833837251	1.9564497803	1.9567308706
		**HQ-TU_iii**		
		E = −2414.07045087777		
1	C	−2.0472176723	2.7391297619	4.3388877056
2	O	−1.9177478688	1.7519447045	3.3296379966
3	C	−2.9302395540	3.2813845434	1.7142749142
4	C	−2.0197256849	1.1229423093	1.0977799259
5	C	−2.3347132812	1.3560610595	−0.2633050401
6	C	−3.2673263215	3.5162843101	0.4075224712
7	C	−2.9784821317	2.5798741770	−0.6136882266
8	C	−1.9929820076	0.4641263545	−1.3317564238
9	C	−2.3207259237	0.8446959699	−2.6100774604
10	C	−2.9945383730	2.0611296750	−2.8404761637
11	N	−3.3160804872	2.9124305596	−1.8930633778
12	C	−1.2279218710	−0.8403157032	−1.1177963799
13	C	−2.1364668550	−2.0233120037	−1.4997799659
14	C	−3.2943029849	−2.3338939798	−0.5369332600
15	C	−3.4090602689	−3.8653722774	−0.4090469374
16	C	−3.4214142573	−4.4432286578	−1.8445418861
17	C	−1.9953168176	−4.2985127428	−2.4152148201

18	C	−3.9973330748	−5.8557421601	−2.0333455431
19	C	−3.3954809233	−6.9940971062	−1.2097057605
20	N	−1.2779625473	−3.2519285887	−1.6203494796
21	N	−0.0284118661	−0.9644666659	−1.9385795856
22	C	1.0862848458	−0.3963263291	−1.5644775176
23	N	1.0252656255	0.5070649830	−0.4899366592
24	C	1.9545976204	1.0458829315	0.3907376774
25	C	3.2744200169	0.6104442600	0.5490518441
26	C	4.0768014662	1.1889143997	1.5281290252
27	C	3.6093077967	2.1884327466	2.3715467437
28	C	1.4821632943	2.0627929888	1.2363244900
29	C	2.2946845679	2.6140364381	2.2120944763
30	C	5.5112584096	0.7481816240	1.6371722054
31	C	1.7243388214	3.6301434932	3.1623161153
32	F	6.0035374459	0.9405044648	2.8740164255
33	F	6.3073878917	1.4334276682	0.7932020541
34	F	5.6647387360	−0.5538403269	1.3463163831
35	F	0.6796432320	4.2919704214	2.6331193336
36	F	2.6391143793	4.5489484554	3.5217395421
37	F	1.2844094597	3.0588365344	4.2983524221
38	S	2.5837348643	−0.6958710690	−2.3847376632
39	H	−1.6191003060	2.2996349625	5.2396625371
40	H	−3.0988236086	2.9827193058	4.5290932020
41	H	−1.4906550413	3.6476062607	4.0876738548
42	C	−2.2916625413	2.0657894113	2.0674640736
43	H	−3.1522871391	4.0297893124	2.4654994084
44	H	−3.7558357756	4.4395769845	0.1141245571
45	H	−1.5290485928	0.2118873092	1.4197295374
46	H	−3.2637862355	2.3426257129	−3.8563427205
47	H	−2.0310644253	0.2288891366	−3.4557140419
48	H	−3.6303579682	−6.8983468684	−0.1451549668
49	H	−2.3065607645	−7.0631004043	−1.3124567519
50	H	−3.8055018823	−7.9534552150	−1.5415134309
51	H	−3.9114717919	−6.1073911648	−3.0986180667
52	H	−5.0731115446	−5.8079621019	−1.8264488116
53	C	−2.1836043806	−4.3446278828	0.3891111206
54	C	−0.9069872659	−3.8053705725	−0.2812531021
55	H	−4.0748983905	−3.7885968740	−2.4341079744
56	H	−1.4094914516	−5.2163920496	−2.3295405146
57	H	−1.9926961958	−3.9843132880	−3.4598357798
58	H	−4.3294529028	−4.1351522875	0.1164097787
59	H	−2.1468147355	−5.4323433685	0.4478871060
60	H	−2.2451916995	−3.9709825534	1.4146952162
61	H	−0.1569444814	−4.5776679450	−0.4550475883
62	H	−0.4299350165	−3.0037284522	0.2820639205
63	H	−3.1087206280	−1.8824678480	0.4444693818
64	H	−4.2162333675	−1.8938670328	−0.9248931612
65	H	−2.5041749932	−1.8506519368	−2.5141767247
66	H	−0.9886892604	−0.9372590897	−0.0503945595
67	H	3.6607531397	−0.1657197225	−0.0976640917
68	H	0.4608772045	2.4151313355	1.1291933539
69	H	4.2477459394	2.6213699238	3.1316459359

70	H	0.1022825223	0.8816107255	−0.3191077238
71	H	−0.4277241216	−2.9062109457	−2.1016125745
**HQ-TU_iv**				
E = −2414.06531469050				
1	C	−5.3083540352	1.7418740968	3.0718930307
2	O	−6.0575336090	2.3074431382	2.0072976050
3	C	−6.3116416872	2.8673585677	−0.2468412052
4	C	−4.2511234995	1.8127165469	0.4776748277
5	C	−3.7570269912	1.8552394466	−0.8562370984
6	C	−5.8544676846	2.9297443025	−1.5287759338
7	C	−4.5669149393	2.4335369695	−1.8743401813
8	C	−2.4721664134	1.3673211132	−1.2457093178
9	C	−2.0966882486	1.5104149854	−2.5615871448
10	C	−2.9786234196	2.0988812925	−3.4863415205
11	N	−4.1765702539	2.5449475085	−3.1734317875
12	C	−1.5317619152	0.6678700846	−0.2755217424
13	C	−1.6115024236	−0.8654284745	−0.4955866583
14	C	−2.9634956876	−1.5044737526	−0.0870371147
15	C	−2.6450720794	−2.8395646758	0.6142964012
16	C	−1.7089775320	−3.6410308376	−0.3222123661
17	C	−0.3523811960	−2.8863071648	−0.3614438264
18	C	−1.5619387145	−5.1487674211	−0.0641109750
19	C	−1.0543128133	−5.6001668147	1.3064558254
20	N	−0.4852711021	−1.5295664501	0.1784330803
21	N	−0.1280313086	1.0206196985	−0.4769332976
22	C	0.5194954957	2.1061807942	0.0273259355
23	N	1.7790539423	2.1785829836	0.2803958329
24	C	2.5554862873	1.0128756447	0.2157501521
25	C	3.4856199233	0.8421279490	−0.8119363439
26	C	4.2861344630	−0.2961980217	−0.8540425684
27	C	4.1840117806	−1.2793859281	0.1210990970
28	C	2.4553841159	0.0215983788	1.2030379816
29	C	3.2615186987	−1.1068147164	1.1490539989
30	C	5.3157219506	−0.4264800428	−1.9434601986
31	C	3.1289677631	−2.1899800896	2.1844810804
32	F	5.6804761628	−1.7039943552	−2.1434250795
33	F	6.4361472936	0.2602019380	−1.6486104002
34	F	4.8652665470	0.0506423563	−3.1161079497
35	F	2.4457618688	−1.7851423198	3.2668473842
36	F	4.3306603061	−2.6225507281	2.6067386646
37	F	2.4854786173	−3.2717132027	1.6984900839
38	S	−0.3910042748	3.5990779502	0.3856218307
39	H	−5.0961463397	0.6817768187	2.8893011994
40	H	−5.9342404015	1.8320340810	3.9596635959
41	H	−4.3718085684	2.2872456075	3.2356630480
42	C	−5.4999019699	2.3048270604	0.7753783925
43	H	−7.2916419786	3.2452180154	0.0241031868
44	H	−6.4542890086	3.3607553072	−2.3233200164
45	H	−3.6364656878	1.3952373924	1.2627714746
46	H	−2.6790020510	2.2036151833	−4.5266637031
47	H	−1.1157004113	1.1791839466	−2.8873548808
48	H	−0.8173331584	−6.6692186222	1.2862850704

49	H	−1.8053313629	−5.4556968563	2.0892889227
50	H	−0.1419575977	−5.0747315263	1.6115020667
51	H	−0.8819641456	−5.5415537324	−0.8325574544
52	H	−2.5306561811	−5.6293596786	−0.2520456879
53	C	−1.9644897065	−2.4791889304	1.9460283644
54	C	−0.7209351492	−1.6111568696	1.6252949260
55	H	−2.1553819689	−3.5689963027	−1.3240321596
56	H	0.4138942753	−3.3950982518	0.2307160523
57	H	0.0299750675	−2.8211683490	−1.3858459168
58	H	−3.5688597630	−3.3992441541	0.7969436347
59	H	−1.6789105153	−3.3700541226	2.5078480079
60	H	−2.6726195673	−1.9266814572	2.5738291766
61	H	0.1796543062	−2.0245725055	2.0841505387
62	H	−0.8299135575	−0.5943870546	2.0171397482
63	H	−3.5241661909	−0.8565545094	0.5959253211
64	H	−3.5953069816	−1.6552057047	−0.9675632155
65	H	−1.4446715430	−1.0244307903	−1.5678880229
66	H	−1.8191771451	0.9027199635	0.7539715614
67	H	3.5693436349	1.6069678312	−1.5762415472
68	H	1.7442868182	0.1496046445	2.0111490604
69	H	4.8124005807	−2.1612086012	0.0849219456
70	H	−1.4786919454	3.3144060272	−0.3481382024
71	H	0.4305128259	0.1628453899	−0.4458345897
		**HQ-TU_v**		
		E = −2414.05993970912		
1	C	−4.3522472976	3.7655586654	2.3319203383
2	O	−4.3617685898	4.3477653915	1.0368755022
3	C	−3.8393039982	4.2880077731	−1.2355535177
4	C	−3.2605946743	2.3862216524	0.1544253959
5	C	−2.6913439994	1.7323106780	−0.9675911834
6	C	−3.2985230383	3.6865701013	−2.3334713108
7	C	−2.7176774260	2.4060429555	−2.2093360875
8	C	−2.1099476381	0.4226044699	−0.9064080653
9	C	−1.5847181332	−0.1132462675	−2.0695408112
10	C	−1.6257810861	0.5914559878	−3.2620444655
11	N	−2.1778028712	1.7975344907	−3.3029362457
12	C	−2.1911510965	−0.3746488459	0.3897094157
13	C	−1.8039024702	−1.8664104639	0.2677578497
14	C	−2.7169009201	−2.6818016590	−0.6972042912
15	C	−2.9912141057	−4.0516025102	−0.0503888589
16	C	−1.6159940334	−4.6372710692	0.3465924212
17	C	−1.0852971420	−3.7657528615	1.5175978719
18	C	−1.5327993529	−6.1495723861	0.6058772923
19	C	−2.4656168350	−6.7523340421	1.6562247431
20	N	−1.7991372653	−2.4899636490	1.6064526557
21	N	−1.5211067080	0.3126019830	1.4896786847
22	C	−0.1718184459	0.6273269388	1.4602467867
23	N	0.3487430750	0.6181996210	0.2633879858
24	C	1.6716303716	0.8279551583	−0.0450766716
25	C	1.9730673636	1.6304148175	−1.1555906128
26	C	3.2807818792	1.7910619078	−1.5995966314
27	C	4.3332687600	1.1472563912	−0.9627272658

28	C	2.7456296462	0.1865823748	0.6011066275
29	C	4.0439535159	0.3449675493	0.1393597800
30	C	3.5394089858	2.6163190049	−2.8285295738
31	C	5.1826527296	−0.3223890113	0.8582007930
32	F	4.8153318995	3.0414528985	−2.8978251541
33	F	2.7549963962	3.7075738816	−2.8766338664
34	F	3.3006710364	1.9245706236	−3.9582429245
35	F	5.7981336749	0.5180542725	1.7143489066
36	F	6.1294964070	−0.7519396503	0.0023650115
37	F	4.7859984995	−1.3870400978	1.5751195681
38	S	0.5493429962	1.0629768273	2.9839289528
39	H	−3.3304884977	3.5620414994	2.6720321702
40	H	−4.9418316257	2.8419692459	2.3541840467
41	H	−4.8126010964	4.4995811111	2.9929255754
42	C	−3.8149374566	3.6384293507	0.0258807249
43	H	−4.2887479564	5.2722896699	−1.2995587924
44	H	−3.3118594636	4.1866483177	−3.2967460174
45	H	−3.1875000895	1.9059305432	1.1188913060
46	H	−1.2203371441	0.2024745170	−4.1877098928
47	H	−1.0961760831	−1.0755256467	−2.0687960710
48	H	−3.5088558174	−6.7496015491	1.3253448680
49	H	−2.4135511292	−6.2254651041	2.6161470783
50	H	−2.1932246167	−7.7957785568	1.8471871994
51	H	−0.4948463495	−6.3744338219	0.8878370583
52	H	−1.7017831522	−6.6714597311	−0.3447416552
53	C	−3.8886265361	−3.7947857398	1.1706877618
54	C	−3.1667642430	−2.7586630702	2.0700943605
55	H	−0.9526851934	−4.4638989170	−0.5128907983
56	H	−1.2076296173	−4.2784669229	2.4799081876
57	H	−0.0131361860	−3.5656258517	1.4043173705
58	H	−3.4954992753	−4.7130409462	−0.7635194707
59	H	−4.0936853442	−4.7140718554	1.7228224153
60	H	−4.8571048507	−3.4097571015	0.8317915621
61	H	−3.0992962332	−3.1088503980	3.1054385852
62	H	−3.7185334169	−1.8141573233	2.1075258588
63	H	−3.6652169310	−2.1582304538	−0.8787533191
64	H	−2.2406205675	−2.8234860739	−1.6716287592
65	H	−0.7609758215	−1.9003474523	−0.0695587811
66	H	−3.2581088917	−0.3478559230	0.6540514835
67	H	1.1554610078	2.1369122459	−1.6581203160
68	H	2.5449912096	−0.4336411034	1.4652035926
69	H	5.3516498172	1.2603860584	−1.3134896052
70	H	−1.8406131730	0.0162732556	2.3999061060
71	H	−2.2142853853	2.2860838254	−4.2009728778
**HQ-TU_vi**				
E = −2414.06002676725				
1	C	−7.1689617693	2.7020958911	2.2136538698
2	O	−5.9059250172	2.1023866264	1.9660443978
3	C	−5.8242459232	3.2189221949	−0.2060401674
4	C	−4.0739768375	1.7420738816	0.5838968042
5	C	−3.3404507301	1.9418532077	−0.6100684167
6	C	−5.1297346355	3.4281505092	−1.3681981556

7	C	−3.8803315424	2.8077768775	−1.6066259767
8	C	−2.0713475435	1.3388742454	−0.8838656015
9	C	−1.4614722775	1.6407027929	−2.0768255273
10	C	−2.0892175638	2.5066031238	−2.9966341443
11	N	−3.2542162421	3.0763834129	−2.7889417449
12	C	−1.3755318140	0.3913321598	0.0839406740
13	C	−1.4668928695	−1.0623176598	−0.4210678521
14	C	−2.9274692525	−1.5737034526	−0.5619434090
15	C	−2.9507154621	−3.0427101180	−0.1027214500
16	C	−1.8229147704	−3.7690586036	−0.8708241761
17	C	−0.4819096309	−3.2276406937	−0.3094044927
18	C	−1.8881048328	−5.3020543796	−0.9503312506
19	C	−1.9347851482	−6.0896236068	0.3589552737
20	N	−0.6721548545	−1.9687963179	0.4236986826
21	N	0.0231521929	0.7323400520	0.2058763088
22	C	0.4627430366	1.4967011876	1.1132173601
23	N	1.8129509750	1.6239924724	1.3570183497
24	C	2.8190300762	0.7597135344	0.9127510306
25	C	2.5815251960	−0.5819312694	0.5854153408
26	C	3.6531636304	−1.3875028492	0.2216069264
27	C	4.9564310793	−0.9035694640	0.1688469041
28	C	4.1234344462	1.2547761023	0.8627127891
29	C	5.1776014862	0.4259753900	0.4969981801
30	C	3.3698637121	−2.8183133788	−0.1487980466
31	C	6.5653096049	1.0047872957	0.4722841310
32	F	4.4905162785	−3.5599088924	−0.2045796907
33	F	2.7806135886	−2.9127929278	−1.3569102362
34	F	2.5477415244	−3.4093956545	0.7336830844
35	F	6.6351007052	2.0868065958	−0.3245541134
36	F	7.4781248090	0.1241725273	0.0305862618
37	F	6.9560806029	1.4039282347	1.6964889368
38	S	−0.4956603488	2.4784735491	2.2956974533
39	H	−7.9156097102	2.3875463925	1.4761013783
40	H	−7.0993577191	3.7954600323	2.2332227773
41	H	−7.4748472733	2.3497709628	3.1989574343
42	C	−5.2885952578	2.3622053321	0.7887160423
43	H	−6.7771237847	3.7131520536	−0.0591713321
44	H	−5.5191307391	4.0809090769	−2.1425575458
45	H	−3.7101244120	1.0882649424	1.3680786887
46	H	−1.5991682778	2.7335773432	−3.9410474867
47	H	−0.4854189872	1.2269924362	−2.3090174356
48	H	−2.8903598115	−5.9628424244	0.8775171624
49	H	−1.1341518322	−5.8015844551	1.0498828942
50	H	−1.8176946920	−7.1599770826	0.1587928537
51	H	−1.0134337161	−5.6336964021	−1.5270213485
52	H	−2.7633665217	−5.5791179720	−1.5522281464
53	C	−2.7008182752	−3.0306398287	1.4163713889
54	C	−1.3759646725	−2.2663102139	1.6765943418
55	H	−1.9026663851	−3.4304854461	−1.9140916680
56	H	−0.0219397192	−3.9420123343	0.3820809457
57	H	0.2432711634	−3.0618778109	−1.1121425662
58	H	−3.9222774788	−3.4976033028	−0.3263778388

59	H	−2.6501671829	−4.0431402269	1.8213734114
60	H	−3.5405644637	−2.5338558815	1.9156930105
61	H	−0.6966474838	−2.8510673736	2.3052266492
62	H	−1.5556068684	−1.3278602770	2.2106216035
63	H	−3.6157325395	−0.9846082996	0.0570588676
64	H	−3.2697527384	−1.4722340193	−1.5971245800
65	H	−0.9789799489	−1.0725015274	−1.4036478556
66	H	−1.8710299984	0.4458339131	1.0609394625
67	H	2.1269902899	2.4935462249	1.7735467056
68	H	1.5731969323	−0.9882786219	0.6075430832
69	H	4.3173528291	2.2952881523	1.1075623206
70	H	5.7772558027	−1.5473165031	−0.1177971570
71	H	−1.5213804070	2.7251405444	1.4668356318
		**HQ-TU_vii**		
		E = −2414.05158183387		
1	C	−2.6541411399	5.6105818238	1.4862387194
2	O	−2.7741919033	4.2025455741	1.4258906569
3	C	−2.5351549061	4.3086108619	−1.0016436028
4	C	−2.7803465315	2.2261755777	0.1874033153
5	C	−2.7227587402	1.4856996911	−0.9989564360
6	C	−2.5160700650	3.6030270903	−2.1917136399
7	C	−2.6340891208	2.2125540070	−2.2107201080
8	C	−2.7402118458	−0.0037469405	−1.0623402676
9	C	−3.1375538903	−0.5232859594	−2.3689672912
10	C	−3.0708091909	0.2415452921	−3.4782117285
11	N	−2.7041842293	1.5507370660	−3.4249626704
12	C	−2.3676321007	−0.8550900645	−0.0619767435
13	C	−2.3231398596	−2.3606397773	−0.2597032708
14	C	−3.6396050833	−3.1101950826	0.0935515340
15	C	−3.2572375563	−4.5462299371	0.5023026415
16	C	−2.2533268063	−5.0690712651	−0.5523520129
17	C	−0.9341526787	−4.2767651681	−0.3384158629
18	C	−2.0595725253	−6.5896481606	−0.6564695979
19	C	−1.6571262177	−7.3558420706	0.6034806594
20	N	−1.1716375181	−3.0248064557	0.3997003245
21	N	−1.9847763424	−0.3854046647	1.2328711537
22	C	−0.7604807442	0.0616177292	1.5924711412
23	N	0.1042580955	0.1807203386	0.5543453650
24	C	1.3878784346	0.7415105306	0.5056004585
25	C	1.7086850306	1.5061418381	−0.6161947201
26	C	2.9823816369	2.0448549839	−0.7541110748
27	C	3.9479849993	1.8401109424	0.2222781709
28	C	2.3558233921	0.5183072993	1.4856937554
29	C	3.6194500313	1.0756576145	1.3368303928
30	C	3.3239342390	2.8189534836	−1.9981732159
31	C	4.6439993686	0.8839377375	2.4218532022
32	F	4.3428703389	3.6725004581	−1.7941407516
33	F	2.2806840529	3.5377633678	−2.4414993507
34	F	3.6910035214	2.0037592320	−3.0037115734
35	F	4.6059028978	1.8839851400	3.3217183248
36	F	5.8937839640	0.8554545024	1.9265083916
37	F	4.4544224201	−0.2607170575	3.0979479914

38	S	−0.4364988211	0.4736601664	3.2050044025
39	H	−3.4618756165	6.1113874064	0.9381511334
40	H	−1.6833350717	5.9520335822	1.1062782571
41	H	−2.7292833728	5.8731256516	2.5421322614
42	C	−2.6943443658	3.6136667328	0.1998444080
43	H	−2.4457524425	5.3879704200	−1.0238446672
44	H	−2.4280337721	4.1432542722	−3.1300957310
45	H	−2.8885804945	1.7350751046	1.1430325884
46	H	−3.3032638125	−0.1470779859	−4.4623510308
47	H	−3.4719388403	−1.5454332189	−2.4771889233
48	H	−2.4632047497	−7.3766336034	1.3438429511
49	H	−0.7671905916	−6.9348632008	1.0852048986
50	H	−1.4251120066	−8.3963219770	0.3522308580
51	H	−1.3017698751	−6.7689046023	−1.4317209189
52	H	−2.9890700783	−7.0276984700	−1.0426687334
53	C	−2.6041477939	−4.4386072753	1.8902132875
54	C	−1.4639982694	−3.3913142818	1.7937611312
55	H	−2.6577247976	−4.7741963589	−1.5313595292
56	H	−0.2085388831	−4.8663856424	0.2354600305
57	H	−0.4613622606	−4.0447019470	−1.2997290053
58	H	−4.1443188280	−5.1890833249	0.5419631573
59	H	−2.2206290979	−5.4032769866	2.2292604986
60	H	−3.3547945687	−4.1249148425	2.6247940937
61	H	−0.5364898331	−3.7768336028	2.2312478092
62	H	−1.7063444770	−2.4893994556	2.3556709711
63	H	−4.1668811358	−2.6135343609	0.9190258750
64	H	−4.3199141096	−3.1018810374	−0.7659986366
65	H	−2.1358361267	−2.5111304639	−1.3260421530
66	H	0.9517520032	1.6945983876	−1.3711352550
67	H	2.1173143735	−0.0824925832	2.3518222542
68	H	4.9386511476	2.2650579755	0.1171693392
69	H	−2.7093779052	2.0992332647	−4.2772837748
70	H	−0.2904039195	−0.0721686720	−0.3483963974
71	H	−2.6359150340	−0.4800686486	2.0026522703
		**HQ-TU_viii**		
		E = −2414.04582841145		
1	C	−5.6814036148	2.8211006917	2.6814418451
2	O	−5.9915749194	3.5409848181	1.4985783150
3	C	−5.6043331072	4.0565241109	−0.7463924193
4	C	−4.2136200973	2.3878513662	0.3300159700
5	C	−3.4895029580	2.2016322381	−0.8791552359
6	C	−4.9238366357	3.8956903376	−1.9161265468
7	C	−3.8498875613	2.9700185111	−2.0188798015
8	C	−2.4075509317	1.2754838880	−1.0199293860
9	C	−1.7917155409	1.1988078132	−2.2449456267
10	C	−2.2289178752	2.0101232776	−3.3115859793
11	N	−3.2179060708	2.8700234372	−3.2217227435
12	C	−1.9552530417	0.4402582782	0.1768001035
13	C	−1.3142259853	−0.9030425997	−0.2180556664
14	C	−2.3876815514	−1.8449522806	−0.8464402789
15	C	−2.2362675684	−3.2295104464	−0.1942117193
16	C	−0.7725981726	−3.6684757066	−0.4240373688

17	C	0.1228016903	−2.6949378925	0.3896115116
18	C	−0.4227554610	−5.1487637533	−0.2043084941
19	C	−0.6824478164	−5.7546591945	1.1755269638
20	N	−0.6329530837	−1.5494239547	0.9108452080
21	N	−1.1846212250	1.2687072981	1.1169209464
22	C	0.0558535616	1.7480664241	1.0124810054
23	N	0.7687038937	1.3520859205	−0.0976332779
24	C	1.7581507472	0.5368126180	−0.0538174829
25	C	2.4287953052	0.2390418673	−1.3154836791
26	C	3.3941464002	−0.6937362003	−1.3762226712
27	C	3.8282136299	−1.4420356752	−0.2022544225
28	C	2.2248850998	−0.1513867274	1.2023028622
29	C	3.2840239071	−1.1858175886	0.9912289085
30	C	4.0746614410	−1.0308841502	−2.6746221107
31	C	3.6989187892	−1.9536655359	2.2123569662
32	F	5.4020816312	−0.8322421423	−2.5799831629
33	F	3.6262678741	−0.3017658651	−3.7024267759
34	F	3.8939431099	−2.3267488814	−2.9826463374
35	F	4.1785045946	−1.1264965433	3.1604152133
36	F	4.6453425725	−2.8672305869	1.9570219089
37	F	2.6514420476	−2.6008198958	2.7566867074
38	S	0.6928391225	2.8742703114	2.1056429124
39	H	−4.6619129199	3.0360487427	3.0225900068
40	H	−5.8099028454	1.7420929218	2.5366965674
41	H	−6.3886058008	3.1659583634	3.4358300498
42	C	−5.2465714134	3.2936830339	0.3968918275
43	H	−6.4241522490	4.7614405268	−0.6596091203
44	H	−5.1787003558	4.4670806654	−2.8024679022
45	H	−3.9528914667	1.8131757730	1.2080115852
46	H	−1.7331188708	1.9402119759	−4.2773149708
47	H	−0.9615654287	0.5261070722	−2.4205502111
48	H	−1.7492843148	−5.9149047903	1.3582936813
49	H	−0.2909213883	−5.1315858468	1.9881328118
50	H	−0.1949742822	−6.7318189039	1.2574359638
51	H	0.6445846000	−5.2628296781	−0.4384380294
52	H	−0.9558362135	−5.7469097645	−0.9543389276
53	C	−2.5816406321	−3.0572761986	1.2966207657
54	C	−1.5911089414	−2.0334801718	1.9099348983
55	H	−0.5729185056	−3.4891124456	−1.4894909597
56	H	0.5900534068	−3.1952542787	1.2442831444
57	H	0.9399699048	−2.3153792266	−0.2356596637
58	H	−2.9234699015	−3.9431933915	−0.6621454498
59	H	−2.5405329218	−4.0063382852	1.8336860786
60	H	−3.6142601792	−2.6986442257	1.3791781222
61	H	−1.0119243048	−2.4803103544	2.7242149819
62	H	−2.1104971869	−1.1736713065	2.3434163527
63	H	−3.4011082177	−1.4610398311	−0.6744898846
64	H	−2.2566830739	−1.9034864807	−1.9315032499
65	H	−0.5266490840	−0.7200780594	−0.9514606414
66	H	2.1011896424	0.7896412892	−2.1896639777
67	H	1.3361584647	−0.6107526234	1.6627387586
68	H	4.5896954558	−2.2039883046	−0.3196507111

69	H	−2.8531507121	0.1838998089	0.7448604358
70	H	2.5718034930	0.6121434445	1.9125968816
71	H	−1.7025659570	1.6282829632	1.9106046253
		**HQ-TU_ix**		
		E = −2414.041261351		
1	C	−4.9056757426	3.2794969777	2.5778769926
2	O	−5.1367462178	3.8984320336	1.3209344960
3	C	−4.7260316958	4.0988085015	−0.9655844280
4	C	−3.6490683699	2.3101502088	0.2702287444
5	C	−2.9979007765	1.8828593877	−0.9144768895
6	C	−4.1136424438	3.7108083607	−2.1212261281
7	C	−3.2411009361	2.6020250230	−2.1051946336
8	C	−2.1157311816	0.7511837368	−0.9576274866
9	C	−1.5373555149	0.4196734361	−2.1712327617
10	C	−1.7981295726	1.1676408137	−3.3120088300
11	N	−2.6197079300	2.2063668373	−3.2527846439
12	C	−1.9391759474	−0.0886444327	0.3073611530
13	C	−1.2382115651	−1.4396212226	0.0346312348
14	C	−2.1448506776	−2.3771965930	−0.8293089454
15	C	−2.3490959809	−3.6930446410	−0.0595474010
16	C	−0.9486933093	−4.3217010978	0.1041247400
17	C	−0.1056614383	−3.3233207308	0.9505633788
18	C	−0.8619924052	−5.7780389020	0.5904147541
19	C	−1.4331225473	−6.1222623742	1.9668227379
20	N	−0.8405965444	−2.1019970263	1.2855556478
21	N	−1.4681598181	0.6451780535	1.4830053342
22	C	−0.5508442884	1.5491644134	1.4101250889
23	N	0.1802496317	1.7328957661	0.1783286771
24	C	1.3088309685	0.9815569377	−0.1297155978
25	C	2.0499254220	1.3226173811	−1.2682212759
26	C	3.1859056937	0.6014037812	−1.6148898477
27	C	3.6113359554	−0.4738066518	−0.8474629503
28	C	1.7266273446	−0.1139054476	0.6439760715
29	C	2.8670546502	−0.8149061913	0.2795194521
30	C	3.9231721614	0.9576836764	−2.8766060916
31	C	3.3464650139	−1.9775949942	1.1042475293
32	F	5.2083747368	0.5598144028	−2.8397752098
33	F	3.9216161897	2.2827339497	−3.1039860027
34	F	3.3677278698	0.3801001556	−3.9567181594
35	F	4.6398004805	−1.8300937114	1.4540637664
36	F	3.2732836012	−3.1344439036	0.4151095773
37	F	2.6452774612	−2.1436122438	2.2346539972
38	S	−0.1528412998	2.6387871038	2.7041143665
39	H	−3.8451961514	3.3129415588	2.8527438770
40	H	−5.2518635465	2.2394692932	2.5762923381
41	H	−5.4882640671	3.8495460115	3.3014332976
42	C	−4.4880171224	3.4008298906	0.2468836018
43	H	−5.3971882114	4.9497723619	−0.9448310606
44	H	−4.2935600056	4.2501793613	−3.0459098200
45	H	−3.4208978634	1.7978908778	1.1957921619
46	H	−1.3576536393	0.9409334283	−4.2752401419
47	H	−0.8574353980	−0.4155190319	−2.2634287530

48	H	−2.5264927889	−6.1495432468	1.9642716610
49	H	−1.1163198281	−5.4131425161	2.7406348910
50	H	−1.0911660821	−7.1142812180	2.2805393506
51	H	0.2025384626	−6.0501685938	0.5812062111
52	H	−1.3396215690	−6.4243536511	−0.1573989868
53	C	−3.0084495964	−3.3259644204	1.2827751783
54	C	−2.0112593635	−2.4551235057	2.0895359898
55	H	−0.5174347456	−4.3446996339	−0.9062493138
56	H	0.2248333157	−3.7773295509	1.8927557313
57	H	0.8019332285	−3.0397708159	0.4114341338
58	H	−2.9995430777	−4.3648461809	−0.6309012558
59	H	−3.3048030461	−4.2106783920	1.8497356864
60	H	−3.9306411098	−2.7676532368	1.0780826485
61	H	−1.6458697549	−2.9932570474	2.9715478607
62	H	−2.4644901376	−1.5302833403	2.4589793321
63	H	−3.1180599750	−1.9098183817	−1.0254716330
64	H	−1.6869175107	−2.5819524193	−1.8031576691
65	H	−0.2940437398	−1.2483976629	−0.4860083477
66	H	−2.9745344964	−0.3613912753	0.5743243730
67	H	1.7353560287	2.1668163755	−1.8743533666
68	H	1.1509782854	−0.4182937977	1.5109701490
69	H	4.5049140224	−1.0269800283	−1.1101516908
70	H	−2.8034595448	2.7298926551	−4.1127734202
71	H	0.3144980502	2.7158753596	−0.0360334707
		**HQ-TU_x**		
		E = −2414.02181102982		
1	C	−5.2626247950	3.3933538714	2.0907917512
2	O	−5.3695241264	3.8802949908	0.7624902436
3	C	−4.7536699889	3.8287511550	−1.4913256027
4	C	−3.7368559339	2.2470951474	0.0407570309
5	C	−2.9730895585	1.6998138674	−1.0252016342
6	C	−4.0225630095	3.3257558409	−2.5260138627
7	C	−3.1088064699	2.2553196422	−2.3255448661
8	C	−2.0653227668	0.6065199755	−0.8614713285
9	C	−1.3575228750	0.1988822274	−1.9627408326
10	C	−1.5602343726	0.8282192315	−3.2078768978
11	N	−2.4051876713	1.8142190536	−3.4057568602
12	C	−1.9268942650	−0.0650694627	0.4973094140
13	C	−1.2969489894	−1.4692019983	0.4941727065
14	C	−2.0592009286	−2.4971937178	−0.3874450500
15	C	−2.0415596956	−3.8472571018	0.3547185341
16	C	−0.5736452984	−4.1121925405	0.7661536849
17	C	−0.2307078974	−3.0838240662	1.8774312060
18	C	−0.1824820347	−5.5573197156	1.1101841821
19	C	−0.9601556932	−6.2718228132	2.2154096798
20	N	−1.1803476884	−1.9659863387	1.8791134671
21	N	−1.2850523629	0.8360104359	1.4532811836
22	C	0.0269107088	1.1907253156	1.3719746353
23	N	0.8476941362	0.9320568083	0.3968097392
24	C	2.0845107461	1.5041849518	0.6408748329
25	C	3.2341213181	1.3781214467	−0.2422569192
26	C	3.4000608320	0.6450576445	−1.3511011133

27	C	1.7922783702	−0.0238327605	−3.1138169967
28	C	2.1987295395	2.2034122960	1.8059304758
29	C	2.4227864477	−0.2890816406	−1.9738047531
30	C	4.7109804669	0.7325928406	−2.0898687246
31	C	2.2964200742	−1.6578329449	−1.3502556045
32	F	5.2585822897	−0.4848319182	−2.2597669239
33	F	5.6233637354	1.4917545701	−1.4620549747
34	F	4.5513970719	1.2572984162	−3.3225298626
35	F	3.3278738621	−2.4518415295	−1.6880597510
36	F	1.1737395155	−2.2928511467	−1.7521000104
37	F	2.2691330540	−1.6145766391	−0.0107237855
38	S	0.7089795878	2.1805751122	2.6711389666
39	H	−4.2448478266	3.5152903991	2.4793792931
40	H	−5.5621602998	2.3406821633	2.1542475365
41	H	−5.9474826802	3.9964867692	2.6871349686
42	C	−4.6064090249	3.2880894799	−0.1858131129
43	H	−5.4516302840	4.6465891110	−1.6353792872
44	H	−4.1123591514	3.7262001434	−3.5304284768
45	H	−3.5992250441	1.8602705390	1.0403841964
46	H	−0.9996070325	0.4930672909	−4.0782873174
47	H	−0.6292756283	−0.5974318481	−1.8910260169
48	H	−1.9873964727	−6.4966912638	1.9115815508
49	H	−1.0015423582	−5.6889258948	3.1428195353
50	H	−0.4804989651	−7.2264490138	2.4562607349
51	H	0.8827332454	−5.5493549899	1.3806377022
52	H	−0.2502771213	−6.1594133086	0.1947688455
53	C	−2.9621043126	−3.6919855280	1.5761584276
54	C	−2.4716498028	−2.4589897615	2.3771930107
55	H	0.0373701199	−3.8541444431	−0.1083245000
56	H	−0.2612380753	−3.5468487212	2.8715743985
57	H	0.7803411547	−2.6896027592	1.7362592139
58	H	−2.4052953145	−4.6463963095	−0.3011796156
59	H	−2.9569485182	−4.5884978451	2.1992480194
60	H	−3.9943735893	−3.5516883308	1.2360871572
61	H	−2.3474882216	−2.6990822999	3.4381709229
62	H	−3.2032582567	−1.6462292594	2.3312002230
63	H	−3.0955050413	−2.1788677691	−0.5649953914
64	H	−1.5862139136	−2.5897513243	−1.3697503508
65	H	−0.2709505158	−1.3665422956	0.1356323277
66	H	4.0804761463	1.9840843246	0.0670431191
67	H	1.1464306584	−0.7535515538	−3.5902590520
68	H	−2.9496220561	−0.1741702405	0.8760819381
69	H	3.0711003114	2.7096387772	2.1959220413
70	H	−1.6185159825	0.7019674503	2.4010060162
71	H	1.9059885184	0.9343398051	−3.6073734370
**Q-TU_i**				
E = −2412.85477732600				
1	C	−8.7329492995	3.0988204951	−2.8273382563
2	O	−9.2884512473	2.3015561712	−1.7943594600
3	C	−9.1184910454	1.2176810314	0.2697870611
4	C	−7.1819895409	2.3833853067	−0.6067154199
5	C	−6.4316984759	2.0126020397	0.5431081974

6	C	−8.4152119807	0.8390430808	1.3740723214
7	C	−7.0557604459	1.2188476162	1.5455126521
8	C	−5.0725465569	2.3876694969	0.7602391615
9	C	−4.4600063153	1.9466001569	1.9088036052
10	C	−5.1664496294	1.1530222441	2.8295875320
11	N	−6.4226636419	0.7946426485	2.6741642075
12	C	−4.3210698323	3.3253665747	−0.1687383999
13	C	−4.3021634082	4.7504022250	0.4517012070
14	C	−5.6607591976	5.4859686982	0.4202047121
15	C	−5.3701641871	6.9658403435	0.1199545936
16	C	−4.2599894851	7.4644492078	1.0829537796
17	C	−2.9571734939	6.7072653950	0.7043052834
18	C	−4.1674420642	8.9675236784	1.0981356035
19	C	−3.0911860026	9.7284135391	0.9206683036
20	N	−3.2506091116	5.5693364950	−0.1729185651
21	N	−2.9236308333	2.9744871155	−0.3341363442
22	C	−2.4353374653	1.9446144397	−1.0405757774
23	N	−1.0745821237	1.9456048982	−1.2041506712
24	C	−0.1742872021	2.9559291987	−0.8000006295
25	C	−0.1792743927	3.4528343789	0.5066811950
26	C	0.6716616034	4.4919169454	0.8533290058
27	C	1.5800959654	5.0103489034	−0.0640913069
28	C	0.7371838034	3.4654732662	−1.7229935485
29	C	1.6166572798	4.4756085667	−1.3445639582
30	C	0.6486643473	5.0362609305	2.2565428823
31	C	2.6174464689	4.9811029053	−2.3491540510
32	F	0.8974100036	6.3561140973	2.2777925158
33	F	1.5765319077	4.4478555586	3.0333138396
34	F	−0.5411297181	4.8420378198	2.8516264142
35	F	3.6440809795	4.1227801405	−2.4905534106
36	F	3.1369874684	6.1665444939	−1.9917015147
37	F	2.0659906411	5.1322618076	−3.5649660473
38	S	−3.3789301235	0.7046056205	−1.7034889628
39	H	−8.4156221962	4.0773367419	−2.4476689815
40	H	−9.5295768790	3.2408398768	−3.5580022277
41	H	−7.8871591992	2.5958655480	−3.3109553541
42	C	−8.4943445533	1.9974851128	−0.7403573229
43	H	−10.1560368373	0.9319781870	0.1331018347
44	H	−8.8688391920	0.2382942131	2.1553896475
45	H	−6.7058721108	2.9554408308	−1.3901867547
46	H	−4.6722907394	0.8056931670	3.7344811485
47	H	−3.4250709561	2.2034927539	2.1112137706
48	H	−2.0978861548	9.3274693000	0.7395196032
49	H	−3.1621817996	10.8112931083	0.9673449178
50	H	−5.1179358734	9.4670278344	1.2925163305
51	C	−4.8626725403	7.0745650405	−1.3245798130
52	C	−3.6901391277	6.0731074464	−1.4835561795
53	H	−4.5609526591	7.1636353495	2.0978353968
54	H	−2.2625263920	7.3530750646	0.1592879085
55	H	−2.4326812569	6.3445536989	1.5928938034
56	H	−6.2777762713	7.5629047357	0.2545145132
57	H	−4.5340543188	8.0993573429	−1.5288212375

58	H	−5.6693279128	6.8469434298	−2.0292053873
59	H	−2.8279955755	6.5374994427	−1.9707713755
60	H	−3.9749642168	5.2152474513	−2.1007088882
61	H	−6.3187792464	5.0777444968	−0.3553143439
62	H	−6.1842316106	5.3646987917	1.3732628606
63	H	−3.9862314290	4.6200352989	1.4939268625
64	H	−4.8011250248	3.3417134387	−1.1522615717
65	H	−2.3612654920	3.8313455413	−0.2778132222
66	H	−0.7256704215	1.2617817185	−1.8649212726
67	H	−0.8610659986	3.0399258380	1.2396659435
68	H	0.7390072206	3.0886861200	−2.7408069140
69	H	2.2401351164	5.8232855492	0.2121085551
		**Q-TU_ii**		
		E = −2412.84445313069		
1	C	−6.7328772091	1.3352837622	1.7650637712
2	O	−6.8415225987	2.2712945131	0.7035067446
3	C	−5.9218292010	3.4128910024	−1.1184177999
4	C	−4.5178722949	1.8963336879	0.1487889677
5	C	−3.4199489858	2.1814307971	−0.7111965125
6	C	−4.8839642433	3.7077915326	−1.9508844931
7	C	−3.6080414449	3.1029044824	−1.7792171575
8	C	−2.1281567569	1.5873220070	−0.5802659755
9	C	−1.1604261362	1.9417028359	−1.4922695845
10	C	−1.4532537942	2.8688732993	−2.5080019778
11	N	−2.6277595010	3.4418384918	−2.6618013269
12	C	−1.7864924881	0.5866348362	0.5234381272
13	C	−1.6229696648	−0.8055253218	−0.1159981340
14	C	−2.9653109412	−1.4388833277	−0.5458020191
15	C	−3.2191570270	−2.6975268461	0.2953265923
16	C	−2.1311138552	−3.7491623929	−0.0438279500
17	C	−0.7562170611	−3.0763311449	0.1521222975
18	C	−2.3608532250	−5.0263476587	0.7257454915
19	C	−1.5917670283	−5.5922914743	1.6507857381
20	N	−0.9422474309	−1.7534437486	0.8250619345
21	N	−0.6936569465	1.0436336873	1.3960892244
22	C	0.6717549965	1.0730532528	1.1365975632
23	N	1.1014498892	0.0766445882	0.3909258084
24	C	2.4233880364	−0.1693263334	0.0552830369
25	C	2.6735626233	−0.6609515669	−1.2326761380
26	C	3.9523565349	−1.0352052198	−1.6291915307
27	C	5.0231408041	−0.9457500330	−0.7501912735
28	C	3.5104600664	−0.0841442125	0.9415983064
29	C	4.7809021376	−0.4716464736	0.5363426226
30	C	4.1658507675	−1.5016883375	−3.0415832179
31	C	5.9346014685	−0.3373194328	1.4907050346
32	F	5.3331097903	−2.1524290165	−3.1936289442
33	F	4.1781407911	−0.4695589291	−3.9097649487
34	F	3.1913429774	−2.3316689363	−3.4494859380
35	F	6.5642929230	0.8472155885	1.3517045027
36	F	6.8643979149	−1.2900176389	1.2902999836
37	F	5.5480582293	−0.4240730075	2.7741457751
38	S	1.5546265942	2.3828773875	1.8483849865

39	H	−7.7198101998	1.2875229648	2.2253608135
40	H	−6.0039894973	1.6645506644	2.5146476025
41	H	−6.4639299724	0.3404041334	1.3908674241
42	C	−5.7406373362	2.4909254834	−0.0526659172
43	H	−6.8980424382	3.8678278782	−1.2477673663
44	H	−4.9979879596	4.4079502473	−2.7717651803
45	H	−4.3909582068	1.2079680710	0.9723501094
46	H	−0.6806076342	3.1471647949	−3.2213958190
47	H	−0.1628440662	1.5188555628	−1.4289963023
48	H	−1.8946547068	−6.5150755051	2.1361539771
49	H	−0.6321763110	−5.1930101445	1.9675801839
50	H	−3.3044646119	−5.5139616071	0.4796553461
51	C	−3.1346263837	−2.3280411077	1.7831295912
52	C	−1.6847593268	−1.9314653208	2.1129075837
53	H	−2.2398788794	−3.9943898827	−1.1067001317
54	H	−0.0695957151	−3.6528378731	0.7710536025
55	H	−0.2558540595	−2.8795934884	−0.7967128842
56	H	−4.2069966058	−3.1037519492	0.0630004592
57	H	−3.4359461182	−3.1698502480	2.4100641991
58	H	−3.8197552240	−1.5012154204	1.9953646914
59	H	−1.1600500292	−2.7140328612	2.6627132427
60	H	−1.5954259821	−1.0012107222	2.6722642591
61	H	−3.7824675706	−0.7249168389	−0.4095954528
62	H	−2.9388956288	−1.6855159103	−1.6105366536
63	H	−0.9333953248	−0.7193781063	−0.9544800480
64	H	−2.6352334907	0.5223598862	1.2055910120
65	H	1.8424451732	−0.7464148401	−1.9257954942
66	H	3.3504555089	0.2855627371	1.9455276802
67	H	6.0171669045	−1.2523078075	−1.0507709103
68	H	−0.0078895149	−1.3152316094	0.9604868794
69	H	−0.9866135036	1.8263941196	1.9642919159
		**Q-TU_iii**		
		E = −2412.83046539741		
1	C	−2.0628524877	2.6571011541	4.3668800949
2	O	−1.9100888493	1.6848602515	3.3463835686
3	C	−2.9511413275	3.2114300530	1.7466394300
4	C	−1.9925729215	1.0807842697	1.1068794783
5	C	−2.3072246422	1.3242776226	−0.2524870552
6	C	−3.2880443169	3.4555970158	0.4415461554
7	C	−2.9747063233	2.5388878887	−0.5902844484
8	C	−1.9430731855	0.4531919672	−1.3306371252
9	C	−2.2718029830	0.8437742606	−2.6056233241
10	C	−2.9688609194	2.0493324726	−2.8238368751
11	N	−3.3128142438	2.8811188546	−1.8669961947
12	C	−1.1554814067	−0.8399986120	−1.1292183389
13	C	−2.0424035360	−2.0331540850	−1.5290607364
14	C	−3.2002701915	−2.3741639887	−0.5805955425
15	C	−3.2918117127	−3.9053370326	−0.4568297237
16	C	−3.2793120287	−4.5068115984	−1.8774671585
17	C	−1.8602835617	−4.2889874944	−2.4696773390
18	C	−3.6689444872	−5.9580322443	−1.9974172208
19	C	−4.1658526786	−6.7386713212	−1.0431784804

20	N	−1.1661926597	−3.2472059730	−1.6538754229
21	N	0.0489249163	−0.9359526321	−1.9462474432
22	C	1.1511798347	−0.3478537722	−1.5656514397
23	N	1.0690016591	0.5463709338	−0.4854798129
24	C	1.9843031671	1.1086512583	0.3954171049
25	C	3.3198193302	0.7201295009	0.5429385620
26	C	4.1063392177	1.3200441268	1.5222015768
27	C	3.6082078824	2.2958977392	2.3755723826
28	C	1.4803272267	2.1011521074	1.2519724621
29	C	2.2779608064	2.6746678462	2.2268532816
30	C	5.5565030166	0.9306581935	1.6192183159
31	C	1.6783067808	3.6647920201	3.1864256811
32	F	6.0502658093	1.1344609407	2.8536381613
33	F	6.3214924152	1.6481670861	0.7732685182
34	F	5.7546882027	−0.3635350465	1.3205931146
35	F	0.6072622397	4.2919363384	2.6677476783
36	F	2.5622354149	4.6136874231	3.5448731513
37	F	1.2666204283	3.0720789024	4.3220602250
38	S	2.6559872776	−0.6122591430	−2.3844641715
39	H	−1.6252870515	2.2171764273	5.2629032679
40	H	−3.1199222904	2.8743057768	4.5588784712
41	H	−1.5272939620	3.5809084858	4.1265034045
42	C	−2.2874114731	2.0057501980	2.0870680266
43	H	−3.1923099661	3.9452346445	2.5063248228
44	H	−3.7951937353	4.3718586555	0.1578886591
45	H	−1.4835076916	0.1766171553	1.4195840555
46	H	−3.2385314963	2.3387532334	−3.8373467687
47	H	−1.9659499067	0.2445997773	−3.4574959727
48	H	−4.3465714523	−6.3940807005	−0.0287920876
49	H	−4.4240431877	−7.7735758749	−1.2465095193
50	H	−3.5248863672	−6.3849071096	−2.9906489208
51	C	−2.0659491247	−4.4027690577	0.3254500307
52	C	−0.7972027621	−3.8146405483	−0.3183957611
53	H	−3.9898066356	−3.9359044857	−2.4899609573
54	H	−1.2504166686	−5.1931540844	−2.4164476574
55	H	−1.8826075383	−3.9431652141	−3.5037167788
56	H	−4.2133063654	−4.1766083916	0.0631129651
57	H	−2.0274270163	−5.4949112231	0.3149831266
58	H	−2.1285351607	−4.0873511782	1.3699559693
59	H	−0.0222880166	−4.5611769381	−0.4944606660
60	H	−0.3536465282	−3.0084615874	0.2652722220
61	H	−3.0325122810	−1.9286770466	0.4065874371
62	H	−4.1285111374	−1.9500406057	−0.9710126067
63	H	−2.4054654696	−1.8566874359	−2.5446004472
64	H	−0.9195193072	−0.9460660114	−0.0618650543
65	H	3.7298501135	−0.0368306446	−0.1119235739
66	H	0.4463736963	2.4169607511	1.1530386000
67	H	4.2350441892	2.7466365898	3.1349549177
68	H	0.1355149249	0.8902409832	−0.3076476210
69	H	−0.3166814950	−2.8860318241	−2.1255826729

Q-TU_iv

E = −2412.82611248451

1	C	−5.2407870436	1.7172668207	3.1024198103
2	O	−6.0136766335	2.2475179311	2.0365876126
3	C	−6.3034155855	2.7647568767	−0.2233790175
4	C	−4.2133953495	1.7658845587	0.4956370324
5	C	−3.7341512129	1.7969528312	−0.8440882253
6	C	−5.8606552357	2.8167766801	−1.5108218005
7	C	−4.5662093295	2.3430108039	−1.8621965634
8	C	−2.4441694251	1.3278795530	−1.2400491292
9	C	−2.0847855989	1.4594399454	−2.5615737925
10	C	−2.9871715948	2.0176070197	−3.4852966654
11	N	−4.1909974889	2.4434343837	−3.1665354814
12	C	−1.4818202255	0.6569103891	−0.2704197711
13	C	−1.5427672680	−0.8785303110	−0.4753988789
14	C	−2.8847522081	−1.5235616167	−0.0548081758
15	C	−2.5613527791	−2.8593752196	0.6365557083
16	C	−1.6189301401	−3.6646047693	−0.2815236936
17	C	−0.2636437901	−2.8837700016	−0.3386907176
18	C	−1.3962136340	−5.1075188363	0.0823309891
19	C	−2.0310270141	−5.8175284093	1.0107139723
20	N	−0.4118539089	−1.5314814243	0.2013824308
21	N	−0.0872765674	1.0332097021	−0.4872536915
22	C	0.5389360795	2.1435125880	−0.0103900112
23	N	1.7999435968	2.2558845052	0.2191684715
24	C	2.6109384428	1.1143154432	0.1608407361
25	C	3.5509891021	0.9701502554	−0.8619611965
26	C	4.3868484038	−0.1426740137	−0.8966603788
27	C	4.3110087153	−1.1254252596	0.0813327286
28	C	2.5376749895	0.1240071993	1.1514424388
29	C	3.3791564486	−0.9782823125	1.1047335231
30	C	5.4248980268	−0.2448486410	−1.9809616719
31	C	3.2748570186	−2.0630368465	2.1412209993
32	F	5.8210727056	−1.5127126681	−2.1829337141
33	F	6.5271765103	0.4671872180	−1.6775954262
34	F	4.9697842505	0.2253702217	−3.1545860233
35	F	2.5631040288	−1.6819454383	3.2142910280
36	F	4.4873293075	−2.4472450421	2.5802944542
37	F	2.6808250201	−3.1682110566	1.6488100831
38	S	−0.4036923066	3.6170464106	0.3454713077
39	H	−5.0087812129	0.6586037643	2.9365175961
40	H	−5.8582405614	1.8107611436	3.9957233553
41	H	−4.3140734735	2.2848324189	3.2451710203
42	C	−5.4691993365	2.2363422037	0.7988965321
43	H	−7.2885813579	3.1257208519	0.0518604841
44	H	−6.4778304012	3.2223146237	−2.3054837775
45	H	−3.5821070416	1.3750188719	1.2811745789
46	H	−2.7000753173	2.1136037547	−4.5299503675
47	H	−1.1009567291	1.1424254424	−2.8929169734
48	H	−1.7872137367	−6.8621781158	1.1815985907
49	H	−2.8186983610	−5.4025624835	1.6338578396
50	H	−0.6198424870	−5.6039553022	−0.5022199912
51	C	−1.8527544255	−2.5437933349	1.9637868337
52	C	−0.6497605403	−1.6176973499	1.6484741084

53	H	−2.0540185859	−3.6589112718	−1.2918609998
54	H	0.5050572489	−3.3889631739	0.2536302331
55	H	0.1089465856	−2.8168945784	−1.3658370647
56	H	−3.4880161002	−3.4130405422	0.8153346534
57	H	−1.5137078208	−3.4668842831	2.4439263262
58	H	−2.5503389770	−2.0579453522	2.6544595395
59	H	0.2665075576	−1.9842460594	2.1164100338
60	H	−0.8120033299	−0.6049521338	2.0317025862
61	H	−3.4376554514	−0.8826056854	0.6407179793
62	H	−3.5295907753	−1.6711882393	−0.9262913231
63	H	−1.3790079269	−1.0462753791	−1.5468468232
64	H	−1.7649167230	0.8970416877	0.7590113424
65	H	3.6142117192	1.7350254875	−1.6280710799
66	H	1.8203598785	0.2330410309	1.9568463011
67	H	4.9660746595	−1.9878809983	0.0507309531
68	H	−1.5010426770	3.2920527348	−0.3562686597
69	H	0.4922873937	0.1905207967	−0.4518431278
		**DQ-TU_i**		
		E = −2411.59906088590		
1	C	−8.7123459248	3.0856011623	−2.8020087155
2	O	−9.2352651831	2.2893506840	−1.7512589804
3	C	−9.0164901905	1.2305392826	0.3205046544
4	C	−7.1077941458	2.4060836389	−0.6026866294
5	C	−6.3346255691	2.0564482922	0.5392192959
6	C	−8.2906592074	0.8705901988	1.4163824536
7	C	−6.9326338108	1.2669360941	1.5606247983
8	C	−4.9765314119	2.4506353175	0.7298991217
9	C	−4.3385804018	2.0260070172	1.8707570615
10	C	−5.0199032714	1.2343351117	2.8123191975
11	N	−6.2750298822	0.8621076981	2.6825636348
12	C	−4.2555866674	3.3965409731	−0.2166735523
13	C	−4.2275310088	4.8150966145	0.4069917143
14	C	−5.6132392146	5.4964406021	0.4846117805
15	C	−5.4344324544	6.9672870529	0.0811458926
16	C	−4.2922024631	7.5725787714	0.9418177777
17	C	−2.9675235477	6.8309297282	0.5421448510
18	C	−4.1813966867	9.0215504028	0.7845213112
19	C	−4.0813657334	10.2131072395	0.6298693610
20	N	−3.2562176478	5.6696966968	−0.2941625689
21	N	−2.8651193515	3.0429338887	−0.4301370499
22	C	−2.4229171968	2.0207377148	−1.1808002135
23	N	−1.0692131263	1.9808826307	−1.3727481404
24	C	−0.0980986594	2.8686220218	−0.8635634187
25	C	−0.0897101948	3.2557421025	0.4780411014
26	C	0.8786994122	4.1396828391	0.9370864697
27	C	1.8741951947	4.6157933338	0.0928369152
28	C	0.8978286835	3.3428689496	−1.7176848491
29	C	1.8809213862	4.1979180192	−1.2322774117
30	C	0.8283271658	4.5521185243	2.3835872840
31	C	2.9483446342	4.6714936078	−2.1821646584
32	F	1.8159236240	5.4009259906	2.7070932532
33	F	0.9264715751	3.4873447088	3.1999232446

34	F	−0.3350979014	5.1587836493	2.6817689006
35	F	3.6942343573	3.6444173587	−2.6275323342
36	F	3.7831948151	5.5544985513	−1.6114984555
37	F	2.4162846595	5.2628355841	−3.2654599196
38	S	−3.4207339395	0.8364209725	−1.8655979630
39	H	−8.3985744130	4.0709475478	−2.4374546131
40	H	−9.5266983407	3.2124709791	−3.5157903073
41	H	−7.8714383350	2.5899117664	−3.3011933011
42	C	−8.4184296632	2.0060224320	−0.7088152090
43	H	−10.0528878768	0.9320980834	0.2049397851
44	H	−8.7246856399	0.2738270106	2.2116323511
45	H	−6.6494024915	2.9687262321	−1.4038783632
46	H	−4.5066848715	0.9019653106	3.7123598238
47	H	−3.3036194917	2.2968197313	2.0532608084
48	H	−3.9967666517	11.2708626563	0.5086182873
49	C	−5.0367490392	7.0192264050	−1.3996878651
50	C	−3.7841951927	6.1231006585	−1.5896603585
51	H	−4.5171854960	7.3669732878	1.9958660359
52	H	−2.3116950305	7.5012554807	−0.0197730735
53	H	−2.4222372584	6.5046924607	1.4316830298
54	H	−6.3587202591	7.5245200678	0.2525694274
55	H	−4.8341346715	8.0533897384	−1.6976259952
56	H	−5.8685526019	6.6631712983	−2.0161211302
57	H	−2.9823384775	6.6602267599	−2.1041092635
58	H	−4.0089042942	5.2409927867	−2.1966789554
59	H	−6.3295362366	5.0242352317	−0.1959387201
60	H	−6.0309174397	5.4050681546	1.4914249621
61	H	−3.8258334694	4.6934968452	1.4203864757
62	H	−4.7674329617	3.4164133071	−1.1834217919
63	H	−2.2829279792	3.8841895444	−0.3800792716
64	H	−0.7543018495	1.3090555695	−2.0621422436
65	H	−0.8376714902	2.8671364201	1.1592621360
66	H	0.8921379199	3.0492843767	−2.7627465729
67	H	2.6300828872	5.2977968311	0.4590586989
		**DQ-TU_ii**		
		E = −2411.58672334335		
1	C	−6.6506206426	1.5234210319	1.9293884206
2	O	−6.7839813192	2.3945674438	0.8169603389
3	C	−5.9164973930	3.4172309770	−1.0970588968
4	C	−4.4988789000	1.9206295881	0.1799242580
5	C	−3.4313451700	2.1376071878	−0.7367342699
6	C	−4.9081975596	3.6459051751	−1.9846406670
7	C	−3.6405683167	3.0183745169	−1.8350961854
8	C	−2.1491346390	1.5180734463	−0.6309284131
9	C	−1.2071899102	1.8185004074	−1.5887591648
10	C	−1.5188984997	2.7105916486	−2.6298254172
11	N	−2.6876358712	3.2993487530	−2.7662719384
12	C	−1.7887889587	0.5538094751	0.4991215931
13	C	−1.5865991589	−0.8502546967	−0.1030696367
14	C	−2.9153214105	−1.5344064018	−0.4946770815
15	C	−3.1222971740	−2.7776498004	0.3814944827
16	C	−2.0046992621	−3.7970850275	0.0392883066

17	C	−0.6364684081	−3.0800542930	0.2088430439
18	C	−2.0910688761	−5.0080423110	0.8591758198
19	C	−2.1571574951	−5.9887844875	1.5555692301
20	N	−0.8615689238	−1.7512669607	0.8518164052
21	N	−0.7171839795	1.0669230152	1.3666227426
22	C	0.6463897772	1.1347829735	1.1095168829
23	N	1.1077203001	0.1380980557	0.3827075381
24	C	2.4382940375	−0.0782110549	0.0601869459
25	C	2.7103578094	−0.5959173524	−1.2120548555
26	C	4.0012054325	−0.9492079752	−1.5901552800
27	C	5.0622422493	−0.8110556916	−0.7068548148
28	C	3.5168171529	0.0590609456	0.9511054034
29	C	4.7988269699	−0.3079453079	0.5652193381
30	C	4.2154920766	−1.4910727693	−2.9752235297
31	C	5.9399234294	−0.1215323873	1.5260775846
32	F	5.4911533301	−1.8573963117	−3.1917605788
33	F	3.8997934168	−0.5821446236	−3.9190652441
34	F	3.4473791057	−2.5682469522	−3.2141475088
35	F	6.5294106401	1.0825028981	1.3782063312
36	F	6.9035824715	−1.0438601829	1.3442043450
37	F	5.5462340177	−0.2070290728	2.8075220356
38	S	1.4876583827	2.4818463305	1.8012459380
39	H	−7.6160254448	1.5334924434	2.4352988330
40	H	−5.8794519489	1.8775383924	2.6233501415
41	H	−6.4260881264	0.4995806090	1.6079442223
42	C	−5.7124133268	2.5420109979	0.0033712771
43	H	−6.8855716538	3.8922980492	−1.2065489893
44	H	−5.0387900704	4.3108702748	−2.8319739331
45	H	−4.3550981131	1.2683307832	1.0298097027
46	H	−0.7676832756	2.9449412389	−3.3806813126
47	H	−0.2134954159	1.3840512003	−1.5416382346
48	H	−2.2113682043	−6.8664545115	2.1618732671
49	C	−3.0194151712	−2.3762795735	1.8589611888
50	C	−1.5777153980	−1.9252401744	2.1556953116
51	H	−2.1143644530	−4.0901226733	−1.0096391747
52	H	0.0488854165	−3.6400170318	0.8445908291
53	H	−0.1494821000	−2.8964464571	−0.7492617763
54	H	−4.0967921938	−3.2244544587	0.1752827653
55	H	−3.2858226914	−3.2151551667	2.5050336442
56	H	−3.7276264421	−1.5682901204	2.0652512877
57	H	−1.0178797405	−2.6764513243	2.7146591571
58	H	−1.5128585166	−0.9795202654	2.6918179567
59	H	−3.7511859648	−0.8420892090	−0.3598519758
60	H	−2.9007076619	−1.8058509487	−1.5533913408
61	H	−0.9126660098	−0.7686030229	−0.9544994398
62	H	−2.6401089028	0.4800825858	1.1768127544
63	H	1.8866951066	−0.7184134638	−1.9091018594
64	H	3.3392005453	0.4531558596	1.9429225939
65	H	6.0656928710	−1.0999214224	−0.9922180252
66	H	0.0602587606	−1.2754386488	0.9623406547
67	H	−1.0393906057	1.8536458292	1.9131969727

DQ-TU_iii

E = −2411.57319198321				
1	C	−2.0079619972	2.7935082010	4.3425449354
2	O	−1.8830018506	1.7950237529	3.3439984551
3	C	−2.9084730116	3.3040864818	1.7176348481
4	C	−1.9933944309	1.1427207905	1.1197273487
5	C	−2.3140990796	1.3617067620	−0.2421886898
6	C	−3.2513255516	3.5249534301	0.4099044479
7	C	−2.9630719651	2.5794312874	−0.6029892131
8	C	−1.9721942510	0.4602494079	−1.3020893315
9	C	−2.3033563372	0.8261760658	−2.5837890392
10	C	−2.9835168014	2.0371475893	−2.8244631680
11	N	−3.3059107400	2.8968300113	−1.8848218898
12	C	−1.2059162830	−0.8407083367	−1.0732068165
13	C	−2.1175473594	−2.0254425711	−1.4399814664
14	C	−3.2787242414	−2.3189340993	−0.4797002585
15	C	−3.3984267302	−3.8421897301	−0.3139897331
16	C	−3.3942204979	−4.4849555317	−1.7283381974
17	C	−1.9757674397	−4.2993412363	−2.3376190849
18	C	−3.7712924372	−5.8977610171	−1.7005490794
19	C	−4.0612982649	−7.0658524024	−1.6568336869
20	N	−1.2650851935	−3.2591585602	−1.5382512568
21	N	−0.0083274722	−0.9788684175	−1.8937375995
22	C	1.1055424777	−0.3992340436	−1.5340798707
23	N	1.0446335986	0.5221306803	−0.4750813841
24	C	1.9755650265	1.0818967288	0.3909290212
25	C	3.2993010088	0.6584692132	0.5486626651
26	C	4.1035206581	1.2589063131	1.5130946489
27	C	3.6345887326	2.2696493923	2.3421742353
28	C	1.5013581232	2.1096289155	1.2224589785
29	C	2.3159312958	2.6831791785	2.1835824856
30	C	5.5414653362	0.8288515190	1.6205354888
31	C	1.7447526951	3.7111851176	3.1203255928
32	F	6.0405732330	1.0447093253	2.8507659833
33	F	6.3279136824	1.5048801444	0.7602115876
34	F	5.7011181301	−0.4769305444	1.3503364870
35	F	0.6874440365	4.3515073060	2.5900028745
36	F	2.6531345510	4.6465911111	3.4521897058
37	F	1.3230972533	3.1573200147	4.2717505562
38	S	2.6001894464	−0.7043041545	−2.3563916055
39	H	−1.5721920282	2.3659411045	5.2453953440
40	H	−3.0587689532	3.0365859846	4.5376712602
41	H	−1.4560141947	3.7004239766	4.0761622923
42	C	−2.2641503441	2.0944743975	2.0806191995
43	H	−3.1299068356	4.0589426118	2.4625354285
44	H	−3.7437413403	4.4437084226	0.1093494285
45	H	−1.4975494237	0.2371502354	1.4489766822
46	H	−3.2576976723	2.3069535345	−3.8421882251
47	H	−2.0129743021	0.2035237030	−3.4242824392
48	H	−4.3284091630	−8.0995743681	−1.6276270803
49	C	−2.1828734623	−4.3561148358	0.4676504726
50	C	−0.9044686254	−3.8065514386	−0.1908569084
51	H	−4.1233869167	−3.9522687748	−2.3491040299

52	H	−1.3863824708	−5.2149845966	−2.2829277416
53	H	−2.0105417424	−3.9630391738	−3.3737119841
54	H	−4.3239314428	−4.1020187676	0.2025948756
55	H	−2.1780831966	−5.4498732282	0.4736250730
56	H	−2.2346819156	−4.0243890988	1.5073163888
57	H	−0.1436225179	−4.5706326504	−0.3516544585
58	H	−0.4463201929	−2.9957821675	0.3750376505
59	H	−3.0989035540	−1.8550779959	0.4964544219
60	H	−4.2001830809	−1.8866239843	−0.8766365028
61	H	−2.4814947019	−1.8667197808	−2.4581205559
62	H	−0.9651973192	−0.9249882989	−0.0051798120
63	H	3.6873057107	−0.1261054545	−0.0868463714
64	H	0.4769115877	2.4529176710	1.1163075942
65	H	4.2746322758	2.7197166245	3.0910139199
66	H	0.1206491033	0.8951293027	−0.3062535885
67	H	−0.4095206304	−2.9222010496	−2.0187893091
**DQ-TU_iv**				
E = −2411.57071498267				
1	C	−5.2739395851	1.8438054960	3.0714790753
2	O	−6.0312092185	2.3803126668	1.9975732105
3	C	−6.3001451991	2.8829237803	−0.2682705149
4	C	−4.2377584947	1.8402575180	0.4683205354
5	C	−3.7524790121	1.8485597189	−0.8695590303
6	C	−5.8522718642	2.9108641160	−1.5546818126
7	C	−4.5684388694	2.4024726768	−1.8964979312
8	C	−2.4704245258	1.3503737543	−1.2550314434
9	C	−2.1041320482	1.4584277602	−2.5768173293
10	C	−2.9918492570	2.0237535714	−3.5103809298
11	N	−4.1868458184	2.4800863000	−3.2005566370
12	C	−1.5218864071	0.6758416244	−0.2737632890
13	C	−1.5992395513	−0.8596948517	−0.4681330089
14	C	−2.9499883800	−1.4837173488	−0.0423743389
15	C	−2.6469028380	−2.7991448548	0.6922538094
16	C	−1.7071413616	−3.6517281501	−0.2024323228
17	C	−0.3449839033	−2.8781468412	−0.3103424516
18	C	−1.5282465679	−5.0105956743	0.3052543288
19	C	−1.3650176091	−6.1217801857	0.7438200454
20	N	−0.4762495448	−1.5221660394	0.2135947570
21	N	−0.1224812189	1.0381173063	−0.4834523764
22	C	0.5084247449	2.1419270448	0.0031526671
23	N	1.7670727862	2.2436632156	0.2482054422
24	C	2.5701254803	1.0964005179	0.1979175409
25	C	3.5124846970	0.9414808661	−0.8209847038
26	C	4.3408873880	−0.1769746307	−0.8477901368
27	C	4.2542786844	−1.1553337363	0.1337512079
28	C	2.4855608470	0.1107304468	1.1919405628
29	C	3.3192081291	−0.9978208728	1.1526058943
30	C	5.3821495326	−0.2898618830	−1.9278966023
31	C	3.2001998623	−2.0790231739	2.1910419150
32	F	5.7779248569	−1.5597973871	−2.1172012388
33	F	6.4837790241	0.4243407341	−1.6271791007
34	F	4.9309365904	0.1703316016	−3.1070077226

35	F	2.4957277443	−1.6850508510	3.2641625693
36	F	4.4070387642	−2.4813978561	2.6289590411
37	F	2.5871396557	−3.1753389301	1.7010645686
38	S	−0.4251099230	3.6240917559	0.3483949128
39	H	−5.0683917864	0.7777959437	2.9188833603
40	H	−5.8904307469	1.9638128306	3.9621341570
41	H	−4.3335964930	2.3896143676	3.2096812039
42	C	−5.4828181228	2.3440464126	0.7622209946
43	H	−7.2767605307	3.2712919422	−0.0000847786
44	H	−6.4567451987	3.3227821574	−2.3558434878
45	H	−3.6192497864	1.4409814053	1.2598246926
46	H	−2.6997524211	2.1004854710	−4.5552956151
47	H	−1.1259322302	1.1177347780	−2.9013899264
48	H	−1.2206611977	−7.1100677402	1.1223211204
49	C	−1.9260173013	−2.4701326685	2.0058686619
50	C	−0.7021900339	−1.5816491354	1.6647324402
51	H	−2.1604289252	−3.7237754239	−1.1988429993
52	H	0.4354733237	−3.3857115382	0.2611637124
53	H	−0.0144310007	−2.8299446305	−1.3518141533
54	H	−3.5715351691	−3.3487676887	0.8858030903
55	H	−1.6143708581	−3.3953565260	2.5021004727
56	H	−2.6090792306	−1.9527438306	2.6872588137
57	H	0.2087521766	−1.9655926651	2.1286439056
58	H	−0.8337275768	−0.5594143600	2.0335235682
59	H	−3.5050687256	−0.8220212882	0.6311199191
60	H	−3.5887254610	−1.6517645848	−0.9146173113
61	H	−1.4370082104	−1.0382214739	−1.5379236652
62	H	−1.8089208536	0.9270591889	0.7517627414
63	H	3.5838325798	1.7028392228	−1.5898779655
64	H	1.7665142347	0.2278833209	1.9945725117
65	H	4.9035846173	−2.0223009351	0.1092938115
66	H	−1.5136400150	3.3137486133	−0.3734973013
67	H	0.4512473531	0.1916696297	−0.4502411348
**HQ-TU_anion_1**				
E = −2413.59162169682				
1	C	−8.6608067202	3.1969609062	−2.7620504370
2	O	−9.0701891977	2.2506480855	−1.7891693839
3	C	−8.6861761818	1.0380529079	0.1734525812
4	C	−6.9456083884	2.5048272519	−0.6603445039
5	C	−6.1084374071	2.1429342522	0.4317265989
6	C	−7.8985378569	0.6678172768	1.2229003879
7	C	−6.5911713783	1.2042103695	1.3859387000
8	C	−4.7980973318	2.6752778813	0.6284816249
9	C	−4.0880100401	2.2219518456	1.7171124752
10	C	−4.6574743298	1.2857518302	2.5990901485
11	N	−5.8661405634	0.7840125965	2.4591173886
12	C	−4.1762413894	3.7327616740	−0.2763473655
13	C	−4.2576620009	5.1087286713	0.4216897764
14	C	−5.6871602118	5.5097371491	0.8857512912
15	C	−5.8433327728	7.0257354989	0.6697197353
16	C	−4.6184744569	7.7048592382	1.3252455556
17	C	−3.3891686193	7.3250316791	0.4560152479

18	C	−4.7206168690	9.2047679091	1.6426386712
19	C	−5.0529103357	10.1612828080	0.4976968026
20	N	−3.6688529364	6.1719837688	−0.4082484782
21	N	−2.7898554572	3.4292143113	−0.5333048872
22	C	−2.4488290654	2.9315220591	−1.6714661935
23	N	−1.0740696947	2.7250476653	−1.8816575606
24	C	0.0287132470	3.1428582273	−1.1594065212
25	C	−0.0188582533	3.9871977894	−0.0397689982
26	C	1.1592693361	4.3684645262	0.5938227557
27	C	2.4048124117	3.9395424162	0.1570655148
28	C	1.2920046637	2.7103994425	−1.6049868321
29	C	2.4507251771	3.1061400932	−0.9579824382
30	C	1.0501530723	5.2429516072	1.8137669783
31	C	3.7813142703	2.5808596934	−1.4221024035
32	F	2.2347397670	5.7646950024	2.1750519195
33	F	0.5852514030	4.5592122775	2.8766766925
34	F	0.2074041919	6.2745872023	1.6241886326
35	F	4.1526686759	1.4845748861	−0.7330036457
36	F	4.7610554372	3.4871628773	−1.2568226668
37	F	3.7723994915	2.2371680637	−2.7210230319
38	S	−3.4385858057	2.4389768512	−3.0323506477
39	H	−8.4654063724	4.1758020455	−2.3069512836
40	H	−9.4930952736	3.2851275599	−3.4610754073
41	H	−7.7663879372	2.8618731764	−3.3007289098
42	C	−8.2038595562	1.9676911646	−0.7867537969
43	H	−9.6843375859	0.6331221139	0.0432485914
44	H	−8.2425979064	−0.0439508386	1.9663504559
45	H	−6.5626177234	3.1860082617	−1.4074442484
46	H	−4.0853785497	0.9397913374	3.4579813637
47	H	−3.0760459216	2.5778716245	1.8813646082
48	H	−6.0859052888	10.0428303306	0.1554703153
49	H	−4.3932246247	10.0234044707	−0.3667487937
50	H	−4.9397389347	11.1987226033	0.8304401511
51	H	−3.7615919029	9.5071412591	2.0857041186
52	H	−5.4704627895	9.3379758218	2.4333369834
53	C	−5.8807836044	7.2419403033	−0.8540288059
54	C	−4.6039600428	6.5951033709	−1.4544526888
55	H	−4.4909848322	7.2151943403	2.3018159639
56	H	−3.0945939079	8.1580841034	−0.1955278737
57	H	−2.5196732623	7.0913629306	1.0815511668
58	H	−6.7719418034	7.3835927392	1.1293071636
59	H	−5.9379976416	8.3019741971	−1.1099145545
60	H	−6.7823731358	6.7692175616	−1.2616505122
61	H	−4.0739178727	7.2975864877	−2.1073631285
62	H	−4.8435055411	5.7253633139	−2.0751451290
63	H	−6.4536246685	4.9755465986	0.3105467476
64	H	−5.8365442669	5.2364924971	1.9360933997
65	H	−3.6060192679	5.0294667958	1.3034104481
66	H	−4.7381149614	3.7725968076	−1.2168735660
67	H	−0.8722969515	2.2461747301	−2.7482313275
68	H	−0.9832433593	4.3352394204	0.3026513084
69	H	1.3543594774	2.0620383558	−2.4736804843

70	H	3.3124491286	4.2504269239	0.6581322407
**HQ-TU_anion_2**				
E = −2413.58689071442				
1	C	−8.6553627649	3.1391067314	−2.8510860638
2	O	−9.2249043402	2.3857249214	−1.7942193464
3	C	−9.0773533067	1.3707539009	0.3065819349
4	C	−7.1177685540	2.4671112795	−0.6058221179
5	C	−6.3737957939	2.1213935772	0.5562608588
6	C	−8.3800629857	1.0124948134	1.4216907691
7	C	−7.0129050769	1.3695630639	1.5809754146
8	C	−5.0078408651	2.4836866942	0.7592996761
9	C	−4.4049112181	2.0595215036	1.9198741481
10	C	−5.1246081259	1.3050982407	2.8624672935
11	N	−6.3874817548	0.9650800631	2.7215492728
12	C	−4.2427907022	3.4062301237	−0.1779479997
13	C	−4.2389259283	4.8294442200	0.4411673196
14	C	−5.6337516741	5.4966808296	0.5446429062
15	C	−5.4424582706	6.9975719372	0.2551058100
16	C	−4.2860132967	7.4913056830	1.1571872000
17	C	−2.9856357057	6.8532052469	0.6012821190
18	C	−4.1795883582	9.0031266535	1.4095211533
19	C	−4.0771338687	9.9291520876	0.1979190407
20	N	−3.2877683839	5.7048463729	−0.2647829771
21	N	−2.8476282105	3.0478502773	−0.3314853583
22	C	−2.3441727684	2.0587367629	−1.1528834828
23	N	−1.0616233230	2.0630332542	−1.4636937083
24	C	−0.1620407118	2.9548329001	−0.9096763377
25	C	−0.0838731411	3.3126570489	0.4521056120
26	C	0.8800436944	4.2036988328	0.9125678567
27	C	1.8242344801	4.7568134208	0.0593058651
28	C	0.8225513070	3.5066058139	−1.7586191381
29	C	1.7838183341	4.3845783926	−1.2842234533
30	C	0.8613835202	4.5668977583	2.3713159519
31	C	2.8373402265	4.9121398382	−2.2184075206
32	F	1.7811746452	5.4933120384	2.6848234957
33	F	1.0952087855	3.4982590461	3.1579731870
34	F	−0.3356560535	5.0573559128	2.7494350853
35	F	3.9400614675	4.1364626456	−2.2186674308
36	F	3.2413982823	6.1481192130	−1.8743865889
37	F	2.4109184953	4.9750855654	−3.4913745334
38	S	−3.4210006600	0.8541283260	−1.7776300047
39	H	−8.3207525642	4.1238883637	−2.5030216584
40	H	−9.4496973509	3.2698246510	−3.5867189193
41	H	−7.8149471434	2.6099339403	−3.3160163790
42	C	−8.4370887854	2.1030373533	−0.7283462399
43	H	−10.1205748377	1.1015330486	0.1787391524
44	H	−8.8436087999	0.4454225209	2.2224062039
45	H	−6.6207246063	2.9922289988	−1.4091012955
46	H	−4.6321216485	0.9745824710	3.7750208493
47	H	−3.3620788517	2.2988623110	2.1020618311
48	H	−5.0134746832	9.9660594264	−0.3679231380
49	H	−3.2777685454	9.6311202183	−0.4903411816

50	H	−3.8575494613	10.9515111423	0.5240913984
51	H	−3.3013138747	9.1639832572	2.0497911028
52	H	−5.0460494368	9.3115556966	2.0088331994
53	C	−5.0961355793	7.1106332522	−1.2409385101
54	C	−3.8727760434	6.1978318423	−1.5173042700
55	H	−4.4667311826	7.0498847229	2.1477111726
56	H	−2.4126565941	7.5705563743	0.0014407927
57	H	−2.3269096576	6.5180892474	1.4095575904
58	H	−6.3631725867	7.5485143836	0.4772953699
59	H	−4.8809270434	8.1422471816	−1.5261355217
60	H	−5.9586608133	6.7909506773	−1.8370043872
61	H	−3.0884260836	6.7315561455	−2.0633693277
62	H	−4.1419620769	5.3323411316	−2.1315313783
63	H	−6.3352332626	5.0642312988	−0.1784896962
64	H	−6.0615919552	5.3286555759	1.5381460288
65	H	−3.8239290946	4.7088764568	1.4502986644
66	H	−4.7337917219	3.4198994026	−1.1579237390
67	H	−2.2917647779	3.9018763429	−0.3090146535
68	H	−0.8040549125	2.9003105831	1.1501515528
69	H	0.7910128145	3.2297260103	−2.8068440589
70	H	2.5626467651	5.4599319803	0.4221775369
		**HQ-TU_anion_3**		
		E = −2413.55324498076		
1	C	−7.1451777305	2.6507145863	2.2678783842
2	O	−5.8739618442	2.0740936846	2.0072736014
3	C	−5.8738654378	3.1387142475	−0.1920315581
4	C	−4.0661493541	1.7276499145	0.5868606920
5	C	−3.3654090483	1.9182129597	−0.6287413371
6	C	−5.2128634336	3.3391980394	−1.3751781919
7	C	−3.9534382048	2.7466181720	−1.6302294641
8	C	−2.0862718344	1.3414755597	−0.9154602799
9	C	−1.5174658115	1.6356067859	−2.1309386744
10	C	−2.1911259838	2.4596559950	−3.0552417985
11	N	−3.3650269350	3.0052453902	−2.8339590702
12	C	−1.3297542952	0.4275945305	0.0458902503
13	C	−1.4616293060	−1.0387093130	−0.4308800525
14	C	−2.9301802980	−1.5346238760	−0.5599992898
15	C	−2.9711244158	−3.0026899175	−0.0980193652
16	C	−1.8564655893	−3.7470488840	−0.8683335021
17	C	−0.5076941717	−3.2187231327	−0.3122289550
18	C	−1.9434984469	−5.2790583874	−0.9433332652
19	C	−1.9982429366	−6.0621286630	0.3680692136
20	N	−0.6797198149	−1.9539418878	0.4132782610
21	N	0.0713829547	0.7528074950	0.0970741968
22	C	0.5975489796	1.5381338346	0.9718574695
23	N	1.8887099318	1.7473477336	1.2293234653
24	C	2.8356891003	0.8289458604	0.8384268275
25	C	2.6232201948	−0.5254102480	0.4826356181
26	C	3.6968617323	−1.3610434244	0.1942940893
27	C	5.0133680510	−0.9143821872	0.2160965172
28	C	4.1811943544	1.2626587353	0.8692835807
29	C	5.2350851123	0.4174055668	0.5610195881

30	C	3.3975520246	−2.7851734600	−0.1850681266
31	C	6.6463576962	0.9205350118	0.6678314016
32	F	4.5071645347	−3.5439517042	−0.2554076310
33	F	2.7967011949	−2.8772099399	−1.3900659433
34	F	2.5749609513	−3.3840518169	0.6953321302
35	F	6.7447390382	2.2379978937	0.4145183009
36	F	7.4735413607	0.2927254603	−0.1889070096
37	F	7.1614855236	0.7292627956	1.8995623172
38	S	−0.3755369964	2.6285387883	2.1055174188
39	H	−7.9004190008	2.3003017592	1.5553363646
40	H	−7.1012745640	3.7457323439	2.2590189563
41	H	−7.4199361187	2.3162096912	3.2687034171
42	C	−5.2909210739	2.3205612629	0.8082708654
43	H	−6.8353948689	3.6119183272	−0.0328169369
44	H	−5.6365762524	3.9634699554	−2.1552320557
45	H	−3.6567353635	1.1082257184	1.3767304057
46	H	−1.7278615952	2.6752387543	−4.0161342897
47	H	−0.5307335215	1.2491802780	−2.3651427924
48	H	−2.9498427547	−5.9170215093	0.8893564227
49	H	−1.1906047012	−5.7831041985	1.0545258228
50	H	−1.8990576236	−7.1348043747	0.1696108742
51	H	−1.0740425666	−5.6228318241	−1.5209537641
52	H	−2.8244650438	−5.5446330872	−1.5423817974
53	C	−2.7150580165	−2.9880120515	1.4202987998
54	C	−1.3804833004	−2.2352263874	1.6687560894
55	H	−1.9350144569	−3.4075018445	−1.9116552699
56	H	−0.0542154274	−3.9334124995	0.3843331699
57	H	0.2219687257	−3.0663285309	−1.1142931302
58	H	−3.9502095860	−3.4447862725	−0.3158399327
59	H	−2.6719309408	−3.9994037300	1.8296450955
60	H	−3.5467281974	−2.4785987959	1.9211202439
61	H	−0.7037528778	−2.8216424279	2.2996481797
62	H	−1.5435347891	−1.2884943531	2.1946093951
63	H	−3.6058993770	−0.9348754218	0.0625475740
64	H	−3.2781743253	−1.4302692128	−1.5935219569
65	H	−0.9798348761	−1.0681611417	−1.4172505896
66	H	−1.7945192122	0.4967563983	1.0401269322
67	H	1.6091498696	−0.9117774611	0.4376397598
68	H	4.3637835746	2.2968063958	1.1428806215
69	H	5.8356502305	−1.5739408580	−0.0290930401
70	H	−1.5577638150	2.6040678988	1.4651387566
		**HQ-TU_anion_4**		
		E = −2413.56789398267		
1	C	−6.6588577328	1.3123333588	1.9870170943
2	O	−6.7158310102	2.3365633412	1.0079189148
3	C	−5.7382470011	3.5807922989	−0.7127699965
4	C	−4.3995770966	1.9228266730	0.4427563048
5	C	−3.2817108757	2.2379754141	−0.3804258228
6	C	−4.6797255915	3.9088003334	−1.5060989797
7	C	−3.4249172139	3.2528456721	−1.3685061839
8	C	−2.0165156259	1.5831005894	−0.2826121434
9	C	−1.0224223290	1.9989343364	−1.1398349135

10	C	−1.2659021968	3.0196305558	−2.0748107284
11	N	−2.4207036929	3.6372828619	−2.2030585580
12	C	−1.7091356556	0.4659328276	0.7183825869
13	C	−1.4869680898	−0.8378989865	−0.1106041012
14	C	−2.8219055349	−1.2677640732	−0.7958141864
15	C	−3.1233835189	−2.7239206197	−0.4048558807
16	C	−1.9467809102	−3.5748501917	−0.9288047744
17	C	−0.6784377662	−3.1003042119	−0.1682754133
18	C	−2.1154133182	−5.1030591504	−0.9521694631
19	C	−2.3976491086	−5.8221566697	0.3675410473
20	N	−0.9462007089	−1.9436194467	0.7024432920
21	N	−0.7950691471	1.0147355428	1.7213639616
22	C	0.4949354686	0.8970838145	1.7539736111
23	N	1.1184812350	−0.0833058837	0.9454983679
24	C	2.4345691495	−0.2836202277	0.5848048193
25	C	2.8322879671	−1.5942302339	0.2849852471
26	C	4.1033053516	−1.8658042105	−0.2067830403
27	C	5.0261948183	−0.8494102095	−0.4018952385
28	C	3.3697155245	0.7480509142	0.3875386069
29	C	4.6364438198	0.4536042458	−0.0942592402
30	C	4.4538377546	−3.2953667357	−0.5122760737
31	C	5.6397302754	1.5613069783	−0.2626303150
32	F	5.6646344903	−3.4167999511	−1.0814034891
33	F	3.5644929114	−3.8585603138	−1.3499735154
34	F	4.4632961537	−4.0563667025	0.5991054841
35	F	6.4657694381	1.3330785493	−1.2998066284
36	F	6.4231968687	1.6996246684	0.8244994214
37	F	5.0571739028	2.7534592182	−0.4767523745
38	S	1.4684540578	1.8530823454	2.8419270363
39	H	−7.6537493496	1.2681671580	2.4309621422
40	H	−5.9249562249	1.5427207362	2.7682328120
41	H	−6.4214110067	0.3419700835	1.5346765488
42	C	−5.5991456712	2.5732278184	0.2793181208
43	H	−6.6983831242	4.0752409338	−0.8158143783
44	H	−4.7592918574	4.6761918374	−2.2692122144
45	H	−4.2876182247	1.1740602302	1.2144740784
46	H	−0.4684391943	3.3354299606	−2.7445161596
47	H	−0.0355489371	1.5482765891	−1.0873326833
48	H	−2.2618288960	−6.9019671040	0.2448611475
49	H	−3.4255342892	−5.6660722457	0.7076112354
50	H	−1.7249700996	−5.5019823385	1.1720046588
51	H	−1.1935498014	−5.5163800328	−1.3837397701
52	H	−2.9138639029	−5.3506951518	−1.6633440075
53	C	−3.2564070449	−2.7507120059	1.1302536739
54	C	−1.8933243502	−2.3489178436	1.7482566000
55	H	−1.8253262711	−3.2886238422	−1.9820217320
56	H	−0.2738717537	−3.8952549882	0.4691689318
57	H	0.1172146683	−2.8219749587	−0.8689841519
58	H	−4.0603249408	−3.0497242481	−0.8703107247
59	H	−3.5772560297	−3.7272807601	1.4965131256
60	H	−4.0343404158	−2.0352849015	1.4239614065
61	H	−1.4378856746	−3.1860514673	2.2868989554

62	H	−1.9861669633	−1.5293913717	2.4670413233
63	H	−3.6481700964	−0.6217460957	−0.4761981155
64	H	−2.7449344909	−1.1604266156	−1.8827400639
65	H	−0.7288100273	−0.6432029327	−0.8767389368
66	H	−2.6249484486	0.2660538450	1.2852079644
67	H	2.1284836562	−2.4071210393	0.4401368396
68	H	3.0896322938	1.7661475686	0.6212668059
69	H	6.0137640111	−1.0578836986	−0.7925286649
70	H	0.5366933946	−0.9313888397	0.9080494970

**Q-TU_anion_1**				
E = −2412.35344501182				
1	C	−8.7391547333	3.1510590595	−2.7585389400
2	O	−9.1902081649	2.2243809764	−1.7851071836
3	C	−8.8608329206	1.0011150747	0.1810556902
4	C	−7.0578094754	2.3895280399	−0.6547055439
5	C	−6.2388871320	1.9971880875	0.4407998041
6	C	−8.0909964069	0.6008438920	1.2326202441
7	C	−6.7622711974	1.0815841418	1.3962634361
8	C	−4.9080332639	2.4747582153	0.6404747425
9	C	−4.2197061657	1.9937363629	1.7312039720
10	C	−4.8291251793	1.0828937395	2.6127202555
11	N	−6.0576587013	0.6325958931	2.4714825946
12	C	−4.2400166052	3.5080196364	−0.2584710465
13	C	−4.2881274655	4.8890905070	0.4378219904
14	C	−5.7115421447	5.3675071963	0.8292580269
15	C	−5.7786385876	6.8848285020	0.6009594389
16	C	−4.5563946059	7.5449448665	1.2861631057
17	C	−3.2965795510	7.0555434374	0.5166307192
18	C	−4.7157952086	9.0370259110	1.4139451433
19	C	−3.8365478662	9.9857468671	1.1026062486
20	N	−3.6056879301	5.9198520330	−0.3573992381
21	N	−2.8568983520	3.1771372242	−0.4851894166
22	C	−2.4902338839	2.6690105635	−1.6095332890
23	N	−1.1018633409	2.5205476009	−1.7905810363
24	C	−0.0540994467	3.1651506203	−1.1550804156
25	C	−0.2104359544	4.1967372719	−0.2153422857
26	C	0.9075066614	4.8055525916	0.3403745301
27	C	2.2007919326	4.4299946964	−0.0011112628
28	C	1.2538296433	2.7855229667	−1.5038627243
29	C	2.3538191352	3.4113696445	−0.9367773437
30	C	0.6842214905	5.9432403964	1.2996547576
31	C	3.7369246336	2.9332732975	−1.2819696303
32	F	1.7823828463	6.2228169607	2.0227738968
33	F	−0.3064079485	5.6813756027	2.1740560878
34	F	0.3398540014	7.0780509485	0.6621863394
35	F	4.1522005084	1.9587685890	−0.4495902721
36	F	4.6448533224	3.9215816025	−1.1986096818
37	F	3.8075434151	2.4322595759	−2.5273175400
38	S	−3.4499816879	2.1080310163	−2.9626160193
39	H	−8.4990357155	4.1200574286	−2.3039088433
40	H	−9.5668429310	3.2768591668	−3.4572344010

41	H	−7.8615812365	2.7749955741	−3.2977356390
42	C	−8.3379239792	1.9065733940	−0.7809680375
43	H	−9.8752586596	0.6389136391	0.0506994393
44	H	−8.4657478727	−0.0938041145	1.9774570293
45	H	−6.6463497855	3.0524879912	−1.4030402425
46	H	−4.2736740654	0.7139830141	3.4729710861
47	H	−3.1946819650	2.3096364522	1.8961736081
48	H	−4.0719737724	11.0344343305	1.2612767767
49	H	−2.8510316564	9.7731075712	0.6968222916
50	H	−5.6740810897	9.3469855780	1.8345534657
51	C	−5.7083454479	7.1439023213	−0.9103342989
52	C	−4.4607901668	6.3938681839	−1.4507426369
53	H	−4.5180634140	7.1479452342	2.3127508152
54	H	−2.8949796379	7.8473081980	−0.1253549804
55	H	−2.4961991466	6.7652605927	1.2033236910
56	H	−6.7077864160	7.2901756690	1.0166959412
57	H	−5.6357906013	8.2204840731	−1.1035710973
58	H	−6.6218838928	6.7858051951	−1.3981345757
59	H	−3.8542453296	7.0426030637	−2.0922456353
60	H	−4.7417799871	5.5322798794	−2.0653204236
61	H	−6.4778581186	4.8746479300	0.2183960972
62	H	−5.9301063992	5.1110716735	1.8717211083
63	H	−3.6838770043	4.7772802519	1.3490684618
64	H	−4.7839086932	3.5589271236	−1.2100203268
65	H	−0.8537755742	1.9867451595	−2.6118952943
66	H	−1.2114347100	4.5022833949	0.0567163508
67	H	1.4006380218	1.9922159839	−2.2307811947
68	H	3.0615155752	4.9131324377	0.4432533109
		**Q-TU_anion_2**		
		E = −2412.34826184821		
1	C	−8.7009389212	3.0337948509	−2.8384491940
2	O	−9.2506193923	2.2762836562	−1.7739858849
3	C	−9.0741769087	1.2788744154	0.3329031678
4	C	−7.1391384310	2.4037134964	−0.5970914808
5	C	−6.3836390759	2.0800783209	0.5639178815
6	C	−8.3653052264	0.9417344352	1.4473812148
7	C	−7.0043227359	1.3253336486	1.5976056304
8	C	−5.0234544624	2.4685551209	0.7573135524
9	C	−4.4082854147	2.0674626519	1.9195956694
10	C	−5.1100404176	1.3083928560	2.8719809158
11	N	−6.3665214572	0.9426675377	2.7389402190
12	C	−4.2795073014	3.3949782442	−0.1929823159
13	C	−4.3001468157	4.8246248448	0.4103355212
14	C	−5.7031962567	5.4651430966	0.5214354600
15	C	−5.5489108215	6.9627206528	0.2125334615
16	C	−4.3823822520	7.5315006212	1.0624760473
17	C	−3.0783242679	6.8707806734	0.5358525829
18	C	−4.3982955467	9.0369275572	1.0938309340
19	C	−3.4156159504	9.8802049625	0.7889456967
20	N	−3.3786413487	5.7133865482	−0.3151751148
21	N	−2.8784082597	3.0618840437	−0.3471319362
22	C	−2.3570001827	2.0663565951	−1.1492274664

23	N	−1.0741363964	2.0877008391	−1.4580429833
24	C	−0.1910715296	3.0018607051	−0.9140307557
25	C	−0.1230376115	3.3784851457	0.4433474158
26	C	0.8260635961	4.2892926178	0.8956842723
27	C	1.7647128191	4.8458180252	0.0384198559
28	C	0.7872893669	3.5585107064	−1.7669531231
29	C	1.7338012558	4.4566558430	−1.3004804737
30	C	0.7974578119	4.6698639361	2.3498369477
31	C	2.7818350077	4.9880039948	−2.2386858797
32	F	1.7066265087	5.6091373215	2.6559594542
33	F	1.0381079107	3.6131956978	3.1503953223
34	F	−0.4061423897	5.1531355826	2.7167680490
35	F	3.8930558933	4.2245970176	−2.2319990441
36	F	3.1719338778	6.2314009457	−1.9054315238
37	F	2.3551166088	5.0350665357	−3.5122760920
38	S	−3.4118249640	0.8315071723	−1.7520381069
39	H	−8.3823576855	4.0264772700	−2.4979799816
40	H	−9.5017315597	3.1460142302	−3.5701041571
41	H	−7.8539147352	2.5171672953	−3.3054669356
42	C	−8.4523375487	2.0152183718	−0.7104156983
43	H	−10.1129128983	0.9897675729	0.2121737173
44	H	−8.8147675253	0.3724941349	2.2545464446
45	H	−6.6556199649	2.9319080260	−1.4065922320
46	H	−4.6078336545	0.9959775822	3.7856183367
47	H	−3.3694726426	2.3277179410	2.0956040792
48	H	−2.4286876679	9.5566481028	0.4696516424
49	H	−3.5630102634	10.9539621927	0.8633025876
50	H	−5.3498035544	9.4598384666	1.4204969305
51	C	−5.2015673258	7.1119264162	−1.2751240141
52	C	−3.9881777134	6.1900432491	−1.5644225703
53	H	−4.5525164969	7.1979298841	2.0973491173
54	H	−2.4968684641	7.5667985055	−0.0766511915
55	H	−2.4305141956	6.5451446070	1.3552596024
56	H	−6.4772832696	7.4946394860	0.4454751827
57	H	−4.9628066837	8.1574262267	−1.5004914880
58	H	−6.0599553933	6.8321934384	−1.8954576054
59	H	−3.2145615761	6.7104672327	−2.1374496484
60	H	−4.2770277198	5.3155292585	−2.1561426424
61	H	−6.4049345618	5.0143793007	−0.1899418939
62	H	−6.1196352875	5.3015486338	1.5204052263
63	H	−3.8712680591	4.7248504489	1.4160760181
64	H	−4.7744501984	3.3887005342	−1.1710508234
65	H	−2.3379864416	3.9257315403	−0.3412334824
66	H	−0.8389441796	2.9638495148	1.1443931666
67	H	0.7635812754	3.2684318580	−2.8118146615
68	H	2.4917996726	5.5638077628	0.3949390776
**Q-TU_anion_4**				
E = −2412.33006873694				
1	C	−6.6860981489	1.2553246129	1.9363320868
2	O	−6.7104891568	2.3440769333	1.0278732797
3	C	−5.6830559311	3.6845653904	−0.5887233042
4	C	−4.3908030557	1.9318198347	0.4761894823

5	C	−3.2532587509	2.2834098877	−0.3041079074
6	C	−4.6054670887	4.0471285579	−1.3402923641
7	C	−3.3634370126	3.3631556660	−1.2254388850
8	C	−1.9995336702	1.6037902620	−0.2274286404
9	C	−0.9846757683	2.0582077417	−1.0397213241
10	C	−1.1965595814	3.1412024355	−1.9100553922
11	N	−2.3391018502	3.7851546620	−2.0162004085
12	C	−1.7263393285	0.4213493672	0.7063298760
13	C	−1.5020967822	−0.8314865997	−0.1956900170
14	C	−2.8206617605	−1.2164472736	−0.9305410555
15	C	−3.2243504431	−2.6342433712	−0.5049615415
16	C	−2.1247363821	−3.6195424177	−0.9770783185
17	C	−0.7868400304	−3.1122902185	−0.3714382497
18	C	−2.5126876934	−5.0445232249	−0.6731973610
19	C	−1.9714152318	−5.8891962099	0.2003758198
20	N	−0.9874893857	−1.9902397411	0.5591209204
21	N	−0.8196989664	0.8902633037	1.7564108999
22	C	0.4699401358	0.7641101816	1.7876280716
23	N	1.0939737089	−0.1622285847	0.9169477207
24	C	2.4126842715	−0.3434724449	0.5554764867
25	C	2.8129237819	−1.6376988815	0.1886081480
26	C	4.0933882622	−1.8835888506	−0.2888293843
27	C	5.0192013908	−0.8566303749	−0.4145959688
28	C	3.3483601771	0.6967305498	0.4267556388
29	C	4.6237149440	0.4281312763	−0.0496964817
30	C	4.4558186433	−3.2733115890	−0.7331114974
31	C	5.6282687652	1.5433226653	−0.1443198313
32	F	5.7784465580	−3.5020770066	−0.6464366617
33	F	4.1090595335	−3.4965613361	−2.0147269086
34	F	3.8404983127	−4.2167946205	0.0009168452
35	F	6.4656971616	1.3732194853	−1.1836451108
36	F	6.4003005777	1.6202705892	0.9569920549
37	F	5.0473024023	2.7454575486	−0.2979580546
38	S	1.4416317588	1.6377051004	2.9441665787
39	H	−7.6891994141	1.1970895797	2.3597003803
40	H	−5.9626553589	1.4212468157	2.7432408222
41	H	−6.4547662156	0.3141333198	1.4232648237
42	C	−5.5774275413	2.6109818991	0.3360636077
43	H	−6.6335530721	4.2003948413	−0.6750364942
44	H	−4.6593106932	4.8646737786	−2.0518593717
45	H	−4.3042852466	1.1313327223	1.1974833110
46	H	−0.3829721930	3.4863950176	−2.5449700085
47	H	−0.0055034101	1.5898407862	−1.0005696722
48	H	−2.3751428054	−6.8894844517	0.3281964671
49	H	−1.1135944607	−5.6418443909	0.8199948877
50	H	−3.3766309203	−5.3901377216	−1.2428815733
51	C	−3.3325563363	−2.6734196084	1.0264888489
52	C	−1.9237957229	−2.4045727005	1.6138326793
53	H	−2.0710971334	−3.5341216318	−2.0706124787
54	H	−0.2545982223	−3.9001764452	0.1690508659
55	H	−0.1143145766	−2.7681611801	−1.1638926785
56	H	−4.1837697808	−2.9016644108	−0.9600223296

57	H	−3.7129035265	−3.6424304017	1.3630997899
58	H	−4.0448629846	−1.9092084016	1.3599106077
59	H	−1.5101234784	−3.3090704053	2.0707501683
60	H	−1.9358470034	−1.6341723378	2.3899831592
61	H	−3.6257678024	−0.5156809868	−0.6820796476
62	H	−2.6848142117	−1.1634409711	−2.0161005944
63	H	−0.7271720566	−0.5955346095	−0.9323049293
64	H	−2.6546246331	0.2019958981	1.2446834396
65	H	2.1078540191	−2.4569484238	0.2934795124
66	H	3.0650845119	1.7008188392	0.7116899048
67	H	6.0176552938	−1.0469618865	−0.7879100657
68	H	0.5121406094	−1.0055358391	0.8206173263
**DQ-TU_anion_1**				
E = −2411.09802235152				
1	C	−8.6605194959	3.1153258909	−2.7648922209
2	O	−9.0748240596	2.2012771643	−1.7634452873
3	C	−8.6948695487	1.0432018211	0.2326133369
4	C	−6.9443014408	2.4694240218	−0.6489507957
5	C	−6.1073105094	2.1326812302	0.4513079007
6	C	−7.9076089719	0.6971865144	1.2906172808
7	C	−6.5953156546	1.2272426376	1.4345947561
8	C	−4.7925883297	2.6606352641	0.6295915707
9	C	−4.0839402676	2.2362824164	1.7307612545
10	C	−4.6587932689	1.3315304848	2.6416474546
11	N	−5.8711473340	0.8342933001	2.5186963725
12	C	−4.1674761454	3.6886624348	−0.3060975449
13	C	−4.2506997058	5.0843448468	0.3510538844
14	C	−5.6762361285	5.5026673470	0.8001987235
15	C	−5.8397139770	7.0068933749	0.5377385879
16	C	−4.6154821450	7.7391884648	1.1494269300
17	C	−3.3605270817	7.2862600935	0.3197219941
18	C	−4.7738506534	9.1922201175	1.1638592344
19	C	−4.8998899811	10.3919804211	1.1480590884
20	N	−3.6641306316	6.1247584809	−0.5080631627
21	N	−2.7797519908	3.3795368875	−0.5480054755
22	C	−2.4321908253	2.8569186871	−1.6732467832
23	N	−1.0566877205	2.6455553773	−1.8720977769
24	C	0.0438080010	3.0811245450	−1.1570735396
25	C	−0.0093094816	3.9270057389	−0.0393793832
26	C	1.1660155766	4.3237321407	0.5896158406
27	C	2.4151144823	3.9090765424	0.1497942963
28	C	1.3106004942	2.6630115493	−1.6062259167
29	C	2.4666562721	3.0738470674	−0.9638775653
30	C	1.0473772852	5.1972872316	1.8091549122
31	C	3.8015189294	2.5626129746	−1.4315761069
32	F	2.2250891054	5.7349986825	2.1689455312
33	F	0.5922443121	4.5074594094	2.8724459047
34	F	0.1900531817	6.2174489785	1.6206198427
35	F	4.1869747533	1.4714796503	−0.7421677375
36	F	4.7722857981	3.4795289025	−1.2711402619
37	F	3.7920453882	2.2167678637	−2.7298977215
38	S	−3.4149988486	2.3357855629	−3.0284363899

39	H	−8.4553838632	4.1052573839	−2.3391224223
40	H	−9.4943847271	3.1903515610	−3.4634996629
41	H	−7.7710652405	2.7565800318	−3.2963916867
42	C	−8.2076957942	1.9401527011	−0.7558937287
43	H	−9.6968495571	0.6431561570	0.1171317906
44	H	−8.2559873601	0.0104380917	2.0554617463
45	H	−6.5580902473	3.1246706052	−1.4172738371
46	H	−4.0880703065	1.0083461910	3.5103637295
47	H	−3.0697021436	2.5907274499	1.8839414506
48	H	−5.0092974319	11.4548053537	1.1423363092
49	C	−5.8523474059	7.2306586757	−0.9801563491
50	C	−4.5971477082	6.5303182821	−1.5658919227
51	H	−4.5007417254	7.3992585271	2.1868860467
52	H	−3.0313704141	8.0979316089	−0.3374559359
53	H	−2.5262261662	7.0422457461	0.9848349292
54	H	−6.7613980067	7.3799724900	0.9926798958
55	H	−5.8498745167	8.3041491418	−1.2011066649
56	H	−6.7694170058	6.8131500059	−1.4097551941
57	H	−4.0566272580	7.1916649431	−2.2513921887
58	H	−4.8636410046	5.6405040896	−2.1449285816
59	H	−6.4442472355	4.9588257789	0.2373535700
60	H	−5.8337056574	5.2614716882	1.8569293016
61	H	−3.5996216339	5.0312813801	1.2350641380
62	H	−4.7260241975	3.7002193262	−1.2492886579
63	H	−0.8512364395	2.1568139256	−2.7320901912
64	H	−0.9764873695	4.2642464301	0.3050027742
65	H	1.3778998552	2.0141162659	−2.4741218303
66	H	3.3206311792	4.2314720499	0.6475221451
**DQ-TU_anion_2**				
E = −2411.09288370535				
1	C	−8.6765651785	3.0963215366	−2.8397038290
2	O	−9.2308374298	2.3427705090	−1.7748729854
3	C	−9.0641960882	1.3452630788	0.3316112394
4	C	−7.1162732770	2.4470035586	−0.5995806744
5	C	−6.3643966768	2.1152356657	0.5618864073
6	C	−8.3589095899	0.9991162898	1.4453484042
7	C	−6.9934423013	1.3664333895	1.5950578519
8	C	−5.0001663604	2.4881555350	0.7570334672
9	C	−4.3893083062	2.0770044953	1.9182219053
10	C	−5.0992212099	1.3239804282	2.8690439680
11	N	−6.3600450048	0.9738917961	2.7353731665
12	C	−4.2418808034	3.4058853180	−0.1912261074
13	C	−4.2387844242	4.8334386977	0.4169166739
14	C	−5.6317576540	5.4992734688	0.5113987924
15	C	−5.4613519818	6.9897611773	0.1783167108
16	C	−4.3032326248	7.5523812597	1.0470283390
17	C	−2.9857189385	6.8529898371	0.5588772167
18	C	−4.2103620632	9.0102695792	0.9852747927
19	C	−4.1244450328	10.2108728523	0.9091777604
20	N	−3.2918715913	5.7078728745	−0.2945788154
21	N	−2.8476997327	3.0466726924	−0.3475499651
22	C	−2.3474445656	2.0518635583	−1.1642535473

23	N	−1.0645753509	2.0503225197	−1.4727467440
24	C	−0.1622148563	2.9374080301	−0.9152408291
25	C	−0.0857652423	3.2909264141	0.4479761122
26	C	0.8848382666	4.1710596487	0.9153108821
27	C	1.8355647940	4.7194861562	0.0663743429
28	C	0.8273716685	3.4863357930	−1.7600865909
29	C	1.7944577379	4.3544694448	−1.2792034308
30	C	0.8677305645	4.5253397664	2.3766212411
31	C	2.8563941106	4.8762581467	−2.2070305843
32	F	1.8039694639	5.4318123726	2.6997043908
33	F	1.0784090421	3.4468239284	3.1563259946
34	F	−0.3209061884	5.0362814402	2.7542747362
35	F	3.9522831491	4.0908390959	−2.2036512830
36	F	3.2692151515	6.1077318983	−1.8571278448
37	F	2.4371710735	4.9466788721	−3.4819279545
38	S	−3.4285244216	0.8475148400	−1.7826848358
39	H	−8.3474070809	4.0851846916	−2.4984983357
40	H	−9.4782918871	3.2178547006	−3.5689363984
41	H	−7.8362308827	2.5718778001	−3.3099666147
42	C	−8.4340956899	2.0738241794	−0.7121358458
43	H	−10.1064539057	1.0692711963	0.2109868330
44	H	−8.8146958539	0.4350712906	2.2525113888
45	H	−6.6263943768	2.9667266422	−1.4108642449
46	H	−4.6003064338	1.0029647253	3.7814728548
47	H	−3.3473161435	2.3241228552	2.0947372983
48	H	−4.0471526084	11.2747821930	0.8523260370
49	C	−5.0828873031	7.1141600964	−1.3035905407
50	C	−3.8656524231	6.1871426135	−1.5594992290
51	H	−4.4936515306	7.2699946609	2.0902238646
52	H	−2.3740275518	7.5528294018	−0.0181397405
53	H	−2.3848714562	6.5136363880	1.4074489550
54	H	−6.3827928246	7.5378476476	0.3908238495
55	H	−4.8442990192	8.1567503457	−1.5413348377
56	H	−5.9317690101	6.8241300611	−1.9316235256
57	H	−3.0747678239	6.7042689129	−2.1110118732
58	H	−4.1413367461	5.3129831786	−2.1574654541
59	H	−6.3358632907	5.0504133466	−0.1981329248
60	H	−6.0580389763	5.3574249578	1.5092725573
61	H	−3.8238750949	4.7229328491	1.4271213024
62	H	−4.7376218158	3.4109400581	−1.1687615259
63	H	−2.2859734726	3.8960190710	−0.3245916317
64	H	−0.8113759574	2.8823374752	1.1425093177
65	H	0.7961152652	3.2144943683	−2.8097151673
66	H	2.5803957658	5.4128683283	0.4346652577
**DQ-TU_anion_4**				
E = −2411.07390356070				
1	C	−6.6538251537	1.2705904129	1.9913592430
2	O	−6.7050408265	2.3291538682	1.0490141098
3	C	−5.7231380268	3.6210328381	−0.6338614646
4	C	−4.3933101664	1.9177218313	0.4647756380
5	C	−3.2748479477	2.2517586848	−0.3501492335
6	C	−4.6639658206	3.9672473000	−1.4184897036

7	C	−3.4133784793	3.2988209923	−1.3048171236
8	C	−2.0142310179	1.5843942350	−0.2777285875
9	C	−1.0200866262	2.0200976716	−1.1251657447
10	C	−1.2585806729	3.0728697881	−2.0251503036
11	N	−2.4088305155	3.7032715972	−2.1294512858
12	C	−1.7144097588	0.4333008157	0.6865492006
13	C	−1.4960474039	−0.8444761461	−0.1812968434
14	C	−2.8278416463	−1.2664446972	−0.8697913358
15	C	−3.2162418701	−2.6679659889	−0.3807017448
16	C	−2.1206774006	−3.6551481480	−0.8554287698
17	C	−0.7580223372	−3.1204009172	−0.2921157757
18	C	−2.3939998544	−5.0325347775	−0.4433675462
19	C	−2.6144531233	−6.1617131552	−0.0815273300
20	N	−0.9526402872	−1.9733090824	0.5986855926
21	N	−0.7952850719	0.9383604864	1.7078076675
22	C	0.4967483009	0.8336270125	1.7178686635
23	N	1.1191040231	−0.1006993552	0.8531711977
24	C	2.4359208863	−0.2815291517	0.4808918322
25	C	2.8321374358	−1.5821465728	0.1253406612
26	C	4.1057760788	−1.8355189535	−0.3603320109
27	C	5.0342852145	−0.8106043305	−0.5063317687
28	C	3.3718614826	0.7527809413	0.3333260028
29	C	4.6455323658	0.4758739305	−0.1505582947
30	C	4.4688991752	−3.2300874726	−0.7883499387
31	C	5.5983740516	1.6250032668	−0.3372627614
32	F	5.7831037373	−3.4761936549	−0.6388162194
33	F	4.1828728939	−3.4461171947	−2.0863671608
34	F	3.8066845551	−4.1664483568	−0.0876210557
35	F	5.1960782993	2.4471099896	−1.3260282226
36	F	6.8382065408	1.2146581991	−0.6538185459
37	F	5.7039195547	2.3821689746	0.7692400731
38	S	1.4747218614	1.7473372163	2.8354871314
39	H	−7.6483270670	1.2175069981	2.4351701074
40	H	−5.9172753348	1.4682655306	2.7790582896
41	H	−6.4239086109	0.3152450988	1.5045054213
42	C	−5.5887031068	2.5814439375	0.3252838497
43	H	−6.6802020256	4.1247399735	−0.7189640234
44	H	−4.7398625262	4.7589106872	−2.1567864014
45	H	−4.2851040063	1.1448257171	1.2127206114
46	H	−0.4607757877	3.4041434803	−2.6869351387
47	H	−0.0365352320	1.5607129247	−1.0923073090
48	H	−2.8086315355	−7.1646546829	0.2304506909
49	C	−3.2767828363	−2.6611794008	1.1534553325
50	C	−1.8553332677	−2.3583536834	1.6932219954
51	H	−2.0916674775	−3.6308136116	−1.9510630339
52	H	−0.2370588536	−3.9045743314	0.2644764590
53	H	−0.1060273402	−2.8068083803	−1.1131437608
54	H	−4.1822670304	−2.9617435726	−0.8000602040
55	H	−3.6395736183	−3.6237857174	1.5264755507
56	H	−3.9886537648	−1.8949560758	1.4817379509
57	H	−1.4203739153	−3.2414196809	2.1712045086
58	H	−1.8550457647	−1.5588050959	2.4389839252

59	H	−3.6325907935	−0.5629554287	−0.6293957080
60	H	−2.7191002770	−1.2547432737	−1.9594843849
61	H	−0.7417005168	−0.6253213969	−0.9437449408
62	H	−2.6318136071	0.2216286986	1.2461390532
63	H	2.1283686191	−2.3996328426	0.2499138409
64	H	3.0974581408	1.7600615878	0.6195080835
65	H	6.0312163467	−1.0098184139	−0.8772173192
66	H	0.5490787403	−0.9534411421	0.7879583117
**HQ-TU_cation_1**				
E = −2414.51843241094				
1	C	−10.1534046243	2.4300733486	−2.7434578107
2	O	−8.8248309617	2.7561524495	−2.3664923014
3	C	−9.0770102667	1.4017973569	−0.3476057150
4	C	−7.0426551943	2.6003802506	−0.8880365192
5	C	−6.4309092574	2.1295413312	0.2950614576
6	C	−8.4990113955	0.9270473932	0.8001034834
7	C	−7.1723122586	1.2673255655	1.1548909810
8	C	−5.0962707852	2.4524695973	0.6964842774
9	C	−4.6164049351	1.9087679188	1.8614970015
10	C	−5.4370593194	1.0592700359	2.6343465886
11	N	−6.6705746973	0.7452793393	2.3120106877
12	C	−4.2317383945	3.4150172093	−0.0943259935
13	C	−4.1937081421	4.8090736273	0.5843500894
14	C	−5.5089305456	5.6146514235	0.4588997083
15	C	−5.1141882536	7.0783514217	0.1791437595
16	C	−4.0857961573	7.4931192691	1.2598884118
17	C	−2.7840839728	6.6946703920	0.9823099066
18	C	−3.8450351006	8.9959655467	1.4730772983
19	C	−3.3751259547	9.8262946259	0.2776478600
20	N	−3.0445647149	5.5750950133	0.0704064690
21	N	−2.8283928410	3.0047977790	−0.1603236384
22	C	−2.2973676794	1.9852760682	−0.7909475333
23	N	−0.9784877666	1.9443614850	−1.0077987731
24	C	−0.1050497923	3.0239230686	−0.6739695319
25	C	0.7954480535	2.8714287579	0.3711258718
26	C	1.6415356954	3.9299799865	0.6947881008
27	C	1.5741898329	5.1271991067	−0.0028758471
28	C	−0.1796608582	4.2164355966	−1.3927685713
29	C	0.6578292313	5.2656241888	−1.0430085714
30	C	2.6555178543	3.7406224389	1.7919888709
31	C	0.5840240126	6.5840032577	−1.7664413831
32	F	3.1554597533	4.9106903099	2.2216945817
33	F	3.6894251737	2.9904850652	1.3716821695
34	F	2.1185636679	3.1133273061	2.8503664683
35	F	−0.1425774201	6.5062010575	−2.8904895764
36	F	1.8085541517	7.0265499784	−2.1018559470
37	F	0.0244837855	7.5377103031	−0.9956310769
38	S	−3.3519428366	0.6818364777	−1.3350738634
39	H	−10.3259442794	2.9390804928	−3.6918132194
40	H	−10.8813055952	2.7922378431	−2.0089493407
41	H	−10.2774295402	1.3516395546	−2.8922152263
42	C	−8.3367954414	2.2498751510	−1.2090930044

43	H	−10.0952523990	1.1198036515	−0.5871759114
44	H	−9.0456405518	0.2731553285	1.4712155283
45	H	−6.5230293126	3.2487650436	−1.5826151473
46	H	−5.0529175745	0.6310766287	3.5573201605
47	H	−3.6090004748	2.1241254866	2.2054059660
48	H	−4.1681721734	9.9639027646	−0.4637595400
49	H	−2.5099363774	9.3838481335	−0.2298358836
50	H	−3.0735482783	10.8256757059	0.6082767400
51	H	−3.1036598864	9.0954890193	2.2777190731
52	H	−4.7673602924	9.4427612585	1.8655201843
53	C	−4.5111262443	7.1050498387	−1.2362010423
54	C	−3.3322390457	6.1004534959	−1.2720683343
55	H	−4.4941558706	7.1365511956	2.2157435434
56	H	−2.0160292261	7.3195691538	0.5176195628
57	H	−2.3597410614	6.3031137110	1.9125336306
58	H	−5.9980196732	7.7232031365	0.2287798174
59	H	−4.1728682704	8.1053296229	−1.5098600758
60	H	−5.2821368397	6.8267888410	−1.9624992632
61	H	−2.4212094340	6.5720802976	−1.6473885896
62	H	−3.5477985682	5.2583790574	−1.9376630025
63	H	−6.1359586362	5.2401864040	−0.3580776668
64	H	−6.0988164072	5.5256585495	1.3756310488
65	H	−3.9766344832	4.6282213562	1.6435890779
66	H	−4.6014243557	3.5071007976	−1.1188725902
67	H	−2.2226195057	3.8084700715	0.0785028004
68	H	−0.5451497505	1.0657530341	−1.2860314766
69	H	0.8288032718	1.9421668597	0.9306258336
70	H	−0.8853384890	4.3176998173	−2.2098727218
71	H	2.2294148211	5.9490770806	0.2604934930
72	H	−2.4648441415	0.2037432693	−2.2250518140
		**HQ-TU_cation_2**		
		E = −2414.56410290504		
1	C	−9.7072411186	1.3167225087	−1.4475024247
2	O	−8.4893051566	2.0441982850	−1.4442952425
3	C	−7.8830285411	0.9925325330	0.6768878965
4	C	−6.4671042606	2.6026893624	−0.4503073206
5	C	−5.5044604787	2.5017828103	0.5790974820
6	C	−6.9583206130	0.8728606473	1.6805279259
7	C	−5.7542218306	1.6151102869	1.6674303627
8	C	−4.2832769273	3.2457354443	0.6128865931
9	C	−3.4416342837	3.0537249660	1.6803568050
10	C	−3.7772968289	2.1327816931	2.6962910791
11	N	−4.8881618699	1.4345977306	2.7072450488
12	C	−3.9141001062	4.2452787363	−0.4774783561
13	C	−4.0640801457	5.6767380930	0.0547516882
14	C	−5.4734322706	6.0259596373	0.5714840093
15	C	−5.7819123645	7.4835527058	0.1851884698
16	C	−4.5618580387	8.3360052419	0.6028608090
17	C	−3.4165859984	8.0079970344	−0.3727160558
18	C	−4.7772155602	9.8481382802	0.7754733574
19	C	−5.3736391154	10.6278079507	−0.3964252121
20	N	−3.7103023161	6.6793012202	−1.0152196735

21	N	−2.5552478340	4.0465857278	−0.9498090823
22	C	−2.1998094852	3.3861326592	−2.0769320506
23	N	−0.9002568739	2.9863899127	−2.1255313981
24	C	0.0054335025	2.9266677702	−1.0383581194
25	C	−0.3423678222	2.2499401536	0.1335222698
26	C	0.5483909260	2.2216361427	1.1987946422
27	C	1.8017854531	2.8183844527	1.0966252497
28	C	1.2548470246	3.5309115042	−1.1494047719
29	C	2.1502004932	3.4586576651	−0.0856109708
30	C	0.1281532184	1.5844101501	2.4958618031
31	C	3.5195660601	4.0637737207	−0.2455393737
32	F	1.1763051254	1.0868339996	3.1733917478
33	F	−0.7477379688	0.5853494357	2.3039488292
34	F	−0.4719621684	2.4802572138	3.3063074187
35	F	4.3260328465	3.2574129120	−0.9613132889
36	F	4.1169910453	4.2768772680	0.9388306939
37	F	3.4671816865	5.2389098535	−0.8910301140
38	S	−3.2442105432	3.0864166385	−3.3789605240
39	H	−9.5313455774	0.2355711927	−1.4770765412
40	H	−10.2283439736	1.6135964322	−2.3577711686
41	H	−10.3306289394	1.5702656624	−0.5827591923
42	C	−7.6341264118	1.8700004092	−0.4082445120
43	H	−8.7946565218	0.4087478238	0.7205651803
44	H	−7.1257579247	0.2035852323	2.5176587724
45	H	−6.3175493567	3.2388453156	−1.3142238011
46	H	−3.0913362519	1.9732427881	3.5241280364
47	H	−2.5018266255	3.5900007091	1.7774582118
48	H	−6.4194799636	10.3613635459	−0.5777226455
49	H	−4.8199727613	10.4849090015	−1.3312642379
50	H	−5.3529521531	11.7009106349	−0.1814832124
51	H	−3.8098051818	10.2947470482	1.0395131381
52	H	−5.4185497682	9.9961666479	1.6522097456
53	C	−6.0047036339	7.5072895002	−1.3373581624
54	C	−4.8036604901	6.8400063978	−2.0287822717
55	H	−4.2608317381	7.9803997607	1.5955974524
56	H	−3.3287748307	8.7325319996	−1.1848567323
57	H	−2.4513213988	7.9284387194	0.1293681801
58	H	−6.6813209048	7.8271987107	0.7026935501
59	H	−6.1340812306	8.5244227270	−1.7063149839
60	H	−6.9179490559	6.9596000643	−1.5833745045
61	H	−4.3917554660	7.4430920478	−2.8381630225
62	H	−5.0275436202	5.8526514547	−2.4326435072
63	H	−6.2201330001	5.3540307533	0.1358882917
64	H	−5.5031515908	5.8796743833	1.6531549950
65	H	−3.3218358128	5.8320247609	0.8443241594
66	H	−4.5570527812	4.0996154005	−1.3435868326
67	H	−1.8230201814	4.2420723314	−0.2714672142
68	H	−0.5612697630	2.6926728477	−3.0333910458
69	H	−1.3034243034	1.7528487319	0.2080384931
70	H	1.5183787308	4.0609793112	−2.0588233885
71	H	2.4960113561	2.7833320587	1.9270234897
72	H	−2.8615997352	6.3528222476	−1.4966069213

**HQ-TU_cation_3**				
E = −2414.53452577752				
1	C	−5.8402762237	4.9739269358	1.1362140268
2	O	−5.0566464690	3.7937451513	1.2143527296
3	C	−4.5817875600	3.9628006803	−1.1768771330
4	C	−3.7209694931	2.1792359192	0.2160070997
5	C	−3.0609363186	1.6037218903	−0.8914769455
6	C	−3.9499698026	3.4203374941	−2.2647801569
7	C	−3.1774541449	2.2403599075	−2.1595299057
8	C	−2.2688453929	0.4105668340	−0.8225457992
9	C	−1.6747295827	−0.0290607859	−1.9766990527
10	C	−1.8606684677	0.6786162260	−3.1861256295
11	N	−2.5778437347	1.7712977613	−3.2940307115
12	C	−2.0809998851	−0.2850040345	0.5196285500
13	C	−1.7206986030	−1.7719890347	0.3894381002
14	C	−2.8240184533	−2.6397387413	−0.2459363698
15	C	−2.8492282736	−3.9972883146	0.4813123046
16	C	−1.3886624909	−4.5012972051	0.5530989330
17	C	−0.6500542948	−3.6262351015	1.5834969638
18	C	−1.1669811567	−6.0045604655	0.7852363895
19	C	−1.8195217828	−6.6481850538	2.0087436502
20	N	−1.4201527372	−2.3483308979	1.7488916575
21	N	−1.1407141489	0.4395954702	1.3815407779
22	C	0.1473243030	0.7115837571	1.0342568273
23	N	0.7394258427	0.0186593701	0.1383430910
24	C	2.0292837010	0.2819272512	−0.3095716578
25	C	2.2488824842	1.1747262893	−1.3640995747
26	C	3.5374120242	1.3919845351	−1.8339081679
27	C	4.6275531020	0.7325829168	−1.2729062547
28	C	3.1190499394	−0.3891471669	0.2487657743
29	C	4.4047361346	−0.1580925987	−0.2318574393
30	C	3.7521351593	2.4065138712	−2.9245928550
31	C	5.5496048202	−0.8810876694	0.4246267598
32	F	4.9098112651	2.2065418530	−3.5778943195
33	F	3.7886153766	3.6571779986	−2.4286648830
34	F	2.7644095953	2.3752057108	−3.8339311937
35	F	5.7083421595	−0.4839782972	1.7002674678
36	F	6.7164546732	−0.6712720626	−0.2081089938
37	F	5.3402730407	−2.2075364975	0.4554562577
38	S	0.8451295362	2.0956969148	1.9591332144
39	H	−5.2396252000	5.8367317250	0.8273211160
40	H	−6.2163937093	5.1480747731	2.1443407699
41	H	−6.6905814138	4.8485058767	0.4568112654
42	C	−4.4666837986	3.3320180539	0.0865850925
43	H	−5.1641016480	4.8687568707	−1.2948131971
44	H	−4.0247314110	3.8841631181	−3.2426078385
45	H	−3.6743361075	1.7407856559	1.2053413085
46	H	−1.3865542470	0.3188110472	−4.0966964253
47	H	−1.0424404427	−0.9081103030	−1.9958881412
48	H	−1.5663750340	−6.1386733228	2.9453273441
49	H	−1.4788333229	−7.6831015004	2.1135439098
50	H	−2.9100482170	−6.6812199073	1.9233174756

51	H	−0.0838235082	−6.1779775985	0.8314389059
52	H	−1.5111759485	−6.5349472255	−0.1104521904
53	C	−3.4393766679	−3.7509283893	1.8800428228
54	C	−2.6623355997	−2.6062593263	2.5512188786
55	H	−0.9420326160	−4.2993460718	−0.4279171656
56	H	−0.5943674692	−4.0896204079	2.5706524597
57	H	0.3584603239	−3.3633488785	1.2613966738
58	H	−3.4687665357	−4.7097290322	−0.0697802818
59	H	−3.3952467351	−4.6488805762	2.4956806716
60	H	−4.4935759271	−3.4771146680	1.7906018966
61	H	−2.3383256819	−2.8495956198	3.5634892418
62	H	−3.2283426880	−1.6760609473	2.5943673106
63	H	−3.7995873800	−2.1472564806	−0.1641817996
64	H	−2.6175737077	−2.7614717378	−1.3113605039
65	H	−0.7784755228	−1.8598503223	−0.1505631102
66	H	−3.0377628394	−0.2286984858	1.0444876451
67	H	1.4050422500	1.6876813308	−1.8127068599
68	H	2.9538758858	−1.0945312798	1.0570108449
69	H	5.6282365667	0.9039822681	−1.6476881353
70	H	−0.8413816092	−1.6698789164	2.2611881282
71	H	−1.5572408425	0.9866958933	2.1246684452
72	H	2.1202786633	1.7691485741	1.7103949105
		**Q-TU_cation_1**		
		E = −2413.28010809340		
1	C	−10.1156152959	2.2998917865	−2.7278859966
2	O	−8.7824207285	2.6283530362	−2.3698229633
3	C	−9.0627040331	1.4220678286	−0.2626774838
4	C	−7.0081350062	2.5487010936	−0.8760167154
5	C	−6.4086336517	2.1552298966	0.3410817094
6	C	−8.4963335398	1.0207905805	0.9183002742
7	C	−7.1663882457	1.3661902851	1.2554910547
8	C	−5.0720432521	2.4885013730	0.7273384131
9	C	−4.6062167411	2.0218764622	1.9309865739
10	C	−5.4418336446	1.2386594957	2.7558737188
11	N	−6.6779628216	0.9194396377	2.4490590985
12	C	−4.1881450699	3.3798343972	−0.1241825073
13	C	−4.1425426002	4.8220823530	0.4469688785
14	C	−5.4370170187	5.6354491387	0.2229851628
15	C	−5.0188250854	7.0614674709	−0.1762677093
16	C	−4.0018034197	7.5869960067	0.8708713836
17	C	−2.7164479233	6.7112586984	0.7372253310
18	C	−3.6955833436	9.0492276302	0.7287039220
19	C	−3.9563887067	9.9731798565	1.6484085374
20	N	−2.9695568754	5.5316476293	−0.0922944795
21	N	−2.7901005701	2.9492883316	−0.1392437805
22	C	−2.2645329074	1.8795578661	−0.6866823361
23	N	−0.9426310302	1.8059489899	−0.8716509489
24	C	−0.0705643781	2.9002901305	−0.5816300271
25	C	0.7344090956	2.8511159058	0.5472043450
26	C	1.5627400019	3.9347788180	0.8349435388
27	C	1.5692174502	5.0548993071	0.0177161868
28	C	−0.0676643107	4.0131841780	−1.4221365026

29	C	0.7496477292	5.0886887744	−1.1091296556
30	C	2.4195230011	3.8779341641	2.0721065919
31	C	0.7563607489	6.3271673348	−1.9647785075
32	F	3.2527260205	4.9275297621	2.1581035015
33	F	3.1671354784	2.7612491359	2.0927281254
34	F	1.6656075511	3.8662968764	3.1836296223
35	F	0.0825916539	6.1567023735	−3.1109940876
36	F	2.0099981865	6.6982171723	−2.2787390881
37	F	0.1920870535	7.3688803145	−1.3220543210
38	S	−3.3309666397	0.5591863967	−1.1602226064
39	H	−10.2784903476	2.7486677774	−3.7078334903
40	H	−10.8386557010	2.7207362430	−2.0203040485
41	H	−10.2568184212	1.2161184950	−2.8059654642
42	C	−8.3064863042	2.1965042997	−1.1778644814
43	H	−10.0840735269	1.1388371860	−0.4868918748
44	H	−9.0550229056	0.4232631601	1.6307962657
45	H	−6.4752066192	3.1368476398	−1.6128630657
46	H	−5.0679151798	0.8700178304	3.7083351730
47	H	−3.5979905248	2.2475704883	2.2657628674
48	H	−4.4295787452	9.7233521863	2.5958730385
49	H	−3.7093208129	11.0195846648	1.4945702155
50	H	−3.2113223973	9.3573379082	−0.1993154871
51	C	−4.3607888508	6.9852976048	−1.5615932691
52	C	−3.2061311830	5.9538341815	−1.4816979660
53	H	−4.4416312155	7.4337667674	1.8649119559
54	H	−1.9031076495	7.2738544053	0.2687686777
55	H	−2.3621032812	6.3887340117	1.7205381536
56	H	−5.8933929900	7.7176001680	−0.2015867719
57	H	−3.9885949349	7.9671909604	−1.8703617470
58	H	−5.1031992935	6.6850524025	−2.3077952179
59	H	−2.2728833964	6.3718773300	−1.8667592093
60	H	−3.4264829492	5.0663047960	−2.0831028707
61	H	−6.0538421506	5.2041206798	−0.5729910259
62	H	−6.0497989864	5.6388985513	1.1289664017
63	H	−3.9554619825	4.7205187094	1.5221649168
64	H	−4.5446942240	3.3971506878	−1.1575064357
65	H	−2.1762729205	3.7589432131	0.0537722150
66	H	−0.5141000971	0.9088191987	−1.0918347898
67	H	0.7098422123	1.9816203416	1.1963764867
68	H	−0.7009276004	4.0312886927	−2.3021689750
69	H	2.2090277647	5.8971230725	0.2528028699
70	H	−2.4282419176	−0.0085151420	−1.9788362998

		**Q-TU_cation_2**		
		E = −2413.32667716321		
1	C	−8.5846932088	2.5401090039	−2.2078762010
2	O	−8.6853699442	1.6278569048	−1.1252619820
3	C	−7.8445195545	0.6987150234	0.8491767776
4	C	−6.5378199141	2.3801858251	−0.3095799335
5	C	−5.5309183600	2.2897585050	0.6896606590
6	C	−6.8872863859	0.5874842664	1.8128100821
7	C	−5.7061806577	1.3784647130	1.7678723995
8	C	−4.3413139405	3.0763083743	0.6882030000

9	C	−3.4516513588	2.9041513438	1.7232436126
10	C	−3.7136858719	1.9578084059	2.7320641913
11	N	−4.7987133448	1.2154262486	2.7699380055
12	C	−4.0503486894	4.0995216472	−0.4039841849
13	C	−4.2508230111	5.5212733343	0.1372592924
14	C	−5.6589522035	5.8052382558	0.6898516772
15	C	−6.0623710787	7.2320112444	0.2907500269
16	C	−4.8989722640	8.1963224322	0.6397453805
17	C	−3.7275515974	7.8796394351	−0.3090927219
18	C	−5.3473209860	9.6343355360	0.6349901315
19	C	−4.9122539904	10.6360667900	−0.1229716174
20	N	−3.9757264571	6.5388152754	−0.9392634427
21	N	−2.7008544560	3.9645034121	−0.9228374167
22	C	−2.3581362981	3.3126147342	−2.0590982627
23	N	−1.0432712788	2.9784286413	−2.1595661691
24	C	−0.0883066691	2.9875326841	−1.1139625995
25	C	−0.3436796412	2.3100507674	0.0806841924
26	C	0.5928446953	2.3512836602	1.1058989230
27	C	1.8031279648	3.0178035338	0.9402114984
28	C	1.1182544489	3.6605385734	−1.2889475968
29	C	2.0620118707	3.6567779209	−0.2658204763
30	C	0.2673457449	1.7037628896	2.4251973560
31	C	3.3885896473	4.3310355877	−0.4959190598
32	F	1.3739863684	1.3341434779	3.0909511792
33	F	−0.5020915872	0.6142449245	2.2712034310
34	F	−0.4099528166	2.5488673432	3.2295262426
35	F	4.2069943373	3.5524409101	−1.2287827842
36	F	4.0225324142	4.6027102255	0.6570281807
37	F	3.2458147813	5.4866585164	−1.1625897724
38	S	−3.4362129149	2.9467871161	−3.3159732930
39	H	−8.5329023223	3.5746905493	−1.8482305819
40	H	−9.4950648365	2.4137188092	−2.7935619498
41	H	−7.7174402935	2.3167481070	−2.8402316204
42	C	−7.6711112944	1.6068234654	−0.2299140766
43	H	−8.7493556346	0.1013776892	0.8792914195
44	H	−6.9984101003	−0.1020395147	2.6427164947
45	H	−6.4062787164	3.0432391108	−1.1529452383
46	H	−2.9940396998	1.8115016785	3.5334003108
47	H	−2.5305791801	3.4757858968	1.7940300626
48	H	−4.1222183282	10.5410290571	−0.8619028195
49	H	−5.3320895472	11.6320056867	−0.0211396343
50	H	−6.1357925436	9.8445528038	1.3574433476
51	C	−6.3105476617	7.2699969343	−1.2226321612
52	C	−5.0975572222	6.6461683000	−1.9330217522
53	H	−4.5897230657	7.9699421903	1.6682023927
54	H	−3.6436140724	8.5846102865	−1.1363674052
55	H	−2.7694113079	7.8334270884	0.2091600041
56	H	−6.9651798993	7.5277749751	0.8301064473
57	H	−6.4621327890	8.3014868065	−1.5516848332
58	H	−7.2136804595	6.7095168406	−1.4755303669
59	H	−4.7271610295	7.2566166156	−2.7567106071
60	H	−5.2886585701	5.6460712145	−2.3219076996

61	H	−6.3836311476	5.0896034150	0.2882509664
62	H	−5.6529562771	5.6777167941	1.7741669752
63	H	−3.4956925733	5.7112533688	0.9069638461
64	H	−4.7108786944	3.9297657857	−1.2521101769
65	H	−1.9540892105	4.1996947430	−0.2736546740
66	H	−0.7286343057	2.6906668844	−3.0780128176
67	H	−1.2682358173	1.7561325451	0.2033575125
68	H	1.3109680128	4.1890988226	−2.2168751459
69	H	2.5342300017	3.0352498980	1.7386405382
70	H	−3.1224022071	6.2555596694	−1.4402504827
**Q-TU_cation_3**				
E = −2413.29487092283				
1	C	−5.8590536579	5.0024494583	0.9747647834
2	O	−5.0896116378	3.8158042091	1.0877583175
3	C	−4.5685861670	3.9378188557	−1.2966073797
4	C	−3.7501376544	2.1719766489	0.1442778035
5	C	−3.0748411834	1.5706564852	−0.9400927082
6	C	−3.9215343480	3.3698903664	−2.3625193849
7	C	−3.1626901917	2.1849058634	−2.2214144074
8	C	−2.2935352149	0.3728659947	−0.8348549344
9	C	−1.6814539238	−0.0917587108	−1.9698175149
10	C	−1.8397862036	0.5947693606	−3.1952566959
11	N	−2.5467141405	1.6902688593	−3.3362639018
12	C	−2.1361513882	−0.2996422790	0.5228305812
13	C	−1.7728024467	−1.7887149754	0.4264115381
14	C	−2.8592758322	−2.6684337395	−0.2152424840
15	C	−2.9168400109	−4.0036361210	0.5428630495
16	C	−1.4722433595	−4.5470097541	0.7005813728
17	C	−0.7259752294	−3.6176068688	1.6775515388
18	C	−1.4622675251	−6.0039502387	1.0820181976
19	C	−0.9092749837	−6.5770740848	2.1464441074
20	N	−1.5041553422	−2.3422200329	1.8002335997
21	N	−1.2147063977	0.4392185300	1.3926754693
22	C	0.0787967260	0.7102976678	1.0663748350
23	N	0.6905572953	0.0053267810	0.1933312943
24	C	1.9853520309	0.2730048466	−0.2378179489
25	C	2.2151626441	1.1593173956	−1.2954902207
26	C	3.5094990000	1.3827496830	−1.7464434131
27	C	4.5950734793	0.7345171717	−1.1643055642
28	C	3.0708188395	−0.3873176521	0.3416669250
29	C	4.3621260476	−0.1507270532	−0.1206338646
30	C	3.7319919657	2.3892932472	−2.8429506526
31	C	5.5019535446	−0.8618485002	0.5572287277
32	F	4.9115282343	2.2084764105	−3.4617775999
33	F	3.7258992779	3.6460485722	−2.3615440184
34	F	2.7707721106	2.3246336748	−3.7786727634
35	F	5.6470325272	−0.4466363308	1.8287594436
36	F	6.6745721721	−0.6577636830	−0.0666963195
37	F	5.2954629746	−2.1882131292	0.6042623671
38	S	0.7522893674	2.1144723769	1.9785653444
39	H	−5.2431791340	5.8536646245	0.6637240465
40	H	−6.2530367382	5.1970841695	1.9722553778

41	H	−6.6970893025	4.8745962705	0.2807150938
42	C	−4.4828398464	3.3288499818	−0.0201779325
43	H	−5.1404374200	4.8463547242	−1.4423284666
44	H	−3.9738261757	3.8164121277	−3.3498026187
45	H	−3.7264922013	1.7508051510	1.1419338019
46	H	−1.3515323997	0.2149947521	−4.0901049832
47	H	−1.0555860926	−0.9754481163	−1.9628427620
48	H	−0.3513706953	−6.0378658953	2.9062877139
49	H	−0.9785700361	−7.6498719547	2.2974806894
50	H	−1.9827314865	−6.6435000156	0.3692537579
51	C	−3.5148113071	−3.7603058183	1.9339281000
52	C	−2.7620671731	−2.5891433198	2.5861917748
53	H	−0.9911303218	−4.4789452251	−0.2838164720
54	H	−0.6586010193	−4.0270166261	2.6858967342
55	H	0.2761022077	−3.3613282664	1.3329588114
56	H	−3.5248646556	−4.7227662690	−0.0110911318
57	H	−3.4265579695	−4.6639632032	2.5430040984
58	H	−4.5776283068	−3.5200845859	1.8570618771
59	H	−2.4570787023	−2.7977552347	3.6119006590
60	H	−3.3376246760	−1.6638640229	2.5870780407
61	H	−3.8354404652	−2.1726423675	−0.1744238661
62	H	−2.6251701061	−2.8220887623	−1.2706803692
63	H	−0.8184739813	−1.8858961748	−0.0906032012
64	H	−3.1044980946	−0.2341665254	1.0251476388
65	H	1.3750282361	1.6627072613	−1.7617159594
66	H	2.8978439629	−1.0879628894	1.1523701784
67	H	5.6002697830	0.9103140705	−1.5246586275
68	H	−0.9350459257	−1.6557293056	2.3131810497
69	H	−1.6466078272	0.9924825244	2.1223661891
70	H	2.0335914715	1.7821216153	1.7712382379
**DQ-TU_cation_1**				
E = −2412.02359077219				
1	C	−10.0660268563	2.3277919026	−2.8198394911
2	O	−8.7374990416	2.6450675535	−2.4352816862
3	C	−9.0863302458	1.4841976620	−0.3129114006
4	C	−7.0031750365	2.5751975711	−0.8947517655
5	C	−6.4404188440	2.2004461397	0.3456692109
6	C	−8.5559647131	1.1017020506	0.8908043895
7	C	−7.2316181934	1.4400307625	1.2561146765
8	C	−5.1098832452	2.5248200205	0.7596560343
9	C	−4.6823888423	2.0803226825	1.9856893132
10	C	−5.5492804391	1.3258731613	2.8050952529
11	N	−6.7803278856	1.0137174811	2.4716905558
12	C	−4.1891981278	3.3816804086	−0.0891937811
13	C	−4.1311837057	4.8334159632	0.4526387738
14	C	−5.4135228479	5.6557603063	0.1932078435
15	C	−4.9794540885	7.0611802146	−0.2525459966
16	C	−3.9654379723	7.6152540146	0.7858132441
17	C	−2.6860570182	6.7092537803	0.7216127796
18	C	−3.6476265016	9.0232987411	0.5571458391
19	C	−3.3670941414	10.1758300829	0.3432006125
20	N	−2.9430657173	5.5196793726	−0.0843771054

21	N	−2.8008026637	2.9200819618	−0.0621938309
22	C	−2.2921483278	1.8213859213	−0.5672600140
23	N	−0.9692284404	1.7015658958	−0.7154497003
24	C	−0.0630165052	2.7719694817	−0.4448806566
25	C	0.7671761506	2.6979409599	0.6651126531
26	C	1.6427775842	3.7499294776	0.9255739366
27	C	1.6706032886	4.8659419325	0.1012908088
28	C	−0.0389250436	3.8790449909	−1.2917108256
29	C	0.8252662242	4.9253592303	−1.0043235608
30	C	2.5135639345	3.6888104504	2.1531970016
31	C	0.8530130827	6.1584527728	−1.8675229189
32	F	3.5351349390	4.5589413146	2.0869830095
33	F	3.0334615314	2.4619551175	2.3224587735
34	F	1.8116189490	3.9763565716	3.2619771985
35	F	0.1834364351	5.9883640515	−3.0164726819
36	F	2.1131658326	6.5107922339	−2.1772158055
37	F	0.2985362642	7.2109182708	−1.2349978911
38	S	−3.3841663414	0.5198184153	−1.0357410927
39	H	−10.7999329729	2.7686067177	−2.1360814607
40	H	−10.2192436577	1.2447754044	−2.8844641499
41	H	−10.2002145290	2.7631027294	−3.8101603717
42	C	−8.2975380835	2.2317021954	−1.2231003556
43	H	−10.1044321183	1.2069982408	−0.5584857084
44	H	−9.1400134913	0.5252893846	1.6003315476
45	H	−6.4448697012	3.1418781217	−1.6296330939
46	H	−5.2054138811	0.9742845143	3.7751231875
47	H	−3.6802880457	2.2999648408	2.3422403410
48	H	−3.1262598979	11.2011409763	0.1658643352
49	C	−4.2910743168	6.9483466966	−1.6183571645
50	C	−3.1479345880	5.9091786497	−1.4886864378
51	H	−4.4187177245	7.5317511769	1.7813553084
52	H	−1.8509800340	7.2496614828	0.2707553253
53	H	−2.3825391238	6.4040595087	1.7264578080
54	H	−5.8451192566	7.7253636343	−0.3102315481
55	H	−3.9026285186	7.9252645997	−1.9232027675
56	H	−5.0177477508	6.6404389315	−2.3763745202
57	H	−2.2037995194	6.3104899109	−1.8645203458
58	H	−3.3637278172	5.0082841679	−2.0711762351
59	H	−6.0301390558	5.2087315238	−0.5936364782
60	H	−6.0335170967	5.6961188685	1.0931992294
61	H	−3.9606997561	4.7517485035	1.5322232194
62	H	−4.5207646491	3.3831382063	−1.1307954661
63	H	−2.1664515872	3.7091904882	0.1379716524
64	H	−0.5667952266	0.7837619341	−0.8961441389
65	H	0.7274535034	1.8320706565	1.3181533656
66	H	−0.6885193169	3.9154079265	−2.1592486291
67	H	2.3497279711	5.6835292909	0.3121997717
68	H	−2.4829881881	−0.0811372050	−1.8320359222
**DQ-TU_cation_2**				
E = −2412.06416576519				
1	C	−3.5853053202	4.7330903696	2.5831371265
2	O	−3.2277932038	3.4085896598	2.2254829259

3	C	−3.0416779498	4.1033524383	−0.1088569420
4	C	−2.5854981952	1.8408892344	0.6365586581
5	C	−2.2782334691	1.4529065510	−0.6867309285
6	C	−2.7507843791	3.7423892501	−1.3978758456
7	C	−2.3582868985	2.4242379077	−1.7248306227
8	C	−1.8516963921	0.1333777977	−1.0523180440
9	C	−1.5222297607	−0.0889007154	−2.3644732570
10	C	−1.6541871081	0.9479114019	−3.3177485227
11	N	−2.0628892572	2.1580485399	−3.0305219418
12	C	−1.6757707737	−0.9303749585	0.0258201556
13	C	−1.7028452081	−2.3598206592	−0.5343192684
14	C	−2.9892132427	−2.7513133910	−1.2836038147
15	C	−3.3084225792	−4.2248437712	−0.9887015924
16	C	−2.0044251590	−5.0472246989	−1.1679834182
17	C	−1.0370852041	−4.6587366146	−0.0184944388
18	C	−2.2446129245	−6.4890617748	−1.1761882377
19	C	−2.4511665657	−7.6749165486	−1.1551325962
20	N	−1.5184297114	−3.3672153942	0.5756448409
21	N	−0.4082070736	−0.6751263856	0.7055738592
22	C	−0.2259791238	−0.3808255709	2.0130978490
23	N	0.9395783296	0.2546418266	2.3050760415
24	C	1.7802082512	0.9321472597	1.3892072182
25	C	1.2477817999	1.8802147420	0.5124192086
26	C	2.0878640647	2.5344524024	−0.3784079204
27	C	3.4563378639	2.2817137053	−0.3861779187
28	C	3.1463525083	0.6668390331	1.3869285597
29	C	3.9771710270	1.3544645324	0.5066437059
30	C	1.4922960899	3.5003815824	−1.3682751416
31	C	5.4575351148	1.0873410241	0.5556280626
32	F	2.3999245320	4.3981723106	−1.7971123460
33	F	0.4679209594	4.1835272412	−0.8347519453
34	F	1.0244290545	2.8624937256	−2.4520255480
35	F	6.0124278377	1.6245340422	1.6583103286
36	F	6.1003408467	1.6005763971	−0.5066528316
37	F	5.7184548881	−0.2292609465	0.5897525598
38	S	−1.3173560977	−0.7815879046	3.2484563626
39	H	−2.7849690459	5.4446460307	2.3504024142
40	H	−3.7408338960	4.7175507359	3.6620279828
41	H	−4.5154322055	5.0466260479	2.0959595464
42	C	−2.9568461889	3.1390132498	0.9257261421
43	H	−3.3317286594	5.1245441186	0.1069394234
44	H	−2.8034825038	4.4646080988	−2.2054935016
45	H	−2.5350955111	1.1447350262	1.4673096175
46	H	−1.4085518229	0.7526134809	−4.3592582829
47	H	−1.1593573761	−1.0474155384	−2.7176304187
48	H	−2.6314935869	−8.7276666569	−1.1502814625
49	C	−3.7764791794	−4.3525916249	0.4643838694
50	C	−2.7772834204	−3.6069987713	1.3620159966
51	H	−1.5531161267	−4.7771492890	−2.1290840049
52	H	−1.0346807372	−5.3988576089	0.7816984859
53	H	−0.0159398350	−4.5093116959	−0.3695595317
54	H	−4.0721862345	−4.5932638685	−1.6748904773

55	H	−3.8468776355	−5.4069196983	0.7456002021
56	H	−4.7706040454	−3.9164731484	0.5847302697
57	H	−2.4889620302	−4.1721948863	2.2482212450
58	H	−3.1400421796	−2.6364289614	1.6957025717
59	H	−3.8256611520	−2.1193409478	−0.9668032564
60	H	−2.8553569544	−2.5879472153	−2.3540516776
61	H	−0.8247353106	−2.5006229443	−1.1703617422
62	H	−2.4696091129	−0.8258638031	0.7680749552
63	H	0.3665452010	−0.4892653095	0.0730228020
64	H	1.1940696584	0.2852468387	3.2846418724
65	H	0.1862949966	2.1075898051	0.5357193141
66	H	3.5533704268	−0.0760149426	2.0652944154
67	H	4.1066895502	2.8043280820	−1.0759743897
68	H	−0.7916182949	−3.0208480426	1.2134011999
**DQ-TU_cation_3**				
E = −2412.03717845262				
1	C	−5.8911799066	4.9746635406	0.9406327243
2	O	−5.1102450297	3.7966515352	1.0644876579
3	C	−4.5755078081	3.9105313865	−1.3174912053
4	C	−3.7499987889	2.1604098300	0.1383930179
5	C	−3.0620394313	1.5596320680	−0.9382307815
6	C	−3.9168001858	3.3425832039	−2.3760739510
7	C	−3.1473051029	2.1659937987	−2.2233585102
8	C	−2.2696173652	0.3704202938	−0.8214772731
9	C	−1.6454225447	−0.0942427306	−1.9497439730
10	C	−1.8023986097	0.5840004605	−3.1798843274
11	N	−2.5186022326	1.6720106794	−3.3312746374
12	C	−2.1121447660	−0.2914262941	0.5413844740
13	C	−1.7383153702	−1.7782926686	0.4546440184
14	C	−2.8192000938	−2.6668716879	−0.1848480765
15	C	−2.9005142561	−3.9876058324	0.5942007780
16	C	−1.4595966342	−4.5412720926	0.7585673079
17	C	−0.6856377218	−3.5918908343	1.7126561638
18	C	−1.4399704317	−5.9129975521	1.2628959043
19	C	−1.4371988694	−7.0323116503	1.7065494176
20	N	−1.4653752233	−2.3210086445	1.8326956639
21	N	−1.1971849315	0.4590725544	1.4080787131
22	C	0.0938296089	0.7378203316	1.0772788621
23	N	0.7111816923	0.0232266899	0.2161636713
24	C	1.9998052865	0.2972818252	−0.2279010110
25	C	2.2139964327	1.1793697213	−1.2924818079
26	C	3.5022399785	1.4067826502	−1.7578382216
27	C	4.5971891625	0.7682053378	−1.1824551430
28	C	3.0942662695	−0.3528792744	0.3453221365
29	C	4.3795083417	−0.1117747495	−0.1312878826
30	C	3.7101166910	2.4103756284	−2.8596469705
31	C	5.5295090311	−0.8125661102	0.5397678913
32	F	4.8751365528	2.2191080510	−3.5024888277
33	F	3.7248229820	3.6674656467	−2.3791978461
34	F	2.7293099608	2.3532759523	−3.7752855962
35	F	5.6762795136	−0.3979324671	1.8112375655
36	F	6.6970899867	−0.5958439256	−0.0894373896

37	F	5.3363551313	−2.1410007436	0.5854479827
38	S	0.7559832250	2.1626670988	1.9649523427
39	H	−5.2826874158	5.8305120473	0.6278211656
40	H	−6.2922031929	5.1708645934	1.9349908781
41	H	−6.7243477129	4.8341521480	0.2431787068
42	C	−4.4921504081	3.3094811226	−0.0371423092
43	H	−5.1542894172	4.8131649617	−1.4719421309
44	H	−3.9666248996	3.7831651269	−3.3661300380
45	H	−3.7286095220	1.7458277094	1.1388484180
46	H	−1.3042540983	0.2044720509	−4.0693562607
47	H	−1.0091441689	−0.9704462407	−1.9339149130
48	H	−1.4288524974	−8.0313438679	2.0844993867
49	C	−3.4954384393	−3.7156280083	1.9804375511
50	C	−2.7182319210	−2.5583385963	2.6298306634
51	H	−0.9746754920	−4.5357277491	−0.2238090889
52	H	−0.5892888742	−4.0098698691	2.7149656382
53	H	0.3040778131	−3.3396401027	1.3320510645
54	H	−3.5093321623	−4.7122457069	0.0515825237
55	H	−3.4383997973	−4.6171298528	2.5961640097
56	H	−4.5507645506	−3.4491320982	1.8906232040
57	H	−2.4040367516	−2.7776645343	3.6503904855
58	H	−3.2804617083	−1.6251419114	2.6431875301
59	H	−3.7941334003	−2.1683405630	−0.1644520361
60	H	−2.5734709870	−2.8403818751	−1.2344385817
61	H	−0.7828870698	−1.8718038152	−0.0609322939
62	H	−3.0823122636	−0.2298797565	1.0406331490
63	H	1.3664895720	1.6759554667	−1.7525645030
64	H	2.9331640459	−1.0495023549	1.1619563122
65	H	5.5978416031	0.9471851024	−1.5537099573
66	H	−0.8915464530	−1.6311728780	2.3368371338
67	H	−1.6336133830	1.0169118733	2.1316196540
68	H	2.0396890091	1.8376765504	1.7602217765

Table S8b Total energies in Hartree and optimized coordinates calculated in THF solvent using the SM8 method on wB97X−D/6−31G* level for neutral and protonated monomer and dimer THF molecules.

**monomer THF**				
E = −232.385827420250				
1	C	−5.8519654355	−0.5715759916	0.0787267442
2	C	−4.4011318608	−0.4005708892	−0.3766738800
3	C	−6.3633622476	0.8654207007	0.0035061775
4	C	−4.0450796191	0.9419451081	0.2569948024
5	O	−5.2468308664	1.7103680674	0.2794550746
6	H	−6.7522801481	1.0951751762	−0.9991615289
7	H	−7.1556682393	1.0798113300	0.7285315186
8	H	−6.4300480649	−1.2561580626	−0.5463524794
9	H	−5.8865599620	−0.9368496681	1.1109433708
10	H	−3.7416331639	−1.2081216809	−0.0503588383
11	H	−4.3488360148	−0.3338162231	−1.4689460202
12	H	−3.2841626878	1.4966001635	−0.3018882646
13	H	−3.6777076899	0.8081869696	1.2852243233
**monomer THF-H**				

E = −232.796755215918				
1	C	−5.9019052449	−0.5520742848	−0.1113871577
2	C	−4.3518483898	−0.4656339304	−0.1492025676
3	C	−6.4028555886	0.8536707602	0.1654851979
4	C	−3.9974691733	0.9862437278	0.1144461871
5	O	−5.2525183563	1.6886466955	−0.3113482747
6	H	−6.5368801026	1.0749060713	1.2253365066
7	H	−7.2607922463	1.1755886779	−0.4200336483
8	H	−6.2538703604	−1.2261924089	0.6699425481
9	H	−6.2855059268	−0.9133389578	−1.0656784011
10	H	−3.9776129659	−0.7798738931	−1.1235420374
11	H	−3.8904000970	−1.0982681351	0.6094226913
12	H	−3.8467314795	1.2202382936	1.1692824476
13	H	−3.2057337937	1.4023249481	−0.5037667612
14	H	−5.2965352750	2.6065834356	0.0186012694
**dimer THF**				
E = −464.775875891339				
1	C	−5.7524165887	−0.7454928609	−0.1258011307
2	C	−4.2479945255	−0.4322954949	0.0282533512
3	C	−6.4215172643	0.6361717910	0.0112055463
4	C	−4.2448006094	1.0485922547	0.4200934519
5	O	−5.3995249429	1.5982931269	−0.1952756824
6	H	−7.2073080322	0.8207904732	−0.7261912386
7	H	−6.8535059196	0.7574326176	1.0161754177
8	H	−5.9651486847	−1.1965626994	−1.0985037129
9	H	−6.1103630693	−1.4319328901	0.6456306079
10	H	−3.7598557204	−1.0543729605	0.7821961527
11	H	−3.7235205036	−0.5761008825	−0.9209415869
12	H	−3.3754692393	1.6044065010	0.0590011282
13	H	−4.3092179697	1.1544220782	1.5128734364
14	C	−6.8330153952	−0.7444559169	3.5194434679
15	C	−6.8501404522	−1.6556719061	4.7537395604
16	C	−5.5278618675	−2.4471699653	4.6234126815
17	C	−4.8515396191	−1.8114101204	3.3940747121
18	O	−5.4621000217	−0.5427092783	3.2212992873
19	H	−7.2913674574	0.2341566923	3.6799561833
20	H	−7.3411086112	−1.2301363415	2.6697178324
21	H	−6.8547797601	−1.0563609589	5.6681152671
22	H	−7.7321697181	−2.3003067440	4.7723073578
23	H	−5.6940624574	−3.5170819545	4.4759288751
24	H	−4.9106762076	−2.3288756969	5.5173053765
25	H	−5.0137370719	−2.4324303736	2.4985190701
26	H	−3.7770342908	−1.6526194902	3.5112395876
**dimer THF-H (protonated on the first THF)**				
E = −465.218345090135				
1	C	−6.2525138969	−0.1619841803	−0.8081284881
2	C	−4.8581947422	−0.7909017082	−0.8931601768
3	C	−6.0443042343	0.9843785232	0.1628152204
4	C	−4.3731836390	−0.7347017637	0.5433187929
5	O	−4.9912915185	0.4964505684	1.0819417723
6	H	−6.9073560139	1.2470614125	0.7747875441
7	H	−5.6377025761	1.8797714953	−0.3064038892

8	H	−6.9784117717	−0.8792040876	−0.4128322584
9	H	−6.6174966015	0.1945642429	−1.7725170042
10	H	−4.8770353248	−1.8165330906	−1.2647505402
11	H	−4.2042320605	−0.2021317593	−1.5422120292
12	H	−4.7389002388	−1.5716251250	1.1433583287
13	H	−3.2975347250	−0.6225072417	0.6667182088
14	C	−6.8678025505	−0.9895900442	3.2951265402
15	C	−6.2395342123	−2.1595118144	4.0338116749
16	C	−5.3446693253	−1.4516884702	5.0547448424
17	C	−4.7944527989	−0.2810396156	4.2551705308
18	O	−5.8472700985	0.0514745040	3.3006951570
19	H	−7.7450236004	−0.5897960314	3.8089706050
20	H	−7.1306659724	−1.2003658407	2.2557050451
21	H	−6.9912720852	−2.8033409214	4.4933900313
22	H	−5.6375657890	−2.7715980486	3.3542386509
23	H	−4.5467183398	−2.0887325758	5.4399156728
24	H	−5.9353243327	−1.0932914626	5.9030092381
25	H	−3.8966716969	−0.5541864379	3.6912490705
26	H	−4.5913331664	0.6141605125	4.8428260907
27	H	−5.3752616883	0.3269769603	2.0959523691
**dimer THF-H (protonated on the second THF)**				
E = −465.218286448533				
1	C	−4.9117432203	−1.2619237436	−0.5311487101
2	C	−4.0898612987	0.0118852042	−0.7477611792
3	C	−6.0769087783	−0.7531690420	0.3018316005
4	C	−4.2096804511	0.7132545699	0.5962417429
5	O	−5.5235406924	0.3327464473	1.1013199814
6	H	−6.8783888096	−0.3324375074	−0.3098791513
7	H	−6.5012075995	−1.4940230913	0.9834858182
8	H	−4.3299727608	−2.0048742359	0.0239738835
9	H	−5.2461409557	−1.7211887627	−1.4628839077
10	H	−4.5291453025	0.6245402966	−1.5406185275
11	H	−3.0492495904	−0.1889039047	−1.0083933173
12	H	−3.4551953931	0.3674147608	1.3098076763
13	H	−4.1829127439	1.8016454250	0.5426752994
14	C	−6.9402970350	−0.0216589502	4.0182327656
15	C	−7.1277366483	−1.2734799700	4.8557353310
16	C	−6.2795319359	−2.3161990124	4.1207352462
17	C	−5.0535619583	−1.5199539090	3.7189028725
18	O	−5.5686666960	−0.1550652003	3.4739302606
19	H	−7.6202492512	0.0275279384	3.1650702158
20	H	−6.9638574426	0.9150014565	4.5712445492
21	H	−8.1809711543	−1.5512263423	4.9177524559
22	H	−6.7545361185	−1.1155105495	5.8712685747
23	H	−6.0143345510	−3.1676088177	4.7492350957
24	H	−6.8065288775	−2.6932861789	3.2390184290
25	H	−4.5619302404	−1.8465908996	2.8019200646
26	H	−4.3192415723	−1.4248796666	4.5184369116
27	H	−5.5396749225	0.0928586853	2.4083380183
**dimer THF-2H (protonated on both THF)**				
E = −465.590126426655				
1	C	−5.9419823692	−0.0886651371	−0.9829816517

2	C	−4.4447624615	0.2366617417	−1.0474933711
3	C	−6.5537961715	1.1701380592	−0.4152422799
4	C	−4.1711644646	0.9528612645	0.2579389896
5	O	−5.5099871960	1.5842245415	0.5931719288
6	H	−6.6371550459	1.9812264361	−1.1383098071
7	H	−7.4725143100	1.0505648657	0.1537104738
8	H	−6.1331419722	−0.9399710931	−0.3246061133
9	H	−6.3636296998	−0.3136108172	−1.9634174270
10	H	−4.2248154020	0.8859093613	−1.8985567723
11	H	−3.8263771273	−0.6581575996	−1.1331344168
12	H	−3.9749374752	0.2953455127	1.1017568525
13	H	−3.4436099105	1.7607665806	0.2154792820
14	C	−6.5263898065	−1.9115233354	3.2575386538
15	C	−6.9753772910	−1.9989179721	4.6980573735
16	C	−5.7472188577	−1.5232171692	5.4823803325
17	C	−4.5907580302	−2.2044570331	4.7873282461
18	O	−5.0392380270	−2.2013347050	3.3401411265
19	H	−6.9499822517	−2.6454991384	2.5736672385
20	H	−6.5727256065	−0.9063949261	2.8444331830
21	H	−7.2352856404	−3.0274073925	4.9621394286
22	H	−7.8486433779	−1.3690546105	4.8718715254
23	H	−5.6431890513	−0.4362248741	5.4312467347
24	H	−5.7855709795	−1.8129074674	6.5332740106
25	H	−3.6429271190	−1.6721448141	4.7922123947
26	H	−4.4651935695	−3.2493324457	5.0699678386
27	H	−5.4562521275	2.5512940063	0.7016341398
28	H	−4.8045096585	−3.0349148388	2.8921310862
